# Precision Lignocellulosic Biorefinery: Process Regulation From Corn Stover to Products

**DOI:** 10.1002/advs.75391

**Published:** 2026-05-28

**Authors:** Xue‐Cheng Lin, Lan Wang, Tai‐Ran Pang, Ming‐Yuan Yin, Hong‐Zhang Chen

**Affiliations:** ^1^ State Key Laboratory of Biopharmaceutical Preparation and Delivery Beijing Key Laboratory of Biomass Refining Engineering Institute of Process Engineering Chinese Academy of Sciences Beijing China; ^2^ School of Advanced Interdisciplinary Sciences University of Chinese Academy of Sciences Beijing China

**Keywords:** biorefinery, cellulose nanocrystal, fermentable sugar, lignin protection, whole‐process regulation

## Abstract

Lignocellulosic biorefinery constitutes a critical pillar for transitioning the fossil‐based industrial paradigm toward sustainability. However, in lignocellulosic biorefinery, cross‐interference between cellulose, hemicellulose, and lignin persists throughout all steps. Effective regulation must extend beyond pretreatment across the entire process. Here, we develop a whole‐process regulation strategy for corn stover. Mechanical fractionation homogenizes physical structure, yielding parenchyma‐rich short fibers and vascular‐bundle‐dominant long fibers. For highly degradable short fibers, molecular control by methanol during steam explosion suppresses lignin condensation, followed by oxidative enhancement by carbon quantum dots during enzymatic hydrolysis, boosting cellulose conversion and facilitating mild lignin depolymerization for high‐performance epoxy resins. For high‐crystallinity long fibers, two‐stage selective enzymatic hydrolysis preserves crystallinity to produce cellulose nanocrystals. Techno‐economic analysis shows a 36.7% revenue increase over the unregulated baseline. This integrated approach embodies the concept of precision biorefinery: a transformative framework where whole‐process regulation orchestrates multi‐level heterogeneity‐guided fractionation to enable full‐component directed valorization, ensuring compatibility between biomass attributes, process, and product specifications. The concept and innovations have been industrially validated.

## Graphical abstract

This study presents a precision biorefinery framework that transforms corn stover into high‐value bioproducts through whole‐process regulation. Mechanical fractionation separates feedstock into parenchyma‐rich short fibers and vascular‐bundle‐dominant long fibers. For short fibers, methanol‐protected steam explosion and CQDs‐enhanced enzymatic hydrolysis preserve lignin reactivity. For long fibers, two‐stage enzymatic hydrolysis produces cellulose nanocrystals. This strategy ensures component‐specific valorization, achieving 36.7% revenue increase.

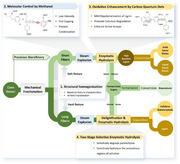

## 1 Introduction

Lignocellulosic biomass is the most abundant and carbon‐neutral organic resource on our planet [1, 2]. It predates humanity, and served as the principal source of materials and energy for most of human history. Nevertheless, its prolonged use did not substantially improve societal productivity. Instead, it was fossil resources that sparked the Industrial Revolution, providing―through refining―diverse, scalable, and affordable fuels, materials, and chemicals, thereby reshaping human civilization (Figure S1). However, both the oil crises of the last century and the increasing climate concerns have intensified the search for sustainable alternatives, revitalizing lignocellulose as a key renewable resource [3, 4]. Inspired by petroleum refining, lignocellulose refining aims to enable large‐scale production of fuels, chemicals, and materials from lignocellulosic biomass, facilitating sustainable industrial transition [5, 6].

**FIGURE 1:**
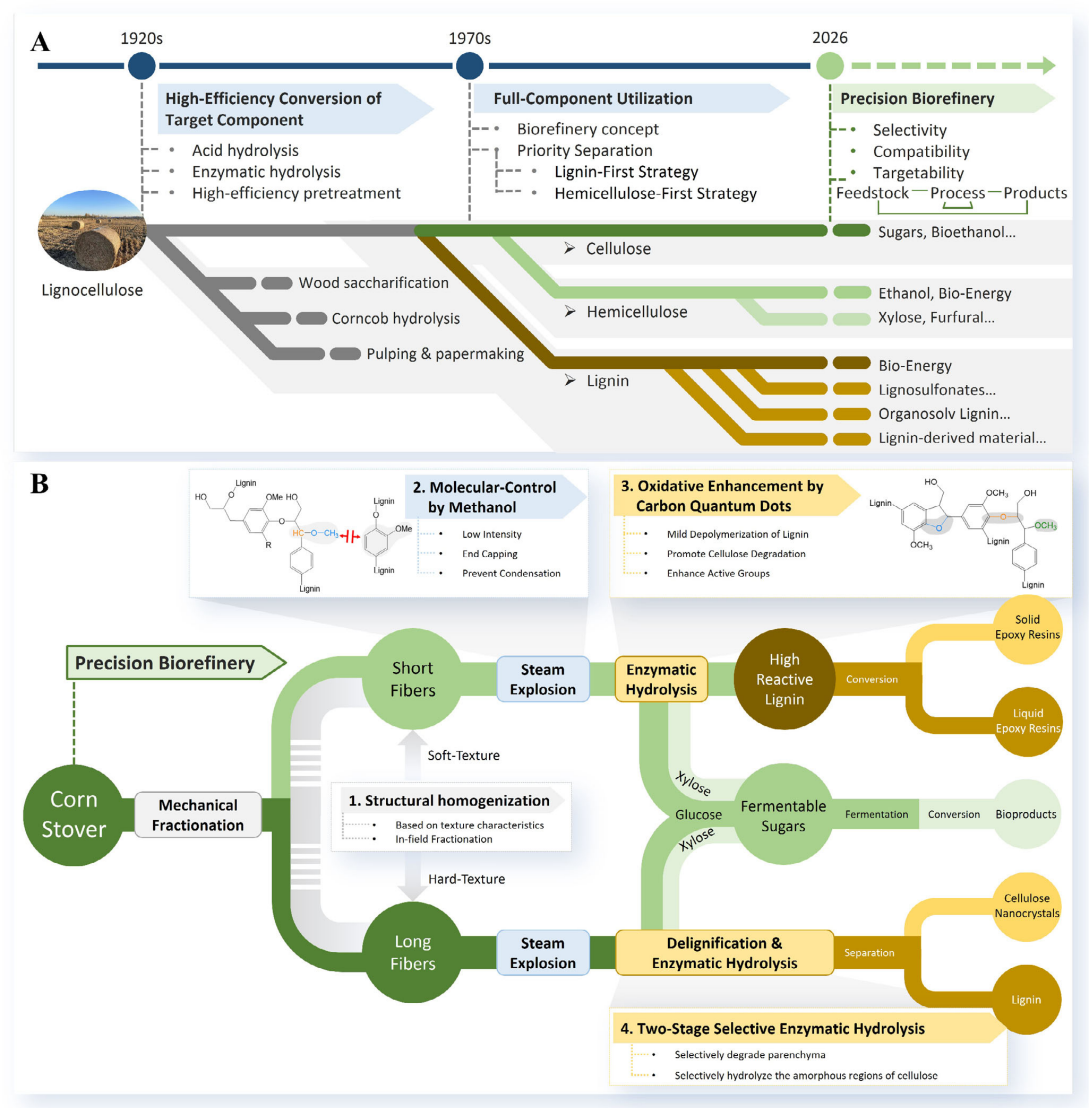
Advances in lignocellulosic biorefinery development and precision biorefinery. (A) Evolution of lignocellulosic biorefinery. In the 1920s, the first stage focused on high‐efficiency conversion of target components like cellulose and hemicellulose. Beginning in the 1970s, the second stage shifted toward full‐component utilization, marked by the application of lignin‐first and hemicellulose‐first strategies. By 2026, we have developed a precise biorefining framework that integrates lignocellulose valorization at both tissue and molecular levels, with precise regulation implemented throughout the entire process. (B) Precise lignocellulosic biorefining process transitioning from corn stover to high‐value products through precise process regulation.

The term “refining” originally meant “separating the valuable from the worthless”, first exemplified by metallurgy, removing impurities to obtain desired metals. Petroleum refining expanded this concept, shifting from targeting specific components to full‐component utilization—erasing the distinction between “target products” and “residues”. Similarly, lignocellulose refining has evolved through two stages. A century ago, countries such as Sweden and Germany, facing petroleum scarcity or embargoes, utilized abundant forest resources to develop acid hydrolysis processes for producing sugars and downstream products such as ethanol and single‐cell protein [7]. This marked the first stage of lignocellulosic refining, focused on efficient conversion of target components. However, strategies prioritizing only certain components while neglecting others proved economically unviable. This led to the emergence of the “full‐component utilization” strategy (Figure 1A). Building on earlier work focused on cellulose and hemicellulose, industries began to recognize the importance of lignin valorization [8]. Strategies such as “lignin‐first” biorefining—including solvent extraction [9] and reductive catalytic fractionation [10]—have gained prominence.

Whether employing lignin‐first or hemicellulose‐first strategies, the overarching goal remains effective fractionation of major components during pretreatment. This separation is central to the concept of full‐component utilization and has overemphasized the importance of pretreatment to the point of equating it with biorefinery itself. However, pretreatment alone is insufficient to achieve precise control over the conversion of components from raw biomass into final products, nor can it accomplish complete separation. Intertwining and mutual interference among components persist both before and after pretreatment. Therefore, biorefining requires continuous, deliberate regulation of all major components at every subsequent unit operation.

However, a significant misalignment persists between upstream and downstream steps in current biorefining practices. For instance, organosolv extraction can yield high‐purity lignin, but residual solvents may inhibit enzymatic hydrolysis [11]. Similarly, steam explosion disrupts biomass recalcitrant structure but can generate inhibitors that hamper subsequent enzymatic hydrolysis and fermentation [12], necessitating costly detoxification [13]. Furthermore, biorefining involves managing multiple components simultaneously—a challenge distinct from chemical reaction regulation, which typically focuses on a single target. Conventional optimization often overlooks “non‐target” components: severe hemicellulose‐first pretreatments may cause lignin condensation, reducing its reactivity [14], which diminishes lignin's valorization potential [15]. Similarly, enzymatic hydrolysis is conventionally regarded as targeting solely cellulose and hemicellulose, with little consideration given to concurrent lignin modulation. A comprehensive understanding of the structural evolution of all components, coupled with active regulation strategies, is essential to overcome the fragmentation between unit operations and enable fully integrated biorefining.

Moreover, current biorefining emphasizes molecular‐level fractionation, neglecting anatomical heterogeneity. Whether in lignin‐first strategies—such as DES or *p*‐toluenesulfonic acid pretreatment [16, 17]—or hemicellulose‐first like dilute acid pretreatment [18], or even direct enzymatic hydrolysis, the common practice involves grinding whole plants and then subjecting them entirely to molecular‐level processing. Even the most advanced engineering strategies currently either focus on the upstream genetic modification of feedstock traits [19] or rely disproportionately on the downstream engineering of microbial metabolic pathways [20], yet both completely neglect the fundamental prerequisite of macroscopic tissue heterogeneity. Similarly, recent cascade fractionation protocols relies on complex multi‐step solvent systems that still operate strictly at the molecular level, ultimately failing to overcome these anatomical barriers. [21]. Plant heterogeneity manifests first anatomically: the content, structure, and accessibility of cellulose, hemicellulose, and lignin vary significantly across tissues such as parenchyma and vascular bundles [22, 23]. This stands in stark contrast to petroleum, which—after millions of years of geological “natural pretreatment”—has lost its physical structure, forming a pseudo‐homogeneous hydrocarbon‐based liquid system suited to molecular separation (Figure S2). By directly adopting petroleum's refining model without accounting for anatomical heterogeneity, lignocellulosic biorefinery not only suffers from intense pretreatment requirements, inefficient delignification, and low enzymatic hydrolysis yields [24, 25], but also becomes disconnected from other utilization pathways such as pulping, pyrolysis, gasification, and solid‐state fermentation. Therefore, the neglect of anatomical structural heterogeneity and the misalignment between upstream and downstream steps necessitate a holistic perspective in biorefinery. Structural homogenization should precede molecular‐level conversion, and subsequent steps such as pretreatment, enzymatic hydrolysis, and further conversion should also be strategically tailored to the structural characteristics to ensure whole process compatibility.

In this study, we recognize that the inherent cross‐interference among cellulose, hemicellulose, and lignin across the entire process limits current biorefineries, and can only be resolved through whole‐process regulation rather than optimization of any single step. Therefore, we propose the concept of precision biorefinery and develop a highly selective and targeted whole‐process regulation strategy for corn stover, in which tissue‐level structural homogenization acts as the indispensable first step, precisely because the physicochemical properties and accessibilities of these three components are drastically different across distinct tissue structures. Mechanical fractionation was utilized to segregate the biomass into parenchyma‐rich short fibers and vascular‐bundle‐dominant long fibers. Following this initial structural homogenization, tailored whole‐process regulatory pathways were experimentally designed for each fraction: for the highly accessible short fibers, methanol‐mediated steam explosion coupled with CQD‐enhanced enzymatic hydrolysis was synergistically applied to overcome component antagonism, suppressing lignin condensation while promoting saccharification and simultaneous lignin activation; conversely, for the dense and high‐crystallinity long fibers, a two‐stage selective enzymatic hydrolysis was strategically designed to degrade amorphous regions while preserving crystalline domains, maximizing cellulose nanocrystal (CNC) production (Figure 1B).

## 2 Results

### 2.1 Structural Homogenization of Corn Stover Through Mechanical Fractionation Into Short Fibers and Long Fibers

**FIGURE 2:**
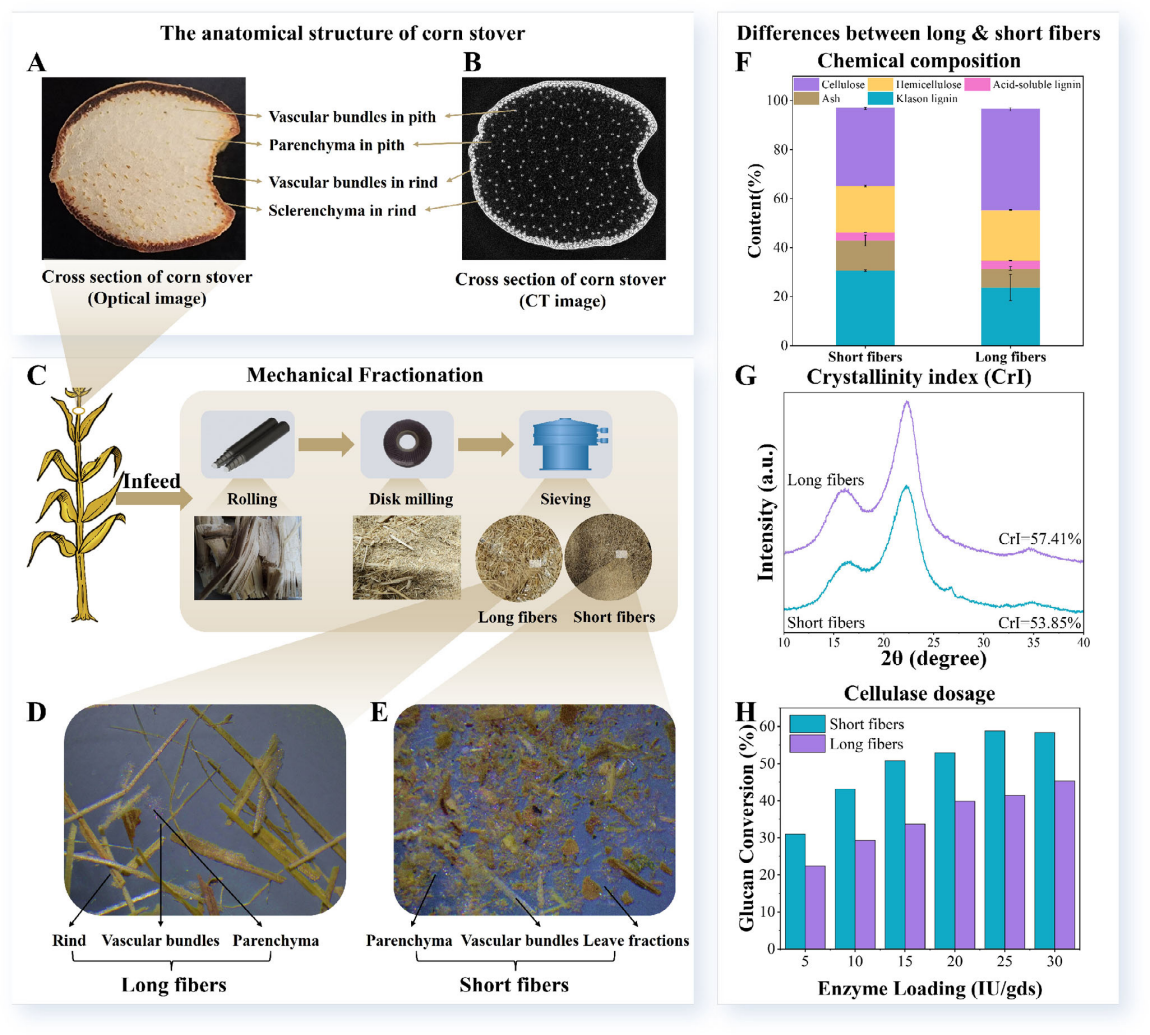
The anatomical structure and mechanical fractionation process of corn stover and the differences between long fibers and short fibers. (A, B) Optical image and CT scan image of corn stover cross section (CT: Computed Tomography). (C) Mechanical fractionation process of corn stover. (D, E) The composition of long fibers and short fibers. In long fibers, vascular bundles and rind account for the vast majority, while also containing a small number of larger parenchyma particles. In short fibers, parenchyma particles and leaf fragments make up the vast majority, while also including a small number of shorter vascular bundles. (F, G, H) Differences in chemical composition, crystallinity index, and cellulase dosage between long fibers and short fibers.

The synergistic, whole‐process regulation of the three major components is the core of precision biorefinery. Because the physicochemical properties and accessibilities of cellulose, hemicellulose, and lignin are drastically different across distinct tissues, structural homogenization by mechanical fractionation serves as the indispensable first regulatory step to achieve this core objective. Historically, however, this inherent heterogeneity of biomass has long been overlooked in conventional biorefinery process design, leading to non‐selective and inefficient blanket conversions [26]. In reality, all lignocellulosic biomass, including corn stover, derives from vascular plants with stems and leaves universally partitioned into vascular bundles and parenchyma (Figure 2A,B; Figure S3A,B). As fundamental structural units executing specialized functions, these tissues exhibit distinct morphological and physicochemical properties, resulting in vastly divergent industrial behaviors during fragmentation, permeation, and subsequent chemical reactions [27, 28].

Industrial implementation of complete vascular bundles and parenchyma separation is impractical due to their intricate interlocking within plant structures. A feasible approach lies in achieving substantial enrichment of each component. The distinct mechanical properties of vascular bundles and parenchyma, combined with the varying mechanical behavior of organs differing in their distribution ratios, provide a textural basis for mechanical fractionation. We have consequently developed a mechanical fractionation method [29] and corresponding industrial‐scale equipment based on the differential textural properties of distinct stover segments (Figure 2C). This approach incorporates a mechanical fractionation module designed to selectively enrich and isolate distinct structural components. We demonstrated that simple rolling‐disc milling and sieving effectively fractionated corn stover into two distinct fractions, which we functionally referred to as ‘long fibers’ and ‘short fibers’ based on their mechanical behaviors during processing rather than strict anatomical classifications. Long fibers, primarily composed of vascular bundles (Figure 2D) with higher cellulose content and crystallinity (Figure 2F,G). Short fibers, primarily composed of parenchyma particles (Figure 2E), exhibit a looser structure and higher lignin content (Figure 2F). Obviously, the distinct properties of long fibers and short fibers lead to different subsequent processing conditions. Under the same steam explosion conditions, short fibers exhibit a lower lignin molecular weight (Table S1), along with lower phenolic ‐OH content (Figure S4 and Table S2), indicating higher lignin reactivity. This heightened reactivity requires tailored control in subsequent steps to prevent undesirable condensation. Under the same enzymatic hydrolysis conditions, short fibers demonstrate higher glucan conversion (Figure 2H).

Therefore, it is necessary to fully redesign the entire biorefining process and product portfolio based on the fundamental differences between long fibers and short fibers. Short fibers have proven highly digestible for sugar production. One question, however, is whether lignin should be removed prior to enzymatic hydrolysis. Given the persistent challenges of solvent pollution and high cost in conventional delignification—and considering the inherently loose structure and high enzyme accessibility of short fibers—we opted for direct enzymatic hydrolysis after pretreatment. A central challenge in this route is to enhance lignin activity during both pretreatment and hydrolysis by promoting its depolymerization, increasing the exposure of active groups, and preventing condensation. For long fibers, the strategic focus shifts to leveraging their high crystallinity by selectively degrading the amorphous regions while preserving the crystalline cellulose. Building upon this structural homogenization, the highly accessible parenchyma structure of fractionated short fibers allowed for drastically reduced steam explosion severity. However, to concurrently manage lignin's structural evolution in this low‐severity acidic environment, precise molecular control must be applied.

### 2.2 Molecular Control by Methanol During Steam Explosion of Mechanical‐Fractionated Short Fibers

Following the mechanical fractionation that segregated the highly accessible parenchyma‐rich short fibers, the second crucial regulatory step focuses on disrupting their cellular network while strictly preventing the undesired condensation of lignin. As a structural intervention, steam explosion is a widely adopted pretreatment for overcoming lignocellulose recalcitrance and enhancing subsequent enzymatic digestibility [30]. However, conventional steam explosion faces an inherent trade‐off: excessive intensity causes severe lignin depolymerization and recondensation [31], while insufficient intensity fails to adequately disrupt the recalcitrant supramolecular structure. Although shortening the steam explosion time can reduce lignin degradation by limiting excessive breakage of aryl ether bonds and other structural features (Figure S5 and Tables S3 and S4), lignin in short fibers remains susceptible to unfavorable reactions in high‐temperature acidic environments [32]. Specifically, the removal of substituents from the Cα position on lignin's β‐O‐4 linkage triggers carbocation formation, which can initiate condensation reactions that compromise the structure and reactivity of lignin.

**FIGURE 3:**
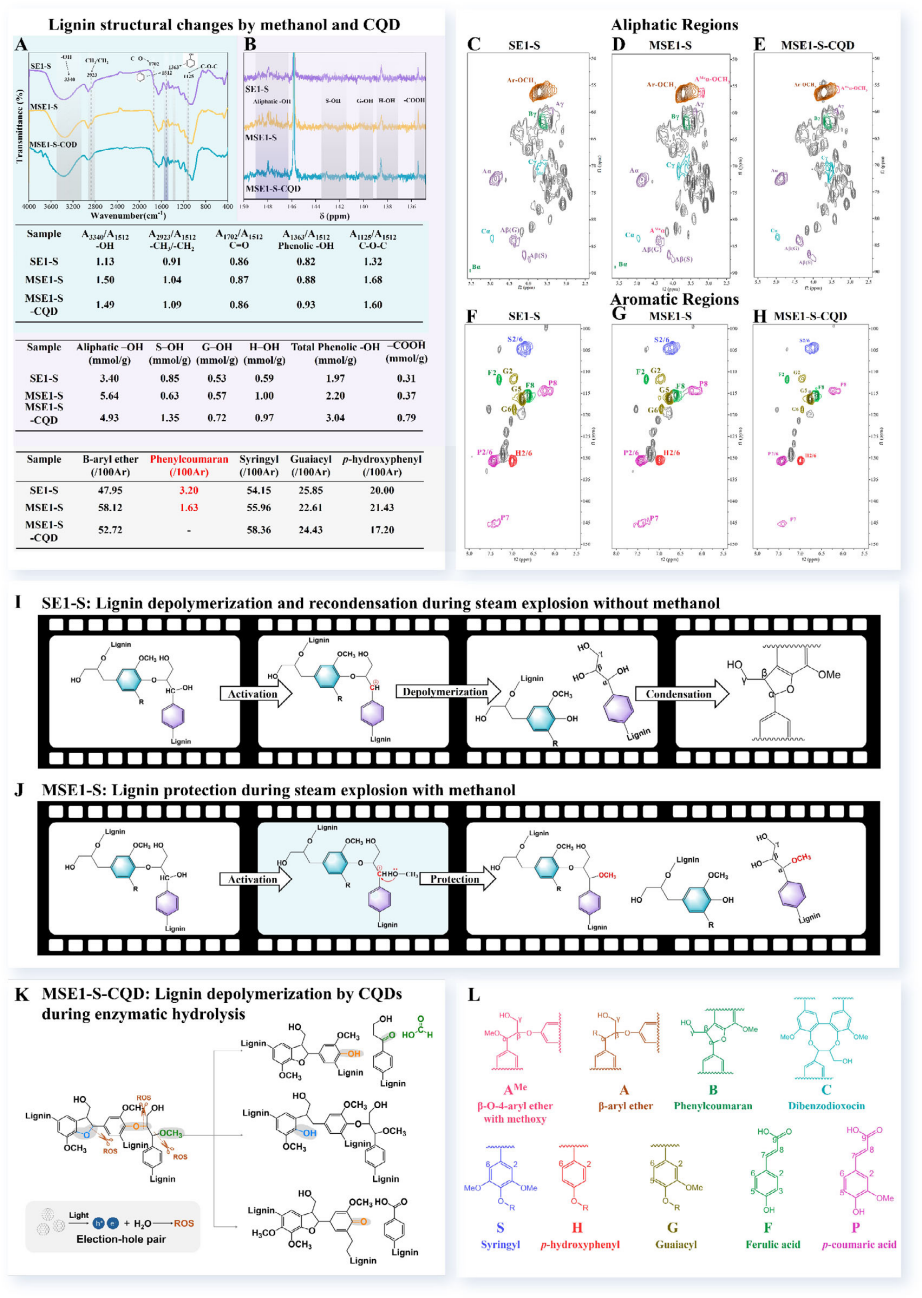
Molecular control by methanol during steam explosion and oxidative enhancement by carbon quantum dots during enzymatic hydrolysis of mechanical‐fractionated short fibers. (A) FTIR spectra and semi‐quantitative analysis comparing lignin structure before and after methanol protection during 1‐min steam‐explosion of short fibers and quantum dot enhancement during enzymatic hydrolysis (SE1‐S: 1‐min Steam‐Exploded Short Fibers; MSE1‐S: Methanol‐protected 1‐min Steam‐Exploded Short Fibers; MSE1‐S‐CQD: Carbon Quantum Dot‐Enhanced Lignin from Methanol‐Protected Steam‐Exploded Short Fibers). (B) Hydroxyl group quantification and semi‐quantitative analysis using ^31^P‐NMR spectroscopy comparing SE1‐S, MSE1‐S and MSE1‐S‐CQD. (C, D, E), Aliphatic region analysis using 2D‐HSQC NMR spectroscopy comparing SE1‐S, MSE1‐S, and MSE1‐S‐CQD. (F, G, H) Aromatic region analysis using 2D‐HSQC NMR spectroscopy comparing SE1‐S, MSE1‐S and MSE1‐S‐CQD, 58.12a/100Ar = 54.82 (A) + 3.30 (AMe). (I, J) Mechanistic schematic of lignin reaction pathway in steam explosion process without and with methanol addition. (K) CQD‐induced structural changes in lignin during enzymatic hydrolysis. L) Typical chemical bonds and monomeric structures of lignin.

To alleviate lignin condensation during pretreatment, we introduced methanol (6.78% v/w). By reacting at the Cα position of β‐O‐4 linkages to form ‐OCH_3_, methanol prevents carbocation formation and inhibits condensation. This resulted in unchanged lignin content (Figure S6) but relatively increased molecular weight, decreased polydispersity index (PDI) (Table S5), increased total ether linkages (FTIR, Figure 3A), elevated phenolic/aliphatic hydroxyl groups (^31^P‐NMR, Figure 3B), and enhanced aryl ether bonds (2D‐HSQC NMR, Figure 3C,D). These findings suggest methanol protects β‐O‐4 linkages by terminating Cα degradation pathways.

Conventional understanding attributes increased phenolic hydroxyl content to β‐O‐4 linkages cleavage [33]; simultaneous increases in phenolic ‐OH and β‐aryl ether bond content are unexpected. Contrary to this, methanol‐protected steam‐exploded lignin (MSEL), compared to lignin obtained from steam explosion without methanol (SEL), exhibits concurrent increases in phenolic ‐OH, β‐aryl ether bonds, and molecular weight. To elucidate this anomaly, we investigated changes in the content of phenylcoumaran structure, which provides a rough indicator of lignin condensation [34]. The MSEL showed a relative reduction in phenylcoumaran content from 3.20 to 1.63 compared to SEL (marked red in the table in Figure 3). Integrating this finding with increased β‐aryl ether bonds and molecular weight leads to a plausible explanation: Without methanol, hemicellulose‐derived acids during steam explosion protonates the α‐OH, forming a carbocation intermediate that triggers β‐O‐4 linkages cleavage. Cleaved fragments recondense under steam explosion conditions (e.g., forming condensed structures like phenylcoumaran via proposed pathways such as Cβ‐C5 coupling, Figure 3I). The reduction in phenylcoumaran content here serves as an indicator of an overall decrease in condensation, highlighting methanol's generalized protective role. With methanol, nucleophilic attack at the Cα carbocation grafts ‐OCH_3_ groups (confirmed at Cα in Figure 3C,D) or stabilizes the intermediate, preventing cleavage and recondensation (Figure 3J). This preserves phenolic ‐OH and explains the elevated molecular weight, β‐aryl ether, total ether, and phenolic ‐OH content. While methanol mitigates lignin depolymerization, it cannot completely prevent it. Reduced depolymerization lowers polydispersity versus unprotected lignin. Reduced β‐O‐4 cleavage also increases aliphatic ‐OH: methanol primarily etherifies Cα (forming Cα‐OCH_3_). Upon cleavage, the adjacent Cα‐OCH_3_ group sterically hinders Cβ‐OH condensation, and β‐OH's low reactivity prevents etherification, preserving β‐OH groups. Furthermore, this nucleophilic quenching mechanism is corroborated by recent solvent fractionations where polyols similarly attack carbocations, indirectly validating the efficacy of unhindered methanol as a scavenger [35].

We further found through literature comparison (Table S6) that adding small amounts of methanol during steam explosion protects lignin from depolymerization and recondensation via a unique mechanism, distinct from both conventional methanol‐free steam explosion and high‐temperature methanol solvolysis. We deduce that in acidic methanol‐free environments, Cα carbocations predominantly induce β‐O‐4 cleavage. With methanol addition, the pathway depends on nucleophilicity: Under strong nucleophilic conditions (high [MeOH], high temperature, catalyst), methanol attacks Cβ via SN2 [36], promoting β‐O‐4 cleavage and acting as a hydrogen‐donor to yield lignin oligomers. Conversely, under weak nucleophilicity (low [MeOH], moderate temperature), methanol is proposed to target Cα carbocations via SN1, hindering β‐O‐4 cleavage (Figure S7). Besides, from an engineering perspective, the ultra‐low methanol dosage remains far below explosion limits and will be co‐recovered with flash steam, robustly ensuring system safety during scale‐up.

### 2.3 Oxidative Enhancement by Carbon Quantum Dots During Enzymatic Hydrolysis of Steam‐Exploded Short Fibers

Building upon the methanol‐mediated steam explosion that successfully preserved the vulnerable β‐O‐4 linkages in the short fiber fraction, our whole‐process regulation strategy next elevates enzymatic hydrolysis from a simple saccharification step into a dual‐functional platform for concurrent, targeted lignin depolymerization. Conventional enzymatic hydrolysis, typically conducted under mild conditions (50°C, pH 4.8), effectively prevents further lignin condensation but completely fails to elevate the content of active functional groups in lignin for subsequent utilization. A significant challenge in precision biorefining is precisely regulating this lignin activation parallel to enzymatic saccharification without introducing toxic elements that impair fermentation. In our previous studies [37], we introduced a Fenton oxidation system to assist enzymatic hydrolysis, which improved substrate degradation and reduced cellulase consumption. This approach provided insight into how oxidation system can disrupt the intricate bonding structure of lignin, liberating active groups such as phenolic hydroxyls. However, traditional oxidation systems relying on metal ions frequently interact with lignin, occupying its active sites and impeding further valorization. Concurrently, the residual metal ions pose a significant challenge to downstream fermentation compatibility.

To overcome these limitations, we explored the use of lignin‐based carbon quantum dots (CQDs) as an alternative metal‐free oxidation system, leveraging their exceptional biocompatibility and oxidative capabilities [38]. Importantly, the preceding methanol‐mediated steam explosion preserved lignin's vulnerable β‐O‐4 linkages, providing abundant and specific target sites for this subsequent targeted oxidative cleavage. Thus, CQDs demonstrate remarkable potential to serve a dual function: promoting both lignin depolymerization and enhancing cellulose hydrolysis. CQDs were synthesized through the hydrothermal carbonization of MSE1‐S (Figures S8 and S9). The introduction of 10–5% (w/w) CQDs into the enzymatic hydrolysis system significantly increased glucan conversion (Figure S10). Within 24 h, it achieved a glucan conversion rate of 51%, representing 13% enhancement over the control group. This improvement can be attributed to the reactive oxygen species (ROS) generated by CQDs [38], which oxidatively cleave glycosidic bonds in cellulose, reducing its crystallinity and increasing enzymatic accessibility [39]. Furthermore, it is noteworthy that these lignin‐derived CQDs inherently lack the toxic heavy metals found in traditional oxidation systems. Combined with the extremely low working concentration (estimated at 5 ng/mL post‐separation), this ensures excellent downstream compatibility for subsequent microbial fermentation without introducing toxic inhibition.

Comprehensive structural analysis revealed intricate molecular transformations induced by CQDs treatment. The oxidative cleavage of cross‐linking bonds within lignin led to the formation of molecular fragments, resulting in a reduction of lignin's molecular weight (Figure S11). FTIR analysis (Figure 3A) indicated that while the core lignin structure remained intact, semi‐quantitative analysis revealed a decrease in ether and methoxy groups and an increase in phenolic hydroxyl content. The ^31^P‐NMR characterization (Figure 3B) showed a total phenolic hydroxy group content of 3.04 mmol/g in the CQDs‐enhanced, a value that exceeds previous reports of lignin treated with organic solvents or methylation. The content of guaiacyl (G‐OH) and syringyl (S‐OH) hydroxyl groups increased significantly, while *p*‐hydroxyphenyl (H‐OH) content remained stable, potentially indicating selective oxidation of H‐type units or conversion of lignin side‐chain alcohols to carboxyl groups [40].

The 2D‐HSQC NMR spectroscopy provided insights into the structural modifications induced by CQDs. The aliphatic region (Figure 3D,E) semi‐quantitative analysis revealed a decrease in β‐O‐4 linkages from 58.12/100Ar to 52.72/100Ar, with methoxy‐related signals disappearing, indicating nucleophilic reactions at the Cα position of β‐O‐4 bonds. The weakened Cα signal from the β‐5 phenylcoumaran structure and diminished C‐C bond signals between 65–80 ppm/2.5‐4.0 ppm further substantiated the oxidative depolymerization mechanism [38]. The aromatic region analysis (Figure 3G,H) showed an increase in S‐ and G‐ units, concurrent with a decrease in H‐type units—a finding consistent with ^31^P‐NMR observations (Figure 3B). Semi‐quantitative analysis of the 2D‐HSQC NMR results revealed an increase in S‐type units from 55.96/100Ar in MSE1‐S to 58.36/100Ar in MSE1‐S‐CQD, likely due to oxidative cleavage of aromatic rings. The S‐type units exhibited stability, likely due to protective side chains at the C3 and C5 positions that prevent condensation and stabilize the lignin structure. The results suggest that CQDs mediate proton and electron transfer, generating ROS that attack lignin's aryl ether bonds. This process releases hydroxyl groups through targeted cleavage of α‐O‐4 and β‐O‐4 linkages, exposing phenolic hydroxyls while oxidizing methoxy and hydroxyl groups to aldehydes or ketones, which further oxidize into carboxyl groups (Figure 3K) [41]. In summary, the CQD‐enhanced enzymatic hydrolysis step is shown to extend the regulation of all three components by simultaneously boosting cellulose saccharification and selectively liberating active lignin functionalities for targeted valorization.

### 2.4 Targeted Conversion of CQDs‐Modified Enzyme‐Hydrolyzed Lignin to High‐Performance Epoxy Resin

**FIGURE 4:**
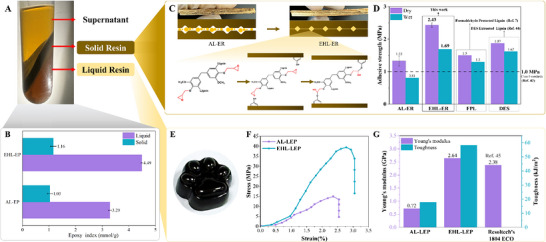
Targeted conversion of CQDs‐modified enzyme‐hydrolyzed lignin to high‐performance epoxy resins. (A) Photograph of centrifuged lignin‐based epoxy resin. (B) Epoxy index of alkali lignin‐based epoxy resin and enzymatically hydrolyzed lignin‐based epoxy resin (EHL‐EP: Enzymatically Hydrolyzed Lignin Epoxy Precursor, AL‐EP: Alkali Lignin Epoxy Precursor). (C, D) Schematic diagram of solid epoxy resin adhesive wood boards and their adhesive strength (EHL‐ER: Enzymatically Hydrolyzed Lignin Epoxy Resin, AL‐ER: Alkali Lignin Epoxy Resin). (E) Maleic anhydride curing effect on lignin‐based epoxy resin in cat claw molds. (F, G) The stress‐strain curve and Young's modulus, and toughness of cured liquid epoxy resins (AL‐LEP: Alkali Lignin Liquid Epoxy Resin, EHL‐LEP: Enzymatically Hydrolyzed Lignin Liquid Epoxy Resin). Significance was determined by t‐test, with asterisks indicating the following p‐values: ^*^ *p* < 0.05, ^**^ *p* < 0.01, ^***^ *p* < 0.001, ^****^ *p* < 0.0001. ns, not significant (p ≥ 0.05).

As the culmination of the whole‐process regulation for the short fiber fraction, the highly active lignin—whose reactivity was strategically preserved during steam explosion and subsequently enhanced via targeted CQD cleavage—is finally directed toward high‐value valorization. A key limitation in current industrial lignin application lies in its low reactivity, which typically mandates severe reaction conditions or toxic crosslinkers. Building upon our orchestrated preparation of this enzyme‐hydrolyzed lignin (EHL) with an elevated phenolic hydroxyl group content, we successfully explored its direct application in high‐performance epoxy resin production, completely bypassing the need for costly prior modifications or extensive depolymerization processes [42]. We epoxidized lignin and subsequently separated the liquid‐phase and solid‐phase epoxy resins using dichloromethane (Figure 4A) and examined the phenolic hydroxyl content of alkaline lignin (AL) and EHL (Figure S12). The EHL exhibited a higher phenolic hydroxyl concentration of 2.61 mmol/g, surpassing those obtained by alkaline (1.04 mmol/g) and DES methods (2.00 mmol/g) [43]. This increase in phenolic hydroxyl content suggests a potential for improved reactivity for epoxy synthesis. FTIR analysis corroborated these findings (Figure S13). Epoxy index measurements (Figure 4B) revealed that the liquid epoxy resin from EHL exhibited a substantially higher epoxide index of 4.49 mmol/g, compared to 3.29 mmol/g for the AL‐derived resin. This increase indicates enhanced crosslinking potential with epichlorohydrin. Conversely, the solid‐phase epoxy resins, composed of larger lignin molecules with more complex side‐chain structures, demonstrated lower epoxide indices: 1.16 mmol/g for EHL‐based resin versus 1.03 mmol/g for the AL‐based counterpart [44]. Yield analysis revealed a profound advantage for EHL, producing more liquid epoxy resin compared to conventional AL, resulting in a 31% increase in yield (Figure S14). This significant yield enhancement can be directly attributed to EHL's elevated phenolic hydroxyl content and the presence of low‐molecular‐weight lignin fragments, which facilitate more efficient epoxidation reactions. We applied the liquid‐phase lignin epoxy resin in curing reactions with maleic anhydride, and FTIR analysis confirmed the successful curing of both samples (Figure S15).

We further explored the potential of solid‐phase lignin epoxy resin as a wood adhesive (Figure 4C,D). FTIR analysis confirmed successful epichlorohydrin grafting and improved inter‐molecular interactions (Figure 4C; Figure S16). Building on the successful grafting and enhanced intermolecular interactions, the adhesive performance of the solid EHL‐based epoxy resin was outstanding, meeting Class I plywood requirements as specified in the GB/T 9846‐2015 standard (Figure 4D) [45]. It achieved a dry bonding strength of 2.43 MPa and a wet bonding strength of 1.69 MPa, significantly surpassing the AL‐based solid epoxy resin (Dry 1.33 MPa; Wet 0.81 MPa), as well as the results from formaldehyde protection (Dry 1.5 MPa; Wet 1.3 MPa) [9] and deep eutectic solvent (DES) extraction (Dry 1.87 MPa; Wet 1.62 MPa) [46] methods.

In a parallel investigation, we explored the mechanical characterization of the cured liquid resins (Figure 4F,G). The EHL‐based liquid resin demonstrated excellent molding properties, producing intricate and smooth finishes in silicone cat‐paw molding applications (Figure 4E). The EHL‐derived resin exhibited remarkable mechanical properties, with a maximum tensile strength of 39.47 MPa, strain of 3.05% (Figure 4F), and Young's modulus of 2.64 GPa (Figure 4G). These values significantly surpassed those of the AL‐based resin (14.91 MPa, 2.54%, and 0.72 GPa, respectively) and closely aligned with commercial standards, particularly Resoltech's 1804 ECO epoxy resin (tensile strength: 37.70 MPa, modulus: 2.38 GPa) [47]. Additional characterization revealed superior performance in water absorption, swelling, and thermal stability tests (Figures S17 and S18).

### 2.5 Two‐Stage Selective Enzymatic Production of Cellulose Nanocrystals From Mechanical‐Fractionated Long Fibers

**FIGURE 5:**
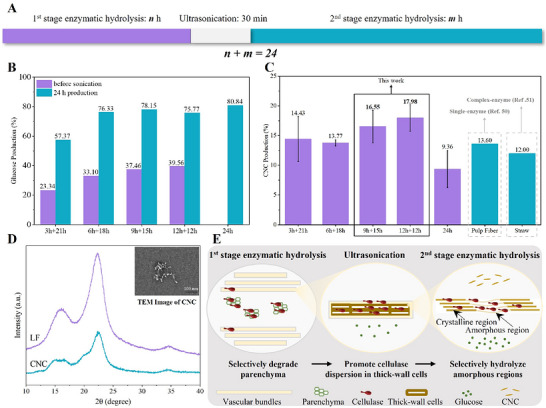
Two‐stage selective enzymatic production of cellulose nanocrystals from mechanical‐fractionated long fibers. (A) Operation of ultrasonication‐assisted two‐stage enzymatic hydrolysis. (B, C) Glucose production and CNC production under different two‐stage enzymatic hydrolysis strategies. (D) XRD spectra of bleached steam‐exploded long fibers and CNC prepared by 9 h+15 h enzymatic hydrolysis; TEM spectra of CNC prepared by 9 h+15 h enzymatic hydrolysis (LF: Long Fibers, CNC: Cellulose Nanocrystals). (E) Mechanistic flowchart of two‐stage selective enzymatic hydrolysis of long fibers.

Parallel to the regulation pathway of short fibers, the vascular‐bundle‐dominant long fibers acquired from the initial structural homogenization require an entirely different regulatory strategy tailored optimally to their dense tissue structure. Because these long fibers natively exhibit massive rigidity and significant crystallinity, they serve as a uniquely suitable substrate for cellulose nanocrystal (CNC) production, bypassing the need to rely on typical expensive feedstocks like cotton or microcrystalline cellulose [48]. While sulfuric acid hydrolysis is conventionally used to extract CNCs by dissolving amorphous regions, it generates massive acidic waste and inevitably destroys hemicellulose and lignin [49]. Adhering to our precision biorefining principles, we implemented an ultrasonication‐assisted two‐stage enzymatic hydrolysis approach, involving a sequential process: Stage 1 selectively degrades parenchyma; then, ultrasonication enhances cellulase penetration in vascular bundles’ thick‐walled cells; Stage 2 leverages cellulases’ inherent selectivity to hydrolyze amorphous regions, maximizing crystalline domain retention for CNC production (Figure 5A,E). We hypothesize that this approach could reduce the overall ultrasonication processing duration while mitigating potential enzyme deactivation, which requires precise regulation to balance CNC production and enzymatic hydrolysis efficiency [50].

The long fibers initially were pretreated by a purification process incorporating steam explosion, DES delignification, and hydrogen peroxide (H_2_O_2_) bleaching (Figure S19). Compositional and structural analyses of the pretreated fibers showed a significant cellulose purity increase to 83.0% (Figure S20), without compromising the crystalline structure. FTIR comparative analyses of DES‐extracted lignin and AL demonstrated minimal structural variations (Figures S21 and S22). Then, we precisely regulated ultrasonication‐assisted two‐stage enzymatic hydrolysis process exhibited distinct variations in CNC and glucose production efficiencies. Extended first stage hydrolysis durations correlated with increased residual content and decreased substrate conversion rates, likely due to early enzyme deactivation caused by prolonged ultrasonication exposure. Glucose production analysis substantiated these findings, showing that glucose production rates significantly increased after ultrasonication treatment (Figure 5B). In the first stage, 3 h of enzymatic hydrolysis yielded 23.34% glucose, while extending to 6 h increased the conversion to 33.10%. In the second stage, a 21‐h enzymatic treatment following ultrasonication achieved a moderate glucose production of 57.37%, indicating that ultrasonication's cavitation effect substantially contributed to enzyme deactivation, although cellulase activity was not entirely compromised. The 9h+15 h group demonstrated the highest glucan conversion rate, with minimal residual content (5.30%), followed by the 12h+12 h group (6.25%) (Figure S23). Continuous hydrolysis with ultrasonication resulted in higher residuals, indicating that ultrasonication‐assisted cellulase dispersion within vascular bundles, thereby promoting CNC production [51].

The CNC production data (Figure 5C) confirmed this speculation that the two‐stage process with 9h+15 h achieved a yield of 16.55%, outperforming the continuous 24 h group (9.36%), marginally trailing the 12h+12 h group (17. 98%). The 9h+15 h strategy was selected because it achieved the highest overall cellulose conversion rate and the lowest residue content (5.30%), maximizing total substrate utilization in the coproduction system. CNC production was improved relative to the single‐enzyme using pulp fiber (13.60%)[52] and the complex‐enzyme using bagasse (12.00%) [53]. Microscopic analysis corroborated this hypothesis, showing improved cellulose and enzyme distribution during hydrolysis (Figure S24). Furthermore, CNCs produced via the 9h+15 h group exhibited superior crystallinity index, zeta potential, and dispersion stability (Figures S25 and S26).

CNC crystallinity and crystalline morphology are critical properties linked to mechanical performance and thermal stability. XRD analysis of CNC produced through the 9h+15 h group (Figure 5D) demonstrated no significant changes in cellulose crystalline structure. Diffraction peaks at 2θ = 22.4° and 34.5° remained stable, with additional peaks at 2θ = 14.8° and 16.3° corresponding to cellulose I's (1, 2, 3, 4, 5, 6, 7, 8, 9, 10) and (110) planes. The CNC crystallinity increased to 83.0%, significantly higher than that of the raw material's 54.3% (Figure 5D), likely resulting from the effective removal of amorphous cellulose regions during enzymatic hydrolysis [51]. Thermogravimetric analysis indicated a slight decrease in thermal stability, attributable to the exposure of reactive surface groups during hydrolysis, but the overall thermal stability remained satisfactory (Figure S27).

### 2.6 Sustainability and Economic Analysis of Different Production Cases From Corn Stover to Products

**FIGURE 6:**
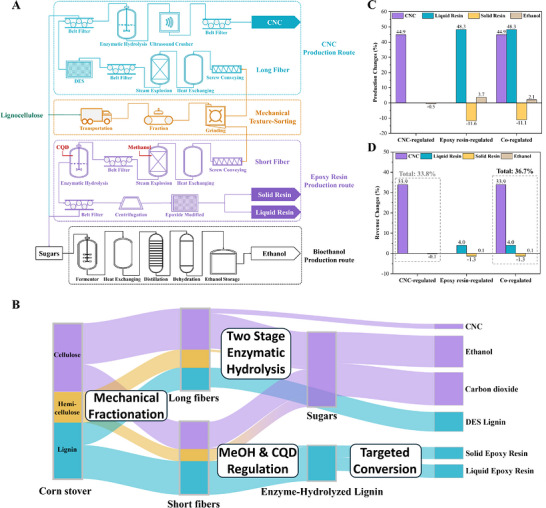
Sustainability and economic analysis of precision biorefinery from corn stover to products. (A) Process route for the co‐production of CNC, lignin‐based materials, and bioethanol from an annual input of 330 000 tons of corn stover. (B) Flow pathways of three components in the precise regulation processing from feedstock to products. (C, D) Variations in production and revenue changes for each product under regulated processes—specifically the CNC regulated process, epoxy resin‐regulated process, and coproduction process—evaluated against the unregulated multiproduct process as a baseline.

Ultimately, the superiority of whole‐process regulation strategy in precision biorefining must be validated from an economic engineering perspective. The development of lignocellulosic biorefining was initially driven by the demand for sustainable bioethanol [54]. To systematically evaluate the sustainability and economic viability of precision biorefinery, a comparative techno‐economic and environmental analysis was conducted for a process that annually converts 330 000 tons of corn stover into bioethanol as the main product, along with CNC and epoxy resins as co‐products. The system integrates four critical stages: mechanical fractionation, CNC production, lignin‐based epoxy resin synthesis, and bioethanol fermentation (Figure 6A). To evaluate the impact of fractionation and coproduction on economic and environmental performance, this assessment compares four cases—bioethanol‐only, CNC and bioethanol, lignin epoxy and bioethanol, and the full coproduction—against a non‐fractionated baseline (Table S7 and Figure S28). To further isolate the contribution of process regulation, it then evaluates the economic impact of individual process regulations by comparing another four cases—all sharing identical fractionation, using different combinations of measures such as two‐stage hydrolysis, methanol/CQDs only, and coregulation—against an unregulated baseline, while maintaining consistent sugar‐to‐ethanol conversion (Table S10).

Cost analysis indicates that CNC production and lignin‐based epoxy resin synthesis dominate total expenditures, accounting for 65.6% and 30.4%, respectively (Figure S29). Meanwhile, raw material costs constitute the majority of the total expenses (Figures S30 and S31). Ethanol‐only production without fractionation or process regulation is not economically viable. Fractionation followed by converting long fibers into CNC significantly enhances the system's profitability. Although the utilization of enzyme‐hydrolyzed lignin from short fibers for epoxy resins production can further increase revenue, the system remains unprofitable unless long fibers are utilized in a high‐value manner (Table S9). The coproduction system turns a loss from ethanol‐only production into a net profit and reduces CO_2_ emissions by 16.3%, achieving the highest profit per unit of CO_2_ emission (Figure S32) and underscoring the economic competitiveness of the fractionation‐based multiproduct strategy.

Precise regulation of the co‐production system yielded substantial improvements in economic performance. To evaluate this optimization, we assessed the economic efficiency across different scenarios (Unregulated process, CNC‐regulated process, epoxy resin‐regulated process, and co‐regulated process). Process regulation enhanced this efficiency metric and optimized the compatibility between raw material constituents and target products. The flow pathways of the three components demonstrated that regulated processing directed each component to its optimal product stream (Figure 6B). We validated the economic benefits of precision regulation in the multi‐product system through targeted regulation strategies (Figure 6C,D). The implementation of ultrasonication‐assisted two‐stage enzymatic hydrolysis (9h+15 h) to replace the conventional 24 h hydrolysis process, owing to a 44.9% increase in CNC yield, resulted in notable economic improvements (Figure 6C; Table S10). While this regulation slightly decreased bioethanol yields, it generated a 33.8% increase in overall co‐production revenue (Figure 6D; Table S10). Similarly, precision regulation in lignin‐based epoxy resin production showed substantial enhancement. Due to the introduction of methanol during steam explosion to protect lignin from condensation and the addition of carbon quantum dots during enzymatic hydrolysis to enhance the content of active functional groups, liquid epoxy resin yield increased by 48.3%, offsetting a modest 11.6% reduction in solid epoxy resin production (Figure 6C). When integrating precision control strategies for both CNC and epoxy resin production streams, the system achieved a substantial 36.7% revenue increase compared to the unregulated process (Figure 6D; Table S10), conclusively demonstrating the transformative potential of precision biorefinery approaches.

## 3 Discussion

Biomass utilization has developed over more than a century, with extensive efforts and numerous technologies developed from specialized perspectives. Yet, no fundamental breakthrough has been achieved. We attribute this bottleneck to the inherent cross‐interference and dynamic antagonism among cellulose, hemicellulose, and lignin—challenges that persist from raw material processing through pretreatment to downstream enzymatic hydrolysis and fermentation. Each unit operation involves intertwined processes affecting multiple components. Focusing solely on converting one component while neglecting structural changes in—or even damaging—the others, prevents any real technological advance in biorefining. Therefore, process regulation in biorefining must involve coordinated control over the transformation of multiple components in every unit operation. Recognizing this, we propose a whole‐process regulation strategy—coordinating the conversion of all three components consistently and sequentially from feedstock processing, pretreatment, and enzymatic hydrolysis to product formation. Due to the inherent structural heterogeneity of biomass, the content, structure, distribution, and accessibility of the three components vary across tissues such as vascular bundles and parenchyma. Thus, we first achieve structural homogenization of corn stover through mechanical fractionation, obtaining parenchyma‐rich short fibers and vascular‐bundle‐dominant long fibers. We integrate tissue‐level structural homogenization and molecular‐level separation and conversion—in contrast to conventional approaches that directly apply delignification, enzymatic hydrolysis, or other pretreatment technologies to fully crushed biomass without regard for the inherent structural heterogeneity of plant tissues [55, 56, 57]. Short fibers are highly degradable and well‐suited for producing sugars. However, a key question emerged regarding lignin utilization: should it be removed prior to enzymatic hydrolysis, or should direct enzymatic hydrolysis be applied to thereby obtain enzyme‐hydrolyzed lignin with maximized reactivity? Given that current delignification processes still face challenges such as high solvent pollution and cost, we opted for the direct enzymatic hydrolysis of short fibers following steam explosion pretreatment. Nevertheless, steam explosion tends to cause lignin condensation, which reduces its reactivity. To address this, we introduced molecular control by methanol during steam explosion of mechanical‐fractionated short fibers, functionalizing the Cα position of lignin side chains to suppress condensation. While lignin depolymerization remains essential for enhancing active functional groups like phenolic hydroxyls, conventional enzymatic hydrolysis focuses solely on cellulose conversion while neglecting lignin. Inspired by microbial oxidative degradation of lignin, we implemented oxidative enhancement by carbon quantum dots during enzymatic hydrolysis of steam‐exploded short fibers. This approach not only promoted cellulose degradation but also facilitated lignin depolymerization, yielding highly reactive enzymatically hydrolyzed lignin. To validate the high‐value potential of lignin derived from this whole‐process regulation, we further achieved targeted conversion of CQDs‐modified enzyme‐hydrolyzed lignin into high‐performance epoxy resin. As for long fibers, which exhibit higher crystallinity, we leverage this property to produce cellulose nanocrystals (CNCs). To selectively degrade the amorphous regions of cellulose and maximize crystalline content, we designed a two‐stage selective enzymatic production of CNCs from mechanical‐fractionated long fibers, thereby maximizing CNC yield. As a result, this integrated strategy transforms a conventional disjointed process into a synergistic and economically viable system, turning a loss from ethanol‐only production into a net profit with a 36.7% revenue increase over the unregulated multiproduct baseline, while reducing CO_2_ emissions by 16.3%.

These technological integrations articulate the essence of precision biorefining: a transformative framework where whole‐process regulation orchestrates multi‐level heterogeneity‐guided fractionation to enable multi‐component directed valorization, ultimately forging precise compatibility between biomass attributes, conversion pathways, and product specifications. Precision biorefinery is reflected in three principles: selectivity, compatibility, targetability (Figure S34). 1) Selectivity is dictated by inherent structural variations within plant materials, including tissue‐specific distinctions (e.g., vascular bundles vs. parenchyma) and molecular heterogeneity (e.g., cellulose, hemicellulose, lignin). The sequential processing strategy prioritizes tissue‐scale disassembly before undertaking differential molecular conversion. Structural homogenization is a critical initial step in all processing routes. 2) Compatibility requires that process design conform to material‐specific properties (e.g., using mild pretreatment for loose structures saves energy and reduces inhibitors), end‐product specifications (e.g., lignin product requirements for resins or phenolics influence process design), and upstream processing parameters that constrain downstream impacts (e.g., avoiding overly severe pretreatment or residual solvents that hinder subsequent processing). 3) Targetability entails preventing off‐target degradation, inactivation, or loss of critical components (e.g., through protection–deprotection strategies to preserve structural and chemical integrity) while promoting the efficient conversion of transformation‐critical constituents, such as the targeted hydrolysis of cellulose amorphous regions. These principles can further serve as foundational rules for machine learning‐based optimization and virtual process design of lignocellulosic biorefineries.

The concept of precision biorefinery, validated here with corn stover, offers a broadly extensible framework for the valorization of diverse lignocellulosic biomass. Industrial relevant feedstocks, particularly gramineous plants, share a common structural and chemical mode—comprising cellulose, hemicellulose, and lignin at the molecular level, and vascular bundles and parenchyma at the tissue level. This commonality necessitates whole‐process regulation of all major components. Structural homogenization through mechanical fractionation serves as the critical initial step, which not only enriches specific constituents but also supplies uniform feedstocks for downstream processing, whether employing lignin‐first, hemicellulose‐first, enzymatic, or thermochemical pathways. Importantly, in strategies targeting specific components such as lignin or hemicellulose, it is essential to concurrently manage structural evolution and conversion efficiency in non‐target components to maintain overall process compatibility. Ultimately, the implementation of precision biorefinery demands that conversion routes and target products be strategically aligned with the distinct molecular characteristics—such as cellulose crystallinity and lignin reactivity—inherent to each biomass fraction. Through precision biorefinery, we strive to establish an industrial system that maximizes the potential of lignocellulose—ushering in a renewable‐resource‐led industrial revolution, much as coal and petroleum did centuries ago.

## 4 Conclusion

Our research presents a precision biorefinery framework that reconstructs conventional biomass conversion strategies. By integrating mechanical fractionation, methanol‐protected steam explosion, and CQDs‐enhanced enzymatic conversion, we demonstrate that systematic process regulation can effectively address the inherent heterogeneity of lignocellulose while maximizing component valorization. The remarkable improvements including enhanced β‐O‐4 bond preservation, superior lignin reactivity for high‐performance epoxy resins, elevated yields of fermentable sugars, and high‐crystallinity CNC production. These advancements collectively enable a 36.7% increase in revenue compared to the unregulated baseline.

## 5 Experimental Section

### 5.1 Raw Materials and Reagents

Corn stover was obtained from Beijing, with leaves and roots removed. Cellic CTec3 cellulase (150 FPU/mL) was purchased from Novozymes (China). All other reagents were of analytical grade and purchased from Aladdin Biochemical Technology Co., Ltd. (Shanghai, China).

### 5.2 Mechanical fractionation of Corn Stover

Corn stover was processed using a mechanical texture‐sorting approach. The material was subjected to five passes through a rolling‐disc milling (LFS‐SJ3FE1.7, GeDun ZhiDao Equipment Co., Ltd., China), ground five times using a disk mill (MF‐3000, Guangzhou JunGong Mechanical Equipment Co., Ltd., China), and sieved for 1 min (ZDS200, Quanzhou Oulandi Technology Co., Ltd., China). This process separated the material into short fibers (< 20 mesh) and long fibers (> 20 mesh) [29]. The cross‐section of corn stover was macroscopically observed and photographed, followed by scanning with a computed tomography (CT) scanner (AX‐2000CT, Always Imaging Co., Ltd., China). Long fibers and short fibers were observed via a stereomicroscope (T2‐HD206, Shenzhen AOSVI Optical Instrument Co., Ltd., China) (Supplementary Methods). The Three‐Component Content and X‐ray Diffraction (XRD) analysis of long fibers and short fibers are described in the Supplementary Methods.

### 5.3 Steam Explosion Pretreatment of Long Fibers and Short Fibers

A steam explosion was performed on corn stover (unclassified/long fibers/short fibers). Corn stover was mixed with distilled water (2:1 mass ratio) and loaded into a 2 L steam explosion system. The equipment consists of three units—steam generator, reaction chamber, and reception chamber—interconnected and precisely controlled by ball valves [58]. Samples were subjected to saturated steam at 1.0 MPa (185‐190°C) for 1 and 10 min, followed by rapid depressurization and air‐drying [30].

### 5.4 Methanol‐protected Steam Explosion Pretreatment of Short Fibers

The methanol‐protected steam explosion pretreatment was performed on corn stover (unclassified/short fibers). A 10% methanol solution (400 mL) was mixed with 200 g of dried short fibers and loaded into a 2 L steam explosion reactor. The samples were exposed to saturated steam at 1.0 MPa (185‐190°C) for 1 and 10 min, followed by rapid depressurization and air‐drying [30].

### 5.5 Carbon Quantum Dots Enhanced Enzymatic Hydrolysis of Short Fibers

Carbon quantum dots were synthesized from steam‐exploded short fiber enzymatic lignin (Supplementary Methods). Ten grams of short fibers were suspended in 200 mL acetate buffer (0.05 M, pH 4.8) and supplemented with 1 µg of carbon quantum dots. The mixture was irradiated for 2 h, followed by enzymatic hydrolysis with 20 FPU/g dry matter (DM) cellulase for 48 h. Solid‐liquid separation was performed by centrifugation (10,000 rpm, 10 min) [28]. The residual lignin was subsequently used to synthesize lignin‐based epoxy resins (Supplementary Methods).

### 5.6 Two‐Stage Selective Enzymatic Hydrolysis of Long Fibers

The purification process was applied to remove lignin using a choline chloride‐lactic acid deep eutectic solvent (DES) (1:2 molar ratio) at 120°C for 9 h, with a solid‐to‐liquid ratio of 1:10. After treatment, the long fiber solid residue was washed and dried, while lignin was precipitated from the liquid phase using distilled water. The delignified fibers were then bleached with a 10% H_2_O_2_ solution at 50°C and 150 rpm for 12 h, followed by filtration and freeze‐drying.

CNCs were prepared using a ultrasonication‐assisted two‐stage enzymatic hydrolysis. One gram of bleached long fibers was dispersed in citric acid buffer (5% solid content) and treated with 20 FPU/g dry matter of Cellic CTec3 cellulase for *n* hours. The mixture was then ultrasonication‐enhanced at 800 W for 30 min. After sonication, enzymatic hydrolysis continued for *m* hours, ensuring a total of *n*+*m* = 24 h. Solid‐liquid separation was performed at 10,000 rpm for 5 min. The liquid phase consisted of a sugar‐rich solution, and the solid was re‐dispersed in water, centrifuged at 2000 rpm for 5 min. The supernatant was collected and freeze‐dried to obtain CNC, while the remaining solid was denoted as residue. The production of glucose or CNC is derived by dividing the weight of the obtained product by the weight of the bleached long fibers.

### 5.7 Polysaccharides/Sugars Characterization

The glucan conversion rate, representing the total concentration of glucose and xylose, was used to evaluate the enzymatic hydrolysis efficiency. The concentrations of glucose and xylose were quantified using high‐performance liquid chromatography (HPLC, Agilent 1200, USA) equipped with an Aminex HPX‐87H column (Bio‐Rad, Hercules, CA) at 40°C, with a mobile phase of 5 mmol/L H_2_SO_4_ flowing at 0.6 mL/min [58]. The glucan conversion rate was calculated using the following equation:

$$Glucan\;Conversion\;rate\;\left(\%\right)=\frac{C\times V\times 0.9}{M}\;\times 100\%$$

where 𝐶 is the glucose concentration in the enzymatic hydrolysis solution, 𝑉 is the volume of the hydrolysis solution, and 𝑀 is the mass of glucan in the substrate.

### 5.8 Lignin/Lignin‐Epoxy Resin Characterization

FTIR spectra of samples were recorded over the range 4000–500 cm^−1^ using a Nicolet iS20 FTIR spectrometer (Thermo Fisher Scientific, USA).

The ^31^P‐NMR and 2D HSQC NMR of lignin were recorded using a Bruker AVIII 600 MHz NMR spectrometer (Bruker, Germany). ^31^P‐NMR was employed for quantitative analysis of whole hydroxy and carboxyl groups, while 2D HSQC NMR was used to analyze the chemical structure of lignin before and after grading. Samples were dissolved in 0.6 mL DMSO‐d6, and standard pulse sequence hsqcedegpsisp 2.2 was used for scanning [40, 59].

The phenolic hydroxyl content in lignin (enzymatic and alkaline lignin) was determined by differential UV spectroscopy using a P6 UV‐VIS spectrophotometer (Shanghai Mapada Instruments Co., Ltd., China). Thirty milligrams of lignin were dissolved in 50 mL of 0.1 mol/L NaOH solution to prepare the lignin solution. 0.5 mL of the solution was transferred and made up to 10 mL with either 7.0 mol/L NaOH or 0.25 mol/L HCl to prepare alkaline and acidic solutions, respectively. The alkaline solution was scanned and the absorbance at 250 nm was recorded, using the acidic solution as the reference. The phenolic hydroxyl content was calculated using the following formula:

$$\;\Delta A_{max}=5.6\times 10^{3}cl+0.21$$

where ΔA_max_ is the absorbance at 250 nm, c is the phenolic hydroxyl concentration (mol/g), and l is the path length (1 cm) [60].

The preparation of Lignin‐Based Epoxy Resin was conducted following the Supplementary Methods. Solid resin was mixed with water and coated on pine wood slices, followed by hot pressing. The adhesive strength was measured according to the GB/T 17657‐2022 standard [61]. Liquid epoxy resin was mixed with maleic anhydride at 60°C for thermal curing (140°C for 3 h). After curing, the tensile properties of the resin samples were measured using a universal tensile testing machine (STD5000, Xiamen Yishite Instruments Co., Ltd., China).

### 5.9 Sustainability and Economic Analysis of Precision Biorefinery Processes

The technical and economic feasibility of the precision biorefinery was evaluated through techno‐economic analysis (TEA) and life cycle assessment (LCA). The process route model was developed using SuperPro Designer software (V10 Build 7, Intelligen Inc., USA) to evaluate the technical and economic feasibility of four‐stage workflow: corn stover fractionation, lignin‐based epoxy resin synthesis from short fibers, CNC production from long fibers, and bioethanol fermentation. The specific process parameters, and associated costs for this model are provided in Tables S7 and S8, respectively. The LCA model was conducted following the principles and framework outlined by the International Organization for Standardization (ISO 14040) [62]. The analysis was divided into four main phases: goal and scope definition, inventory analysis, impact assessment, and interpretation of results.

The corn stover fractionation stage involved transportation, washing, mechanical disc‐rolling, and sieving. For short fiber processing, short fibers were mixed with a 10% methanol solution (2:1 mass ratio) and steam‐exploded at 1.0 MPa for 1 min, achieving partial hydrolysis (5% cellulose, 10% hemicellulose). To improve enzymatic selectivity and maximize yield of CNC, a two‐stage enzymatic hydrolysis strategy was employed. Substrates were first hydrolyzed (50 °C, 9 h, 10% solids, 20 FPU/g DM, 150 rpm), then subjected to ultrasonication treatment (800 W, 30 min), followed by a secondary enzymatic hydrolysis for different durations (e.g., 6, 15 h) without additional enzyme loading. The ultrasonication step enabled cellulase dispersion in vascular bundles, thereby enhancing enzymatic accessibility and leaving crystalline regions intact for CNC preparation. After hydrolysis, the solids were separated via plate‐frame filtration and reacted with sodium hydroxide, epichlorohydrin, and aniline (98°C, 1.5 h) to synthesize epoxy resin, while liquids were directed to fermentation. For long fiber processing, long fibers were rehydrated (2:1 water‐to‐fiber ratio), steam‐exploded (1.0 MPa, 10 min), delignified, and bleached. Bleached long fibers were two‐stage enzymatically hydrolyzed (50°C, 9 h, 10% solids, 20 FPU/g DM, 150 rpm), ultrasonicated (800 W, 30 min), and subjected to a secondary hydrolysis (15 h, no additional enzymes) to yield CNC (Supplementary Methods, Table S7). The bioethanol production process included sugar concentration, fermentation (72 h, ammonium sulfate as nitrogen source), and distillation (95% ethanol), followed by molecular sieve dehydration (>97% purity).

Three biorefinery configurations were analyzed: 1) bioethanol production route, which retained only the transportation, washing, and crushing steps of corn stover fractionation, followed by steam explosion pretreatment (1.0 MPa, 10 min) and direct enzymatic hydrolysis (50°C, 96 h, 10% solids, 20 FPU/g DM, 150 rpm). The hydrolysate was concentrated and processed for ethanol production. 2) The CNC and bioethanol coproduction route, which integrated the CNC production stage, with unutilized long fibers enzymatically hydrolyzed under the same conditions as the bioethanol‐only route and directed to ethanol production. 3) The lignin‐based epoxy resin and bioehanol coproduction route, which retained the epoxy resin synthesis stage, with residual short fibers enzymatically hydrolyzed under identical conditions and processed for ethanol production. In both coproduction routes, unutilized fibers were converted to ethanol following the same enzymatic hydrolysis as the bioethanol route (Figure S28).

### 5.10 Statistical Analysis

Experimental data were analyzed in triplicate and expressed as mean values ± standard deviation. Statistical analysis was performed to determine significant differences in selected data using Origin 8.0 (OriginLab, USA).

## Conflicts of Interest

The authors declare no conflicts of interest.

## Supporting information




**Supporting File**: advs75391‐sup‐0001‐SuppMat.docx.

## Data Availability

The data that support the findings of this study are available in the supplementary information of this article.
