# Supplementary material for: Precision Lignocellulosic Biorefinery: Process Regulation From Corn Stover to Products
Source: Adv Sci (Weinh). 2026 May 28;13(40):e75391. doi: 10.1002/advs.75391 (PMC13335572; doi:10.1002/advs.75391)
Supplement: Supplementary file 1 — Supporting File: advs75391‐sup‐0001‐SuppMat.docx. [file ADVS-13-e75391-s001.docx]

Supporting Information

**Precision Lignocellulosic Biorefinery: Process Regulation from Corn Stover to Products**

*Xue-Cheng Lin, Lan Wang*, Tai-Ran Pang, Ming-Yuan Yin, and Hong-Zhang Chen*

**Contents**

Supplementary Methods 2~6

Supplementary Figures 7~40

Supplementary Tables 41~55

References 56

**Supplementary Methods**

1. **Imaging of corn stover, long fibers, and short fibers**

Macroscopic observation of corn stover：The third to fourth internode of naturally air-dried corn stover was cut into 3-cm-long segments, ensuring smooth cross-sectional surfaces. Imaging was subsequently performed using a smartphone (Galaxy S22 Ultra, Samsung Electronics Co., Ltd., South Korea) at 1× optical magnification.

Optical microscopy of corn stover: The corn stover was cut into 1-cm-long segment, immersed in Formaldehyde-Acetic Acid-Alcohol (FAA) fixative solution, dehydrated through an ethanol gradient, cleared with xylene, and then embedded in paraffin. Sections were taken both transversely and longitudinally and observed under an optical microscope (BX 41, Olympus (China) Co., Ltd., China) at 100× magnification.

Scanning electron microscopy (SEM) of the cross-section of corn stover: The sectioned sample was mounted on conductive adhesive tape, sputter-coated with gold, and subsequently observed with a field-emission scanning electron microscope (JSM66700F, JEOL, Tokyo, Japan).

Computed tomography (CT) scanning of corn stover: Scanning was performed under 180 kV and 0.5 mA, achieving a final isotropic voxel size of 8 μm.

Observation of long fibers and short fibers via stereomicroscope: A small amount of long fibers and short fibers were placed on the sample stage, brought into sharp focus, and imaged. Their origins were investigated by analyzing morphological characteristics.

1. **Carbon quantum dots synthesis**

Synthesis of carbon quantum dots (CQDs) involved dispersing 0.1 g of steam-exploded short fibers enzymatic lignin in 100 mL water and treating it in a high-pressure reactor at 200℃ for 12 hours. The mixture was separated by filtration using a Büchner funnel. The filtrate was dialyzed for 48 hours using a 500 Da dialysis membrane, with water replaced every 12 hours. The solid was then stored, and its concentration determined by weight loss after drying^[1-2]^.

1. **Extraction of Enzyme-Hydrolyzed Lignin**

Ten grams of ball-milled corn straw were suspended in 200 mL acetate buffer and hydrolyzed with 20 FPU/g DM Cellic CTec3 cellulase at 50℃ and 150 rpm for 48 hours. Solid-liquid separation was performed by centrifugation (10,000 rpm, 10 minutes), followed by washing and freeze-drying the residual lignin^[3]^. The dried lignin was subjected to additional ball-milling for 2 h, followed by enzymatic hydrolysis under identical conditions, then washed and freeze-dried to yield enzyme-hydrolyzed lignin (EHL).

1. **Extraction of Methanol-Protected Steam-Exploded Short Fibers Lignin**

A similar protocol was used, with 10 g methanol steam-exploded corn straw short fibers powder mixed with 1 μg carbon quantum dots, irradiated for 2 hours, and then hydrolyzed in acetate buffer (pH 4.8) with 20 FPU/g DM cellulase for 96 hours^[3]^. Solid-liquid separation was performed by centrifugation (10,000 rpm, 10 minutes), followed by washing and freeze-drying the residual lignin.

1. **Alkaline Lignin Extraction**

Twenty grams of corn straw short fibers powder were treated with 2.0% (w/v) NaOH solution at 121℃ for 90 minutes. The cooking liquor was filtered (200 mesh), acidified to pH 2 with hydrochloric acid to precipitate lignin, and then washed to neutral pH and freeze-dried.

1. **Preparation of Lignin-Based Epoxy Resin**

Following the modified method of Verdross *et al.*, three grams of lignin was dissolved in 30 mL of 5.6 mol/L NaOH solution, and 30 mL of epichlorohydrin and 0.65 mL aniline were added. The mixture was stirred at 98℃ for 1.5 hours. After neutralization with 25 mL of deionized water and cooling to room temperature, 10 mL of ethyl acetate was added to obtain lignin-based epoxy resin^[4]^. The epoxidized lignin was subsequently separated into liquid-phase epoxy resins (Liquid Resin) and solid-phase epoxy resins (Solid Resin) using dichloromethane.

1. **Characterization**

Three-Component Content: The cellulose, hemicellulose, and lignin contents were determined following the National Renewable Energy Laboratory (NREL) NREL/TP-510-42618 (Determination of Structural Carbohydrates and Lignin in Biomass) and the resulting data were normalized^[5]^.

Gel Permeation Chromatography (GPC): The molecular weight distribution of lignin was measured by high-performance liquid chromatography (HPLC, Agilent 1200, Agilent Technologies Inc., USA) with a hydrophilic gel column (TSKgel G3000PWxl, TSK Corporation, Japan). The mobile phase was a tris(hydroxymethyl)aminomethane solution (pH 7.4), with a column temperature of 25℃, a flow rate of 0.5 mL/min, and a detection wavelength of 280 nm.

Lignin sample preparation: 10 mg of EHL was dissolved in 0.1 mL of 0.5% (w/v) NaOH solution under sonication. After complete dissolution, the mixture was diluted with 2 mL of mobile phase and filtered through a 0.22 μm membrane into a sample vial for molecular weight determination.

UV-Vis Spectroscopy: The absorption spectra of carbon quantum dots synthesized by hydrothermal methods were measured using a UV-Vis spectrophotometer (P6, Shanghai Meipuda Instruments Co., Ltd., China). Fluorescence properties of the carbon quantum dots were observed under UV.

X-ray Diffraction (XRD): XRD measurements were conducted using an X-ray diffractometer (Panalytical Empyrean, Netherlands) with a rotating anode X-ray generator. The crystallinity of the cellulose in CS and SECS was determined as follows:

$$The crystallinity of cellulose=\frac{I_{200}-I_{am}}{I_{200}}$$

Where *I*_200_ is the height of the 200 peak (2*θ* = 22.5°) and *I*_am_ is the minimum between the 200 and 110 peaks (2*θ* = 18°).

Optical Microscopy: The morphology of the substrate fibers during enzymatic hydrolysis was observed using an optical microscope (BX 41, Olympus, Japan). The cellulose samples treated with cellulase were stained with eosin (Eosin Y), and the stained samples were examined under the microscope.

Transmission Electron Microscopy (TEM): The surface morphology of CNC was observed using a low-voltage transmission electron microscope (JEM1200EX, JEOL, Japan). The freeze-dried CNC powder was dispersed in a 0.01 wt% aqueous solution and subjected to ultrasonic treatment. A drop of the suspension was placed on a carbon-coated copper grid, followed by staining with phosphotungstic acid, and the morphology was observed.

Zeta Potential: The Zeta potential of the samples was measured using a Zetasizer Nano ZS90 (Malvern Instruments Ltd, UK) by dispersing 10 mg of sample in 1 mL of water after ultrasonication.

Thermal Stability Analysis: Thermogravimetric analysis (TGA) and glass transition temperature (T_g_) of hydrogel freeze-dried powders were measured by a Thermogravimetric Analysis-Differential Scanning Calorimetry (TGA-DSC) (STA 449F3, NETZSCH, Germany), and the analyses were performed at a flow rate of 50 mL/min under N_2_ atmosphere. A sample (4–8 mg) was placed in an alumina crucible after being equilibrated, and the heating rate was set to 10 ℃/min with a temperature range of 25–580 ℃.

1. **Annual revenue, cost, profit and CO_2_ emission of different process-regulated cases**

First, evaluate the impacts of multi-product production on economic and environmental benefits based on mechanical fractionation, and establish four cases: non-fractional ethanol-only production (without fractionation), co-production of CNC and ethanol, co-production of ethanol and epoxy resin, and co-production of ethanol, CNC, and epoxy resins. Specific process flows and parameters are provided in Table S7.

Based on the process flow constructed in SuperPro Designer, the annual cost, revenue, profit and CO_2_ emission were calculated. The modeling process adhered to the following assumptions: corn stover underwent homogenization following mechanical fractionation; all unit operations were adiabatic (no heat exchange with the environment) and were non-interacting with each other. The annual operating time was 330 days, resulting in a total of 7920 hours. The transportation radius was set to 50 km. The costs associated with utilities, raw materials, transportation, and equipment are provided in Table S8. The price of products is referred to the notes in Table S9.

The total cost, obtained from the process simulation results, along with the total revenue and total profit, are presented in Table S9. CO₂ emissions were calculated using the integrated ecoinvent database within the software.

*Total profit = Total revenue – Total cost*

$$Profit per CO_{2}= \frac{Total profit (CNY/yr)}{CO_{2} emission (kg/yr)}$$

Second, the impacts of different process regulations strategies on the cost and revenue of the multi-product coproduction system were compared. Specifically, the two-stage enzymatic hydrolysis (9 h + 15 h) combined with ultrasonication was replaced by a 24 h enzymatic hydrolysis + ultrasonication system. Furthermore, the steam explosion process and the enzymatic hydrolysis process were reverted to the state without process control (without addition of methanol and CQD), thereby establishing an uncontrolled multi-product coproduction line as a reference. Using this as a baseline, the effects of individually regulating the CNC production process and the lignin-based epoxy resin production process, as well as simultaneously regulating both processes, on product cost and yield were compared. Detailed measures are provided in the notes to Table S10. The process parameters, costs, and product prices remained consistent with those mentioned earlier. The impacts of individual regulation and synergistic regulation on product output and total revenue were calculated. Taking the production change and revenue change of CNC as an example, the calculation methods for other products followed a similar approach.

$$CNC Production change= \frac{CNC production after regulation-CNC production before regulation}{Total production after reguation-Total production before regulation}$$

$$Total production=Ethanol+CNC+Liquid resin+Solid resin production$$

$$CNC revenue change= \frac{CNC revenue after regulation-CNC revenue before regulation}{Total revenue after reguation-Total revenue before regulation}\times Revenue growth rate$$

$$Total revenue=Ethanol+CNC+Liquid resin+Solid resin production$$

$$Revenue growth rate= \frac{Total revenue after reguation-Total revenue before regulation}{Total revenue before regulation}$$

1. **Statistical analysis**

Experimental data were analyzed in triplicate and expressed as mean values ± standard deviation. Statistical analysis was performed to determine significant differences in selected data using Origin 8.0 (OriginLab, USA).

**Supplementary Figures**


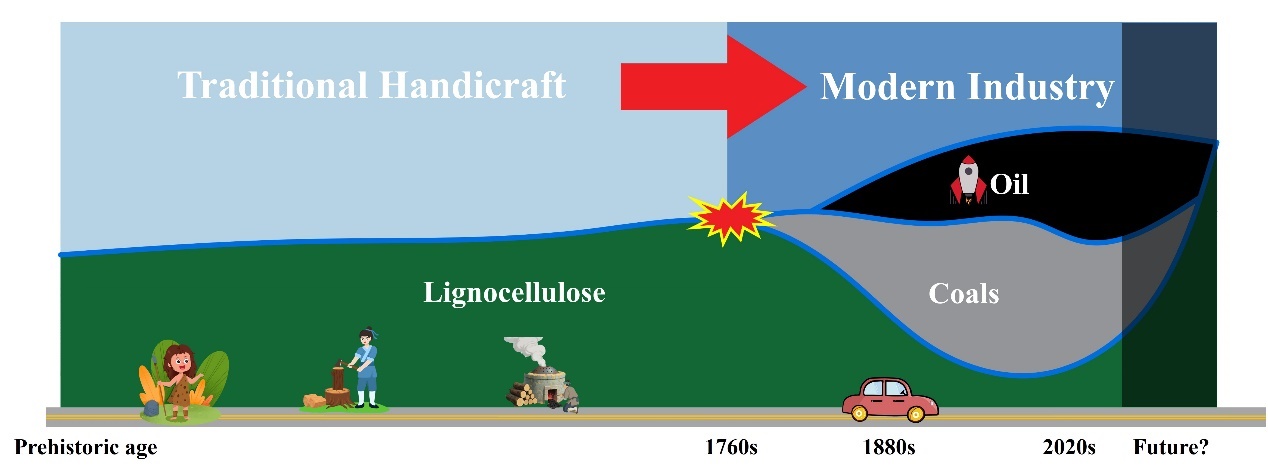


**Figure S1.** The role of lignocellulose and petroleum in driving industrial transformation throughout human history*.*


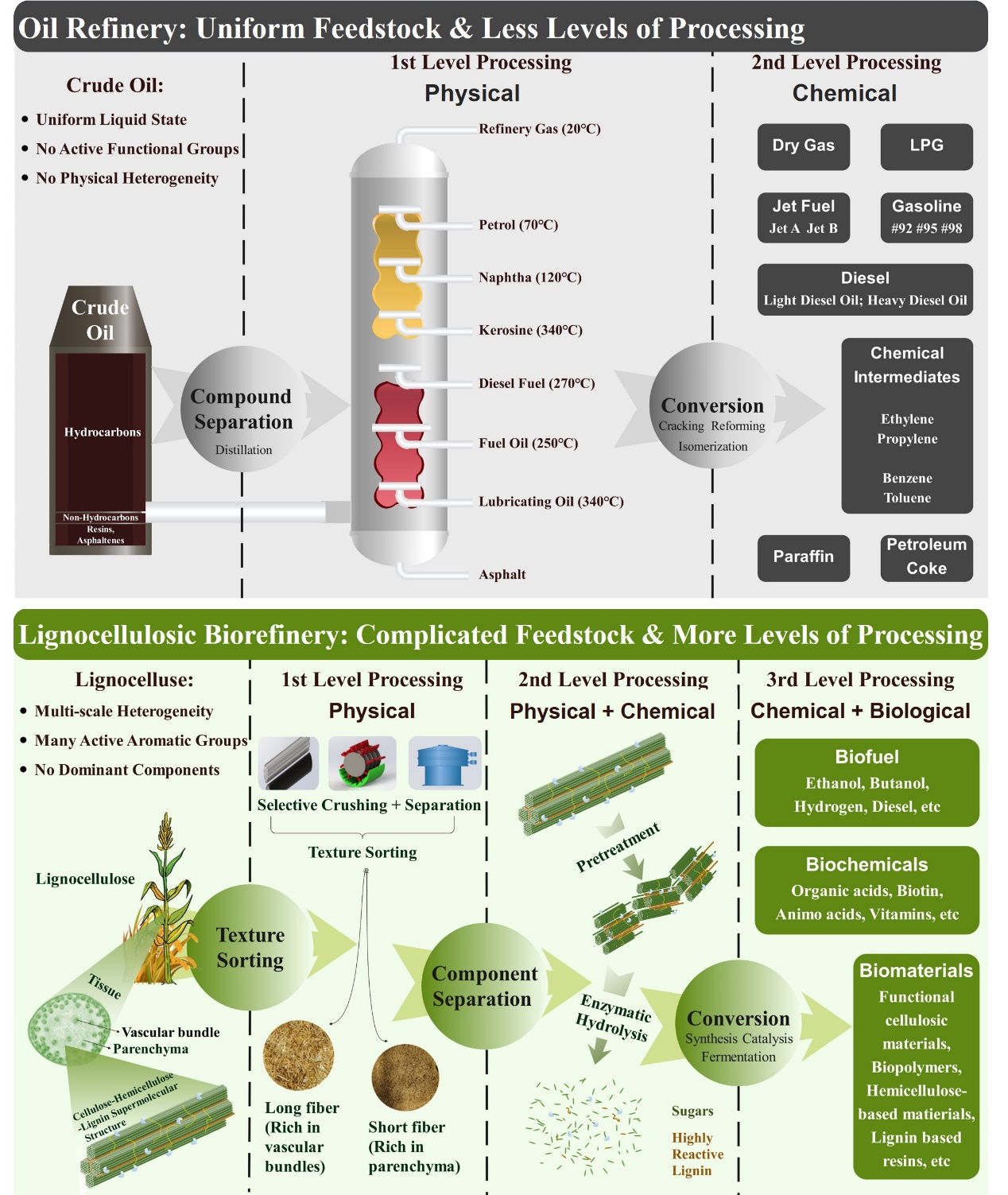


**Figure S2.** The comparison between oil refinery and lignocellulose biorefinery.


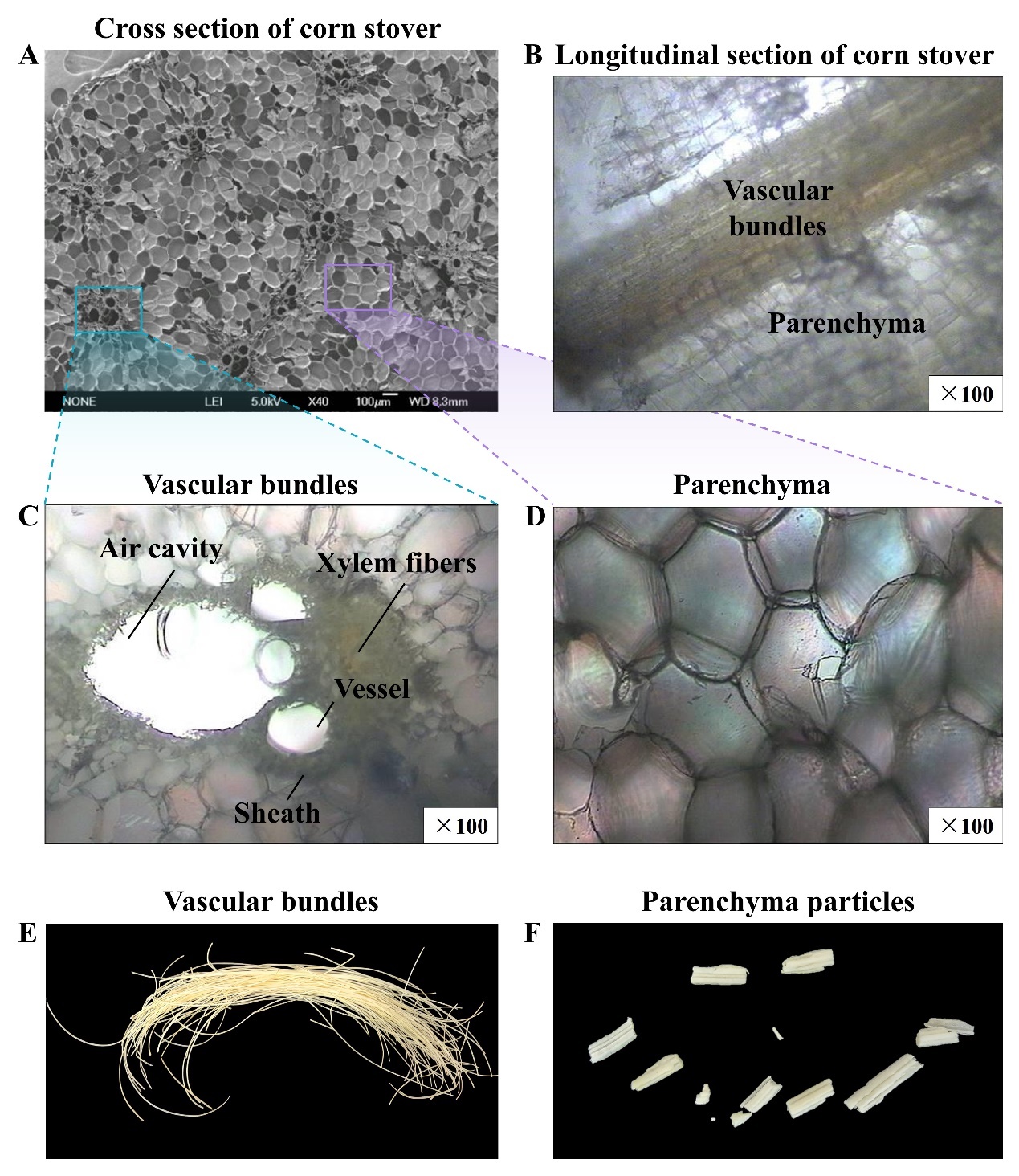


**Figure S3.** The anatomical structure of vascular bundles and parenchyma of corn stover. A) The cross section of corn stover. B) The longitudinal section of corn stover. C) The structural composition of vascular bundle of corn stover. D) The cross section of parenchyma of corn stover. E) Vascular bundles from corn stover pith. F) Parenchyma particles from corn stover pith.

*
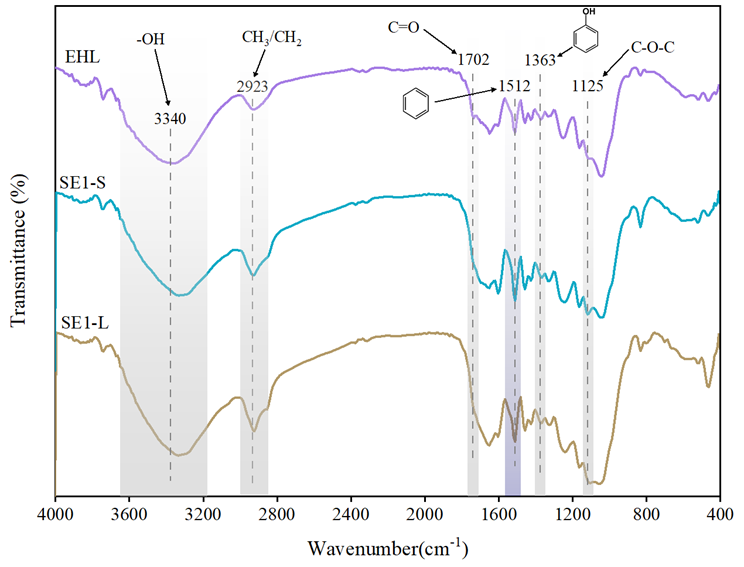
*

**Figure S4.** FTIR spectra of EHL from steam-exploded (1 min) corn stover (SE1) and steam-exploded (1 min) corn stover long fibers fraction (SE1-L) and short fibers fraction (SE1-S). EHL, Enzyme-hydrolyzed lignin;

SE1-S, Enzyme-hydrolyzed lignin from short fibers of 1-min steam-exploded corn stover;

SE1-L, Enzyme-hydrolyzed lignin from long fibers of 1-min steam-exploded corn stover.


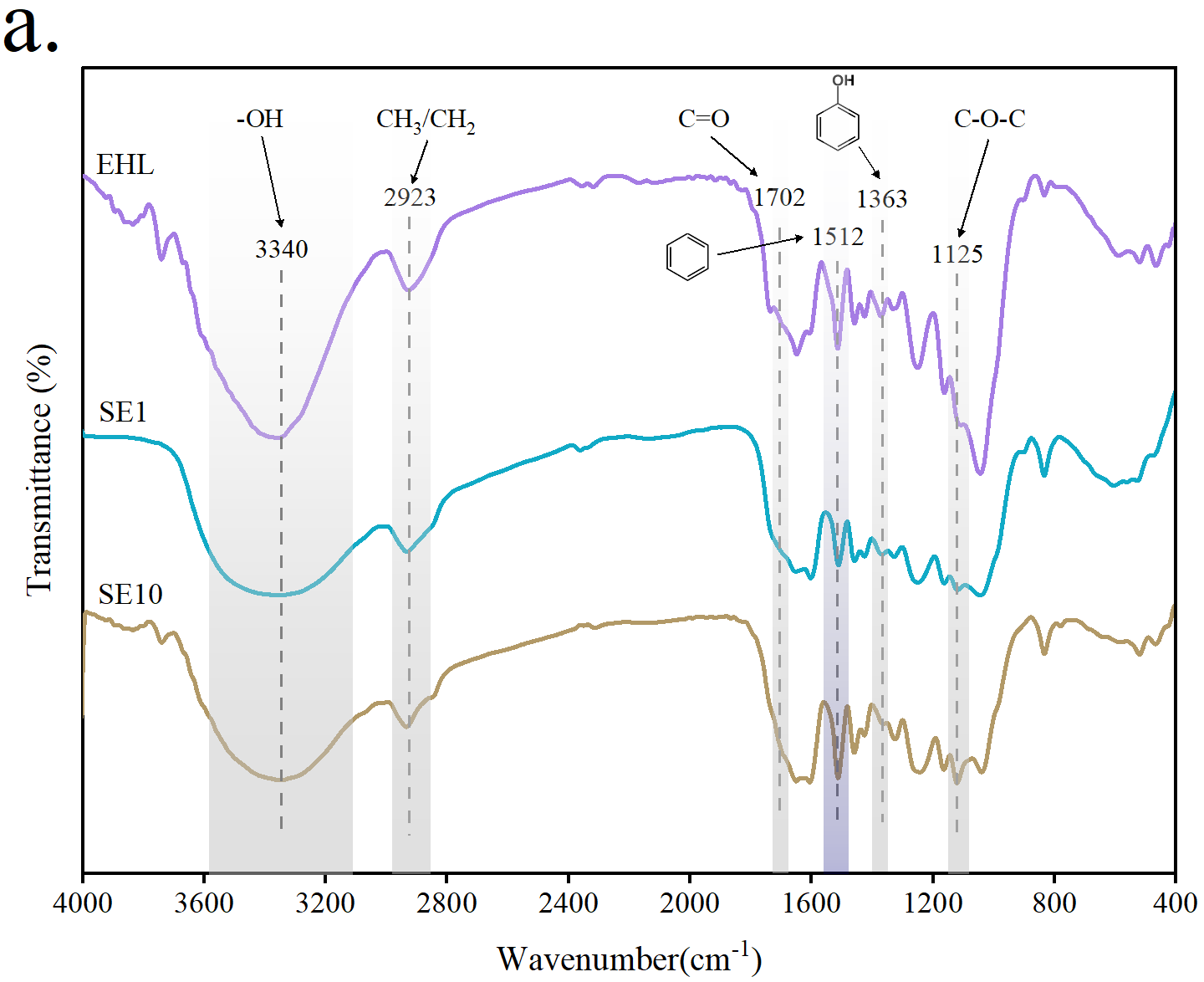


**Figure S5.** Differences in chemical structures characterized by FTIR spectra of EHL and EHL (SE1 and SE10) from corn stover under different steam explosion conditions (1.0 MPa, 1 min and 1.0 MPa, 10 min).

EHL, Enzyme-hydrolyzed lignin;

SE1, Enzyme-hydrolyzed lignin from 1-min steam-exploded corn stover;

SE10, Enzyme-hydrolyzed lignin from 10-min steam-exploded corn stover.


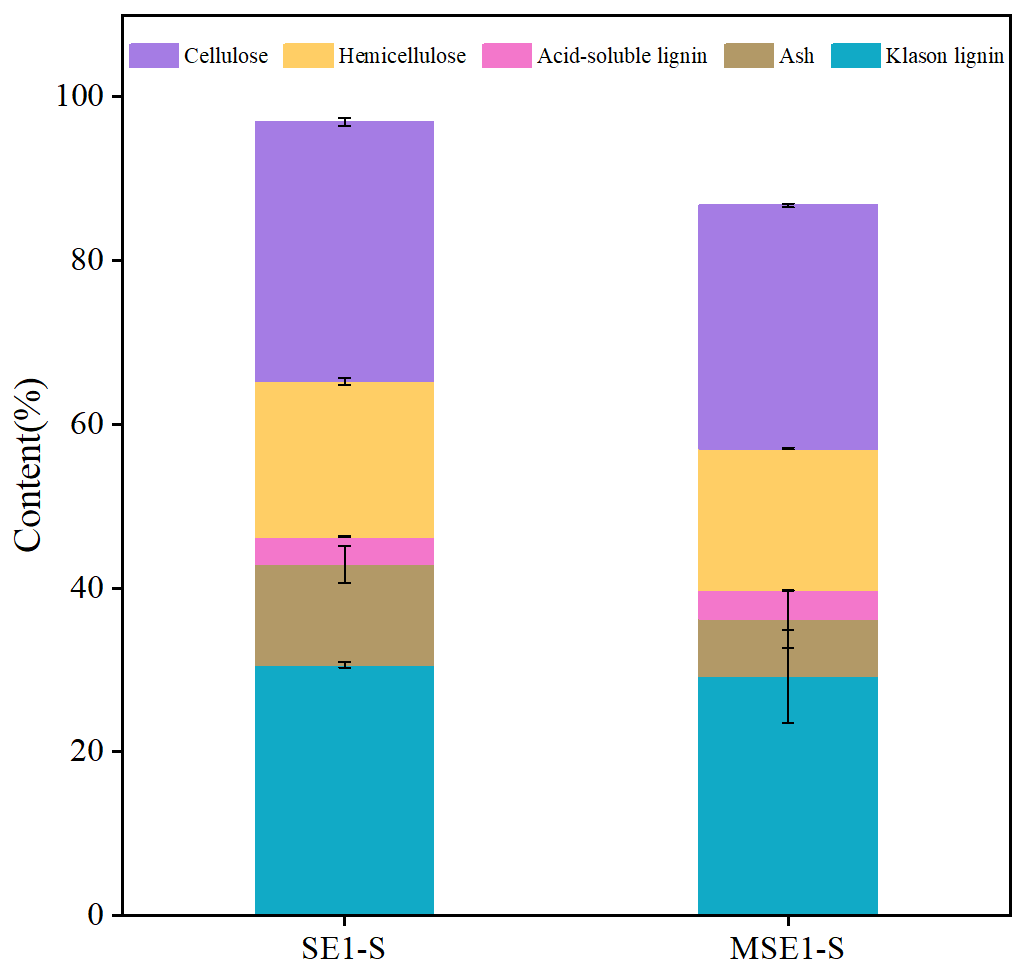


**Figure S6.** Effect of steam explosion (1 min) without/with methanol on the change of corn stover short fibers components.

SE1-S, Enzyme-hydrolyzed lignin from short fibers of 1-min steam-exploded corn stover; MSE1-S, Enzyme-hydrolyzed lignin from short fibers of 1-min methanol-protected steam-exploded corn stover.


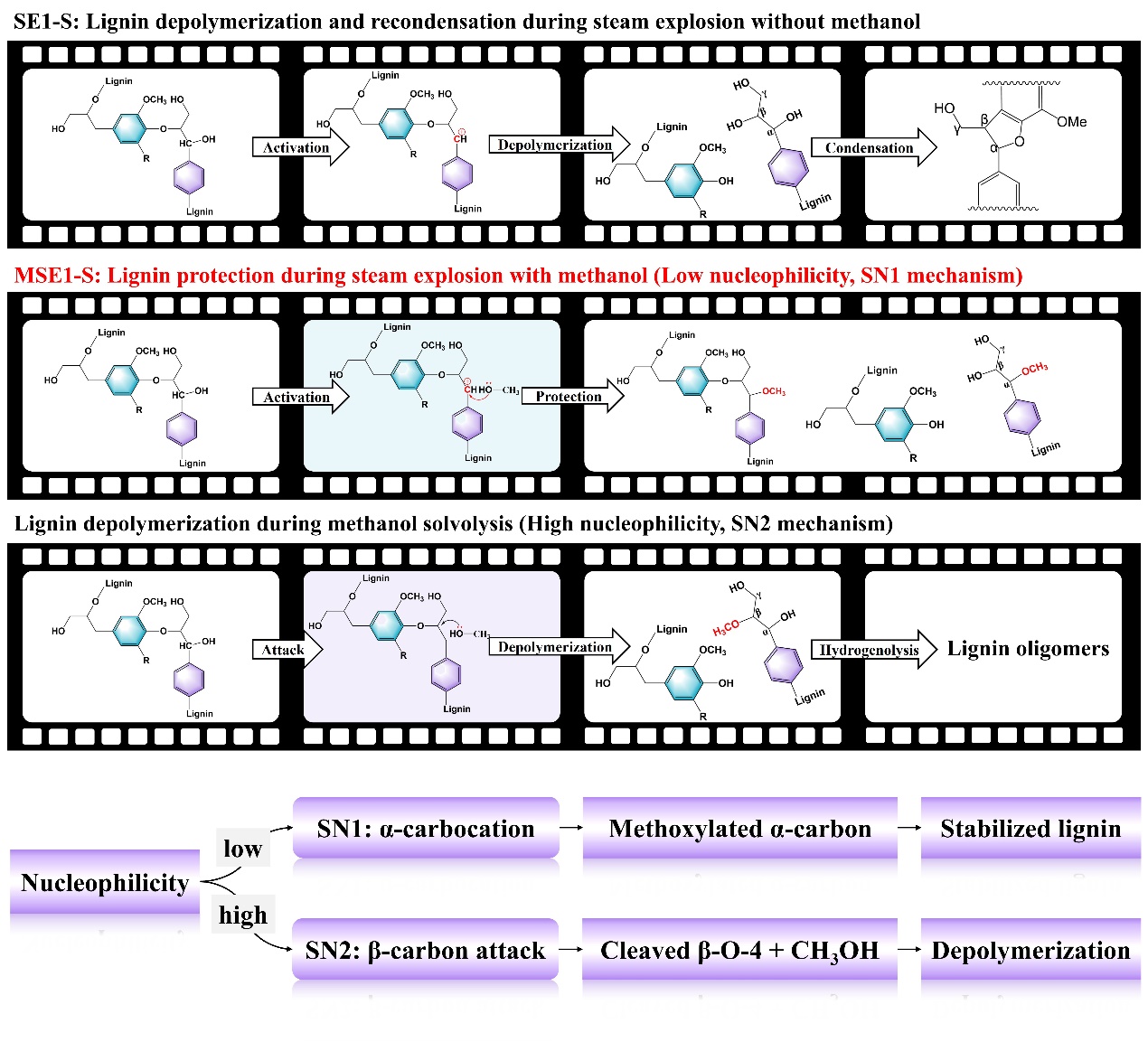


**Figure S7.** The proposed mechanism of steam explosion without and with methanol, and methanol depolymerization (Derived from Figure 2).

In the steam explosion environment without methanol addition, lignin undergoes both depolymerization and recondensation.

With methanol, we deduce that under mildly nucleophilic conditions (as demonstrated in this work), methanol favors methoxylation at the α-carbon through SN1, thereby suppressing β-O-4 cleavage and retarding depolymerization. In contrast, under strong nucleophilic conditions, methanol predominantly cleaves β-O-4 linkages via SN2, promoting lignin depolymerization.


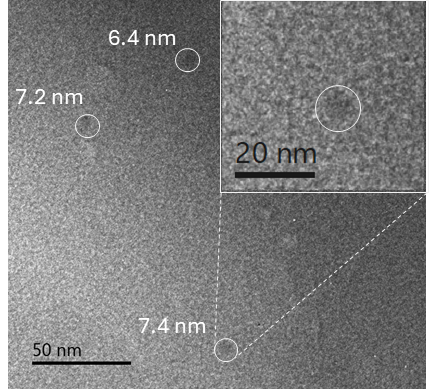


**Figure S8.** TEM images of CQDs prepared by EHL hydrothermally.

EHL, Enzyme-hydrolyzed lignin;

TEM, Transmission electron microscope;

CQDs, Carbon quantum dots.


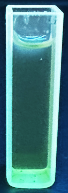

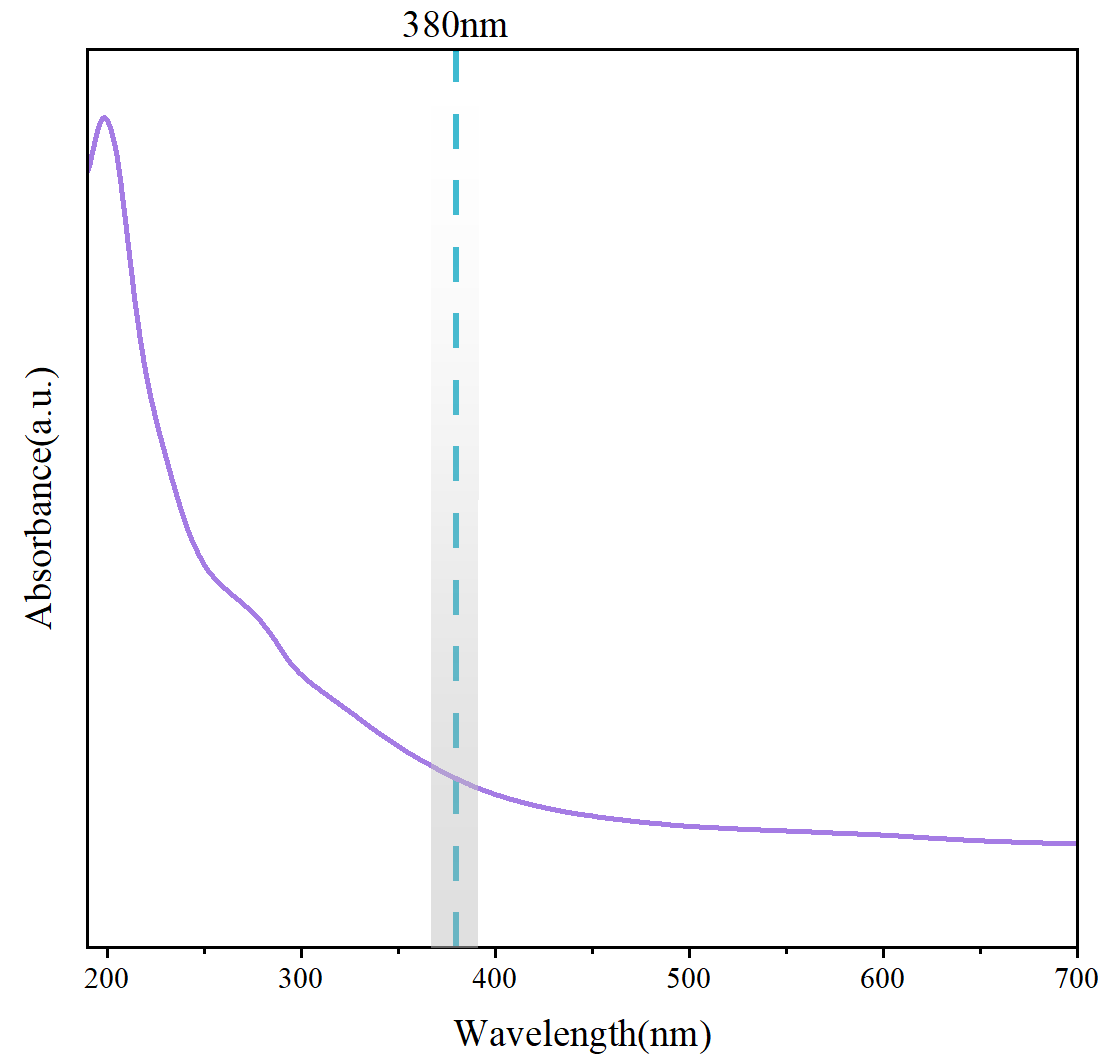


**Figure S9.** CQDs UV-Vis absorption spectrum and excitation of green light.

CQDs, Carbon quantum dots.


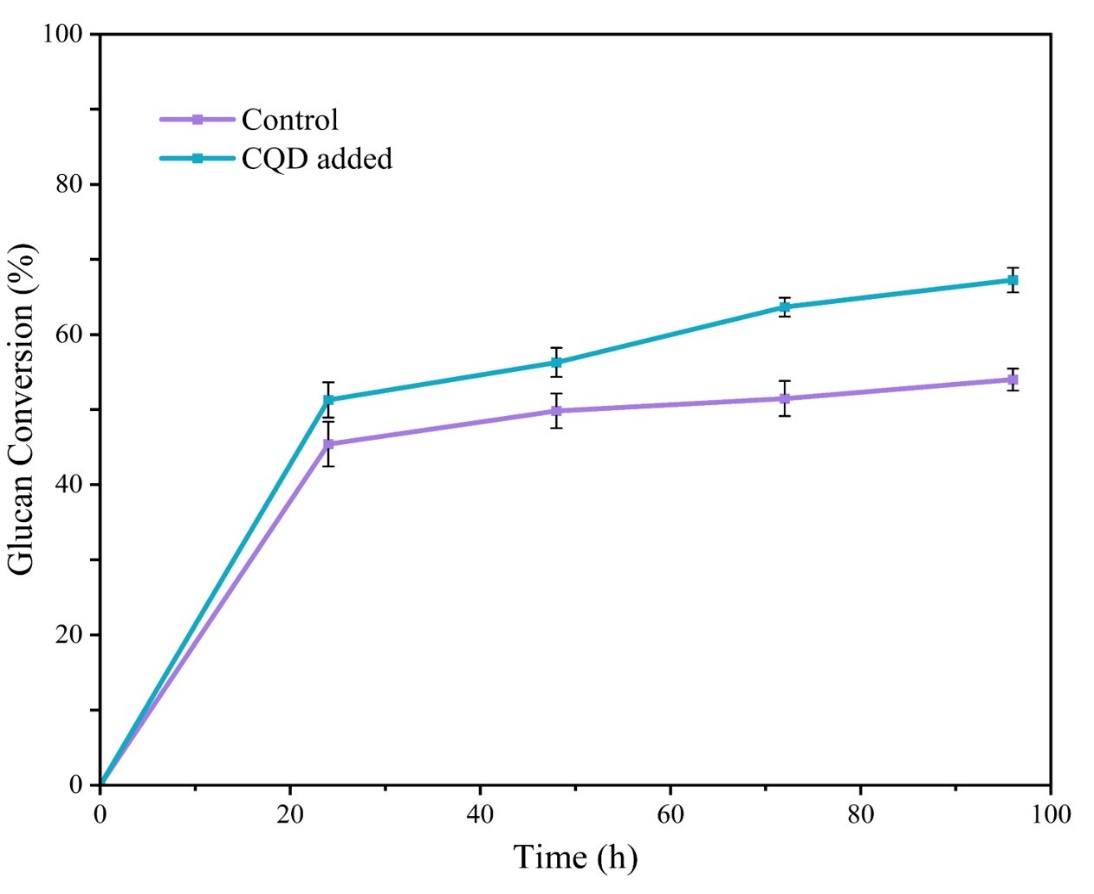


**Figure S10.** Comparison on glucan conversion rate of methanol-protected steam explosion corn stover short fibers before and after CQDs addition.

CQDs, Carbon quantum dots.

| Sample | Mn | Mw | PDI |
| --- | --- | --- | --- |
| MSE1-S | 12082.00 | 22852.43 | 1.89 |
| MSE1-S-CQD | 6087.35 | 19016.12 | 3.12 |

**Figure S11.** Molecular weight distribution and detector responses of EHL from methanol-protected steam exploded corn stover short fibers before and after CQD addition.

EHL, Enzyme-hydrolyzed lignin;

MSE1-S, Enzyme-hydrolyzed lignin from short fibers of 1-min methanol-protected steam-exploded corn stover;

MSE1-S-CQD, Carbon quantum dot-enhanced enzyme-hydrolyzed lignin from short fibers of 1-min methanol-protected steam-exploded corn stover;

Mn, Number-average molecular weight;

Mw, Weight-average molecular weight;

PDI, Polydispersity index; PDI=Mw/Mn.


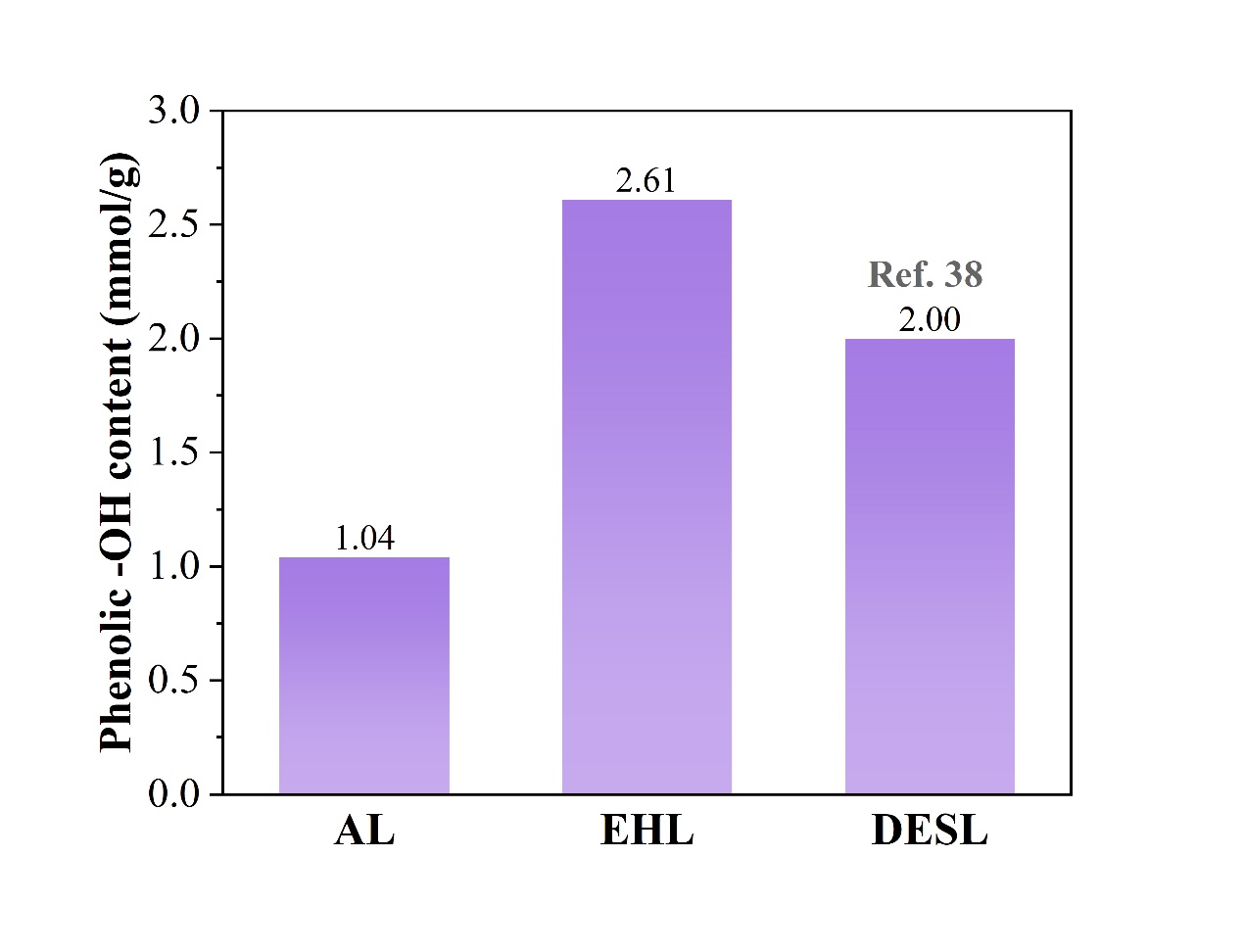


**Figure S12.** The total phenolic hydroxyl group content of AL and EHL.

AL, Alkali lignin;

EHL, Enzyme-hydrolyzed lignin;

DESL, Deep eutectic solvent extracted lignin.


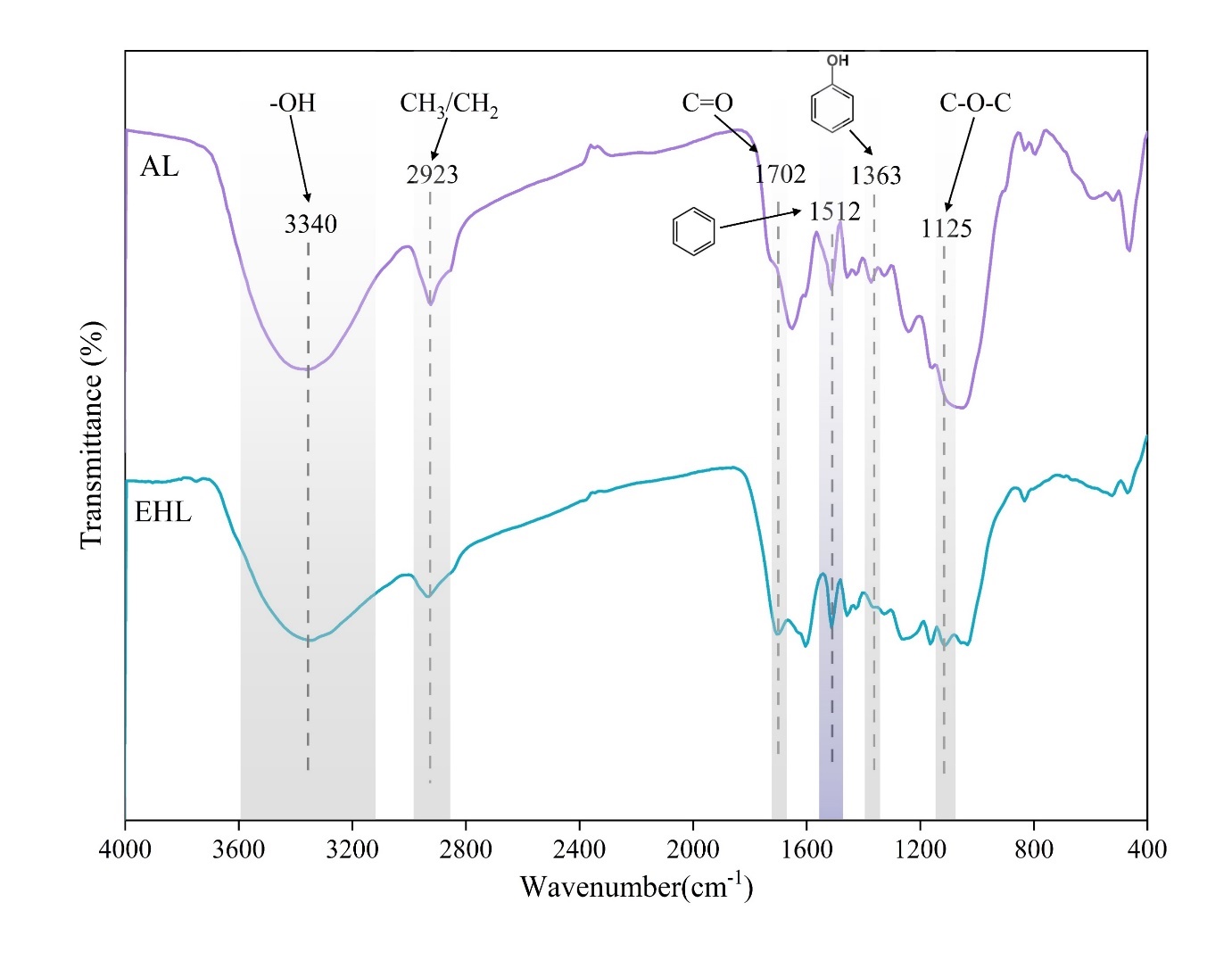


**Figure S13.** FTIR spectra characterize the differences in chemical structure between AL and EHL.

AL, Alkali lignin;

EHL, Enzyme-hydrolyzed lignin.


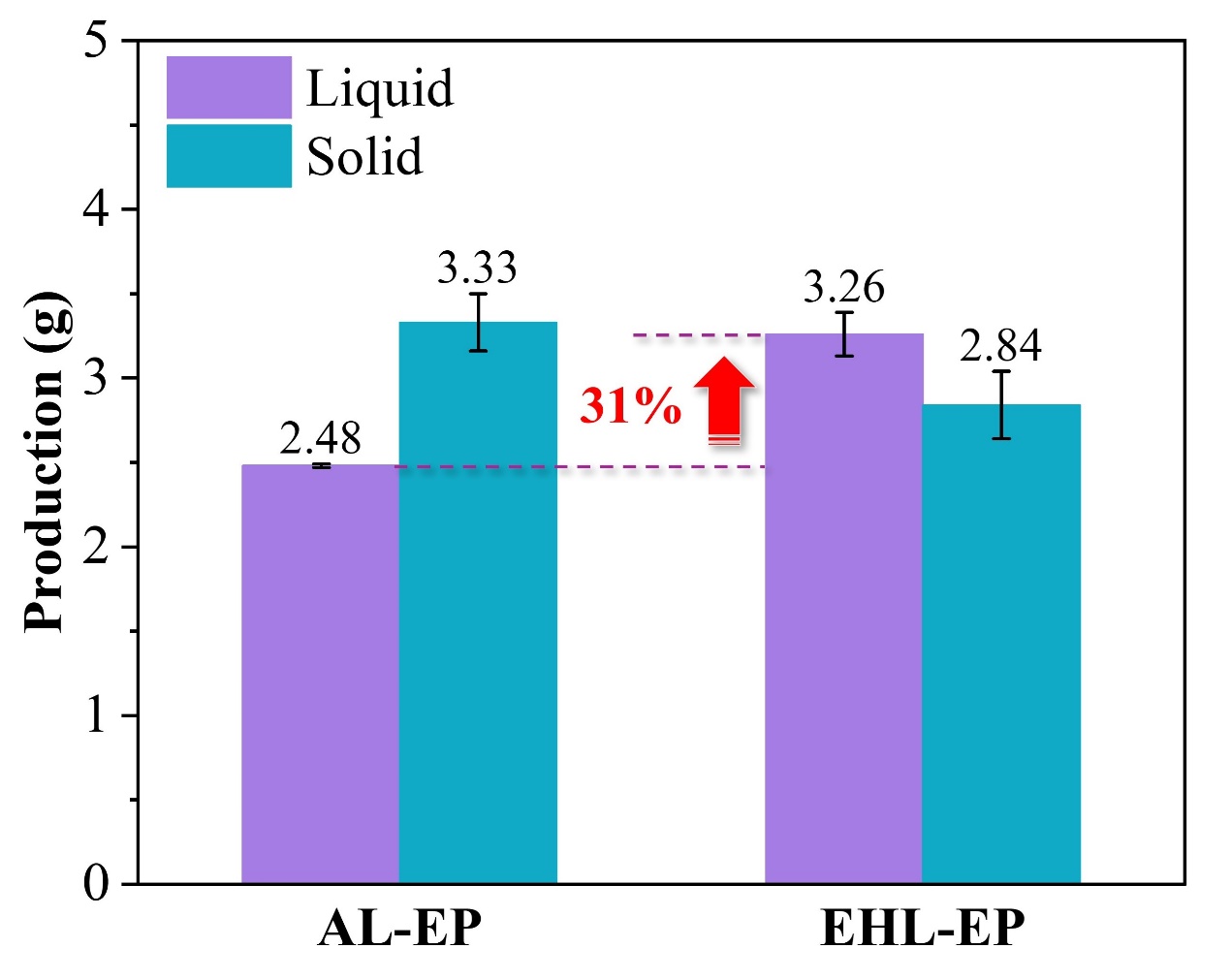


**Figure S14.** Yield of alkali lignin-based epoxy resin and enzymatically hydrolyzed lignin-based epoxy resin.

EHL-EP, Enzyme-hydrolyzed lignin epoxy precursor;

AL-EP, Alkali lignin epoxy precursor.


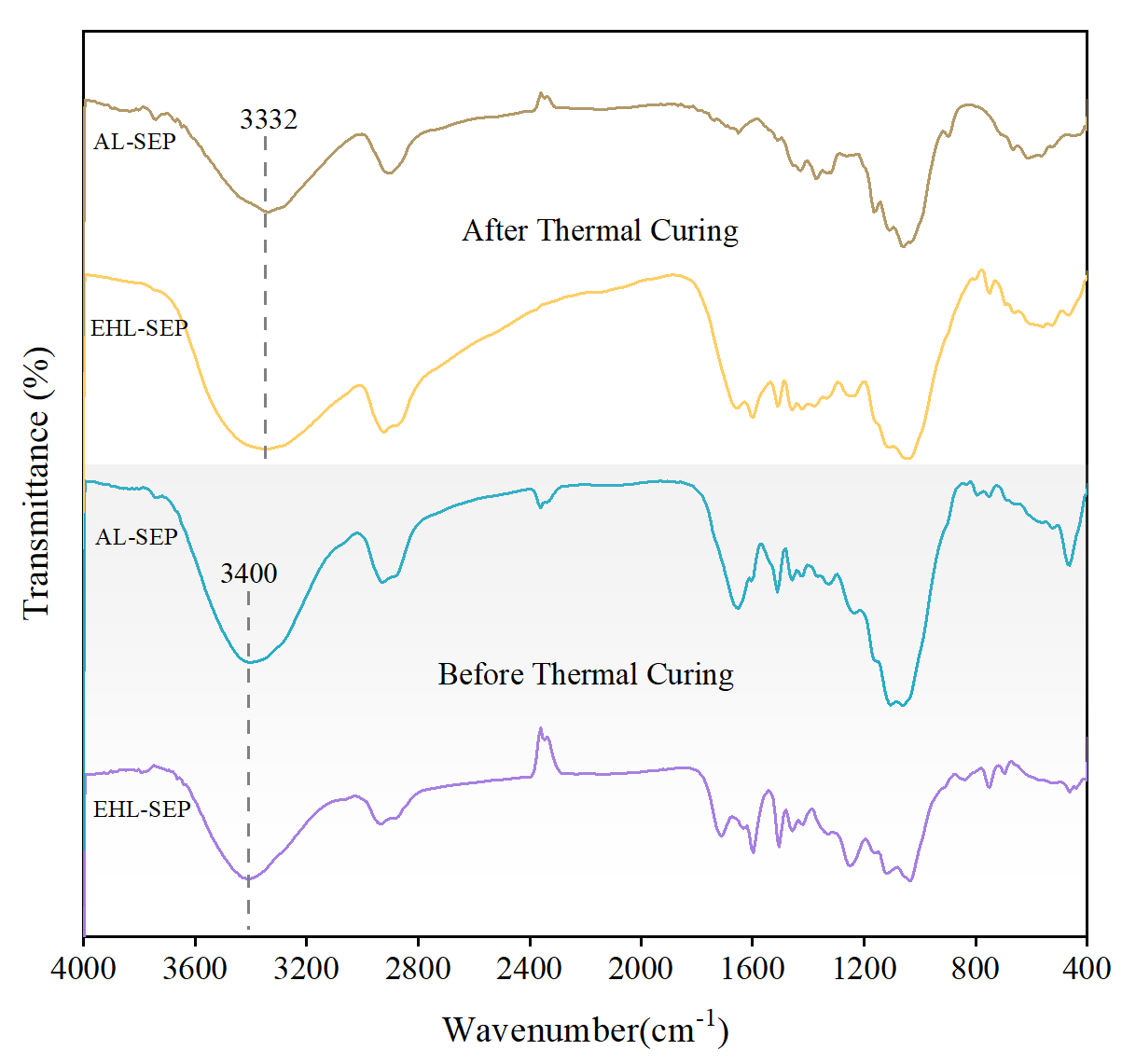


**Figure S15.** FTIR spectra characterizing chemical structural differences of alkali lignin solid epoxy resin and enzymatic lignin solid epoxy resin before and after thermal curing.

AL-SEP, Alkali lignin based solid epoxy resin;

EHL-SEP, Enzyme-hydrolyzed lignin based solid epoxy resin.


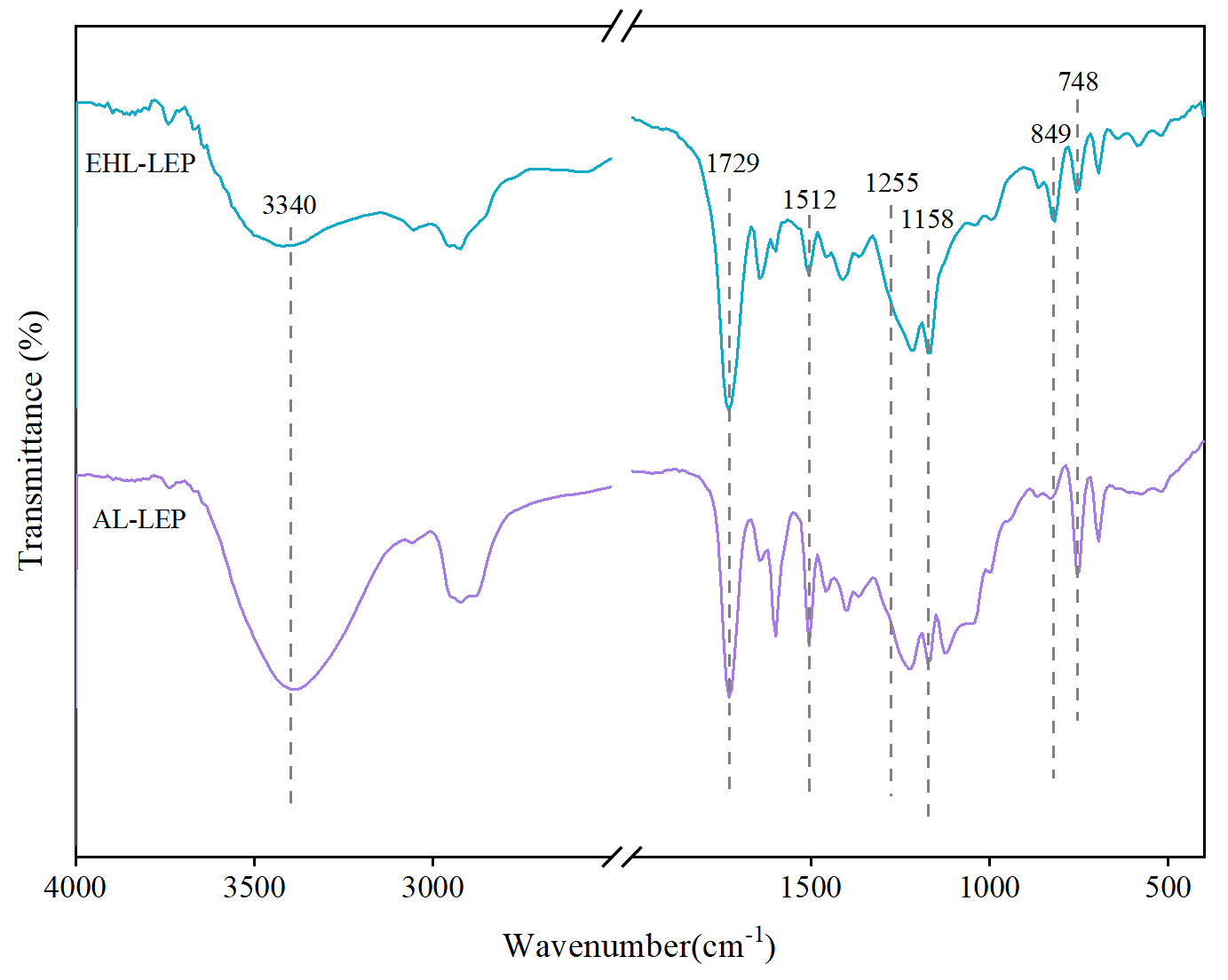


**Figure S16.** FTIR spectra characterizing the chemical structure of maleic anhydride-cured alkaline lignin and Enzyme-hydrolyzed lignin liquid epoxy resins.

AL-LEP, Alkali lignin based liquid epoxy resin;

EHL-LEP, Enzyme-hydrolyzed lignin based liquid epoxy resin.


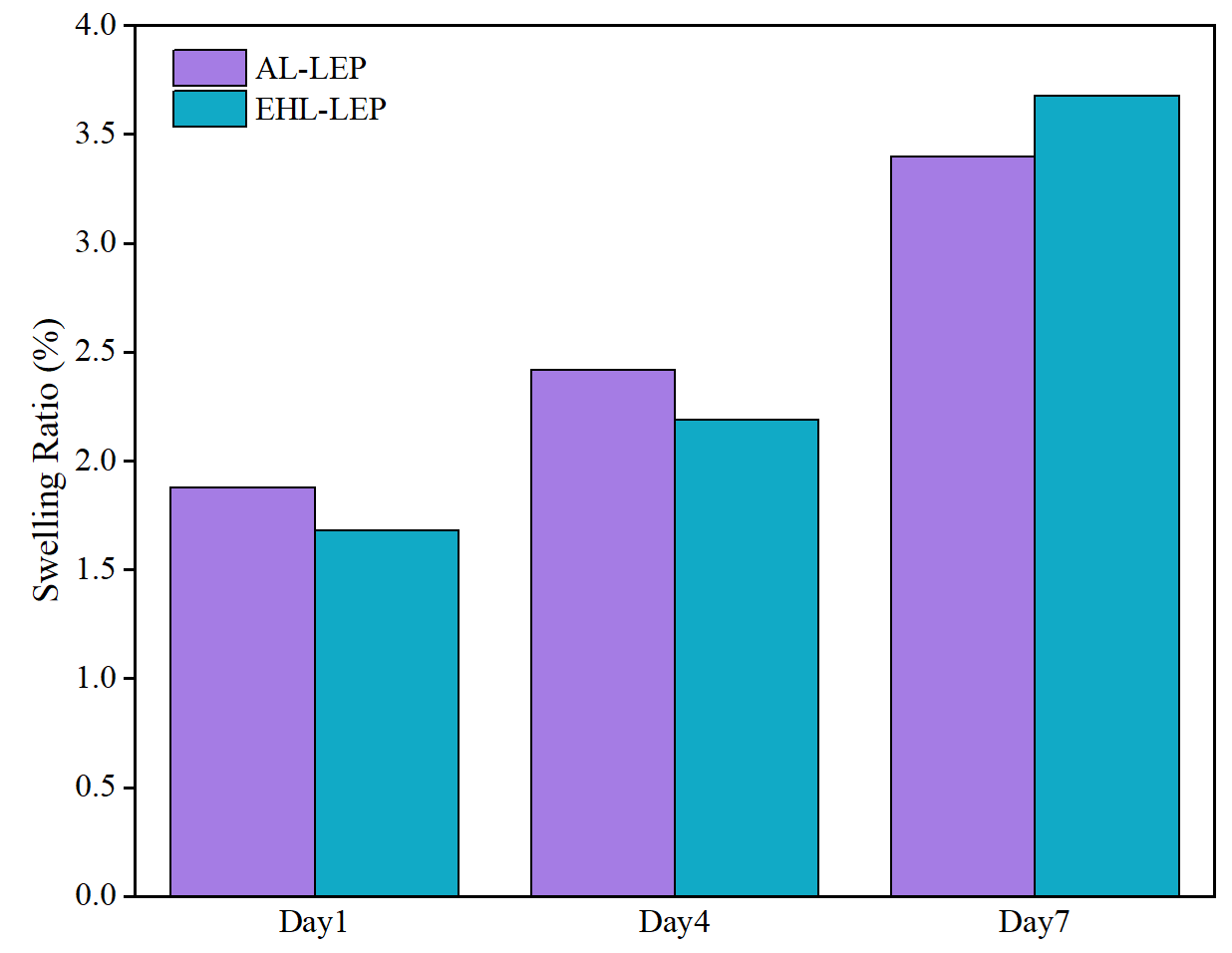


**Figure S17.** Swelling ratio of liquid epoxy resins in water between alkaline lignin and EHL liquid epoxy resin.

AL-LEP, Alkali lignin based liquid epoxy resin;

EHL-LEP, Enzyme-hydrolyzed lignin based liquid epoxy resin.


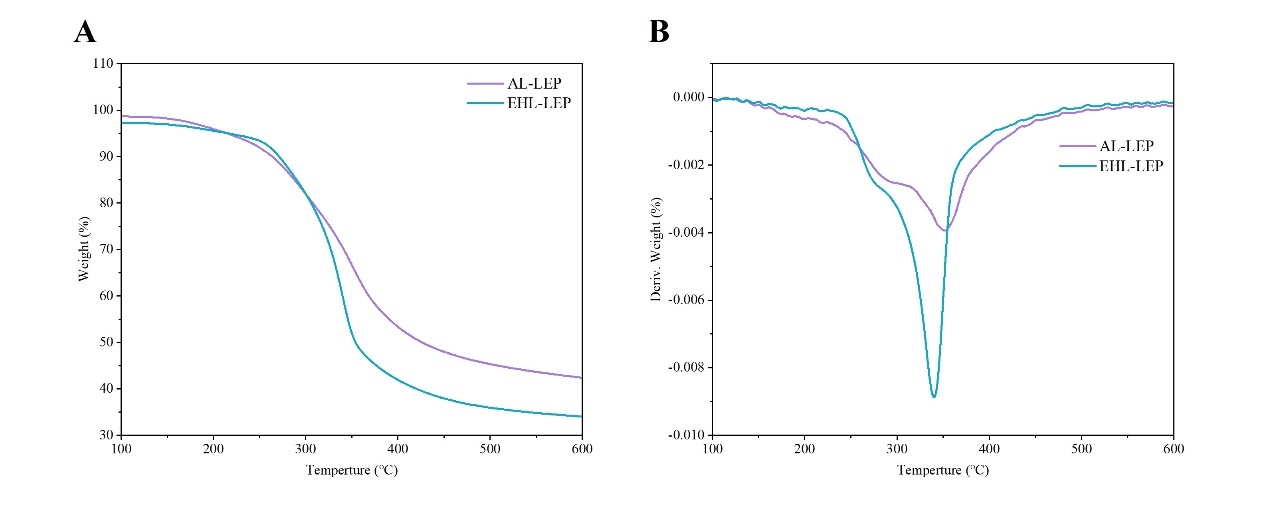


**Figure S18.** TG (A) and DTG (B) curves of liquid epoxy resins characterize the differences in thermal stability between alkaline lignin and EHL liquid epoxy resins.

AL-LEP, Alkali lignin based liquid epoxy resin;

EHL-LEP, Enzyme-hydrolyzed lignin based liquid epoxy resin.

*
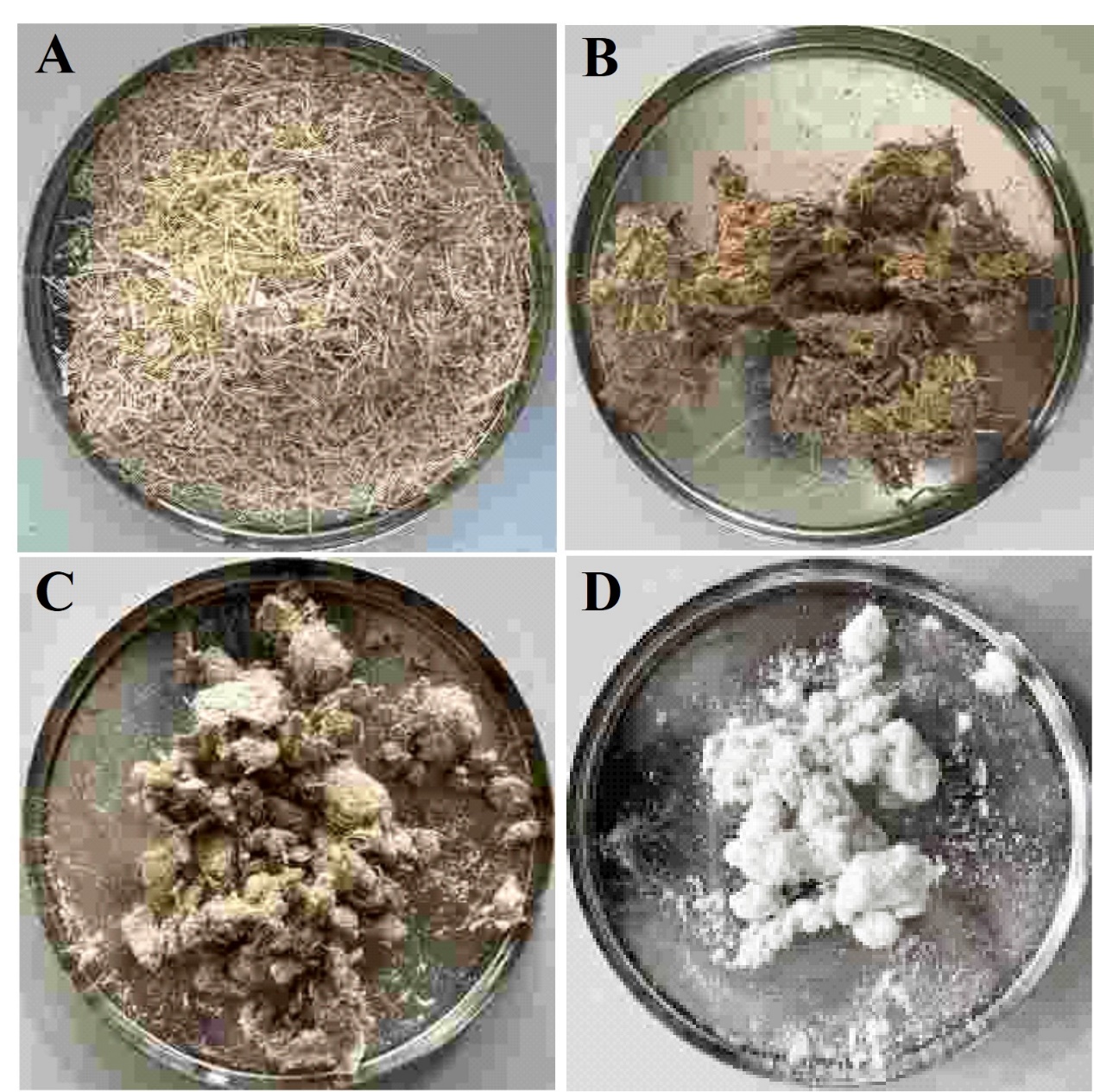
*

**Figure S19.** Photos of A) corn stover long fibers, B) after steam explosion, C) lignin removal with DES, and D) H_2_O_2_ bleaching treatments.

*
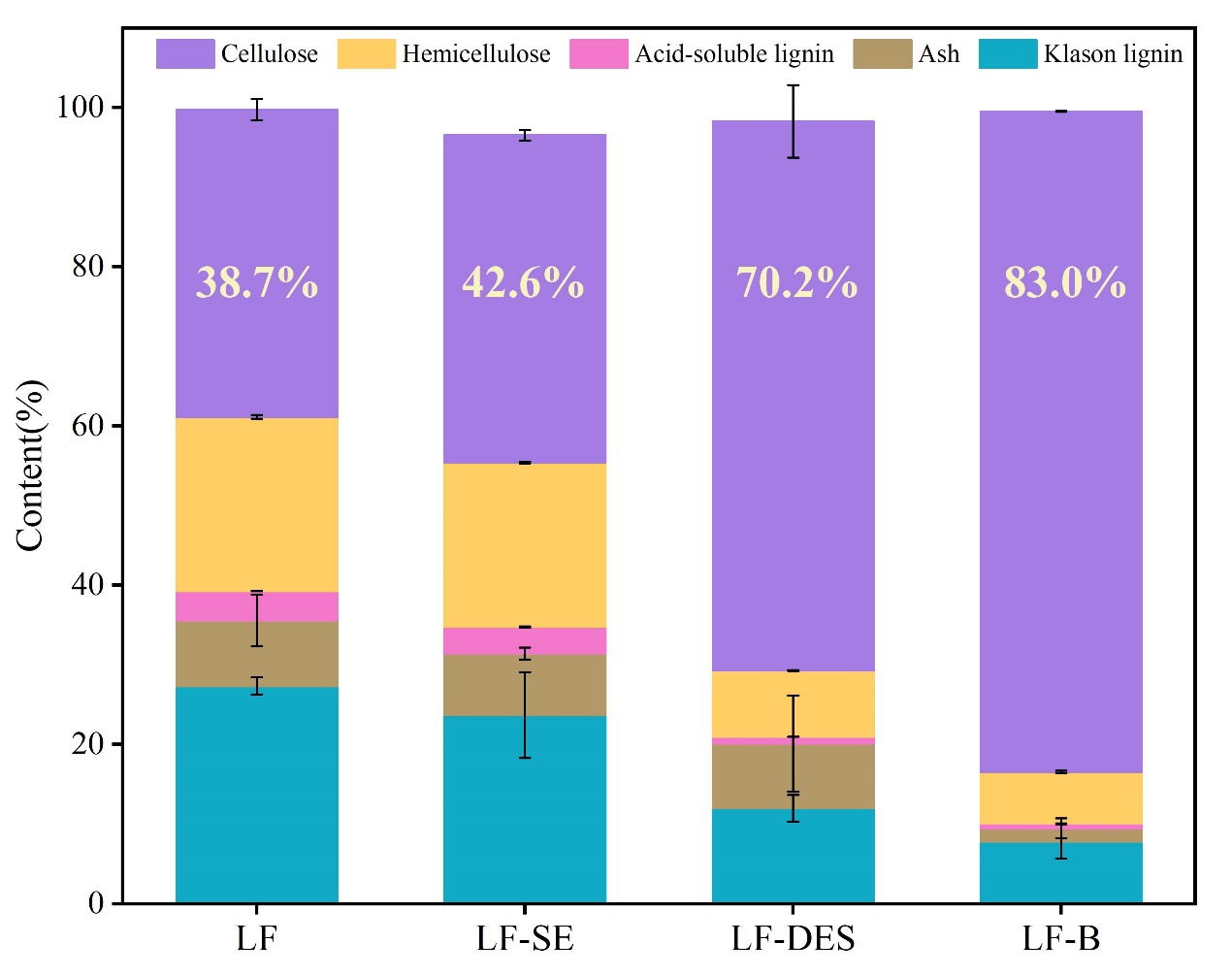
*

**Figure S20.** Component changes of corn stover long fibers (LF) after steam explosion (LF-SE), lignin removal with DES (LF-DES), and H_2_O_2_ bleaching (LF-B) treatments.

LF: Long fibers;

LF-SE: Long fibers after steam explosion;

LF-DES: Long fibers after DES treatment;

LF-B: Long fibers after H_2_O_2_ bleaching.


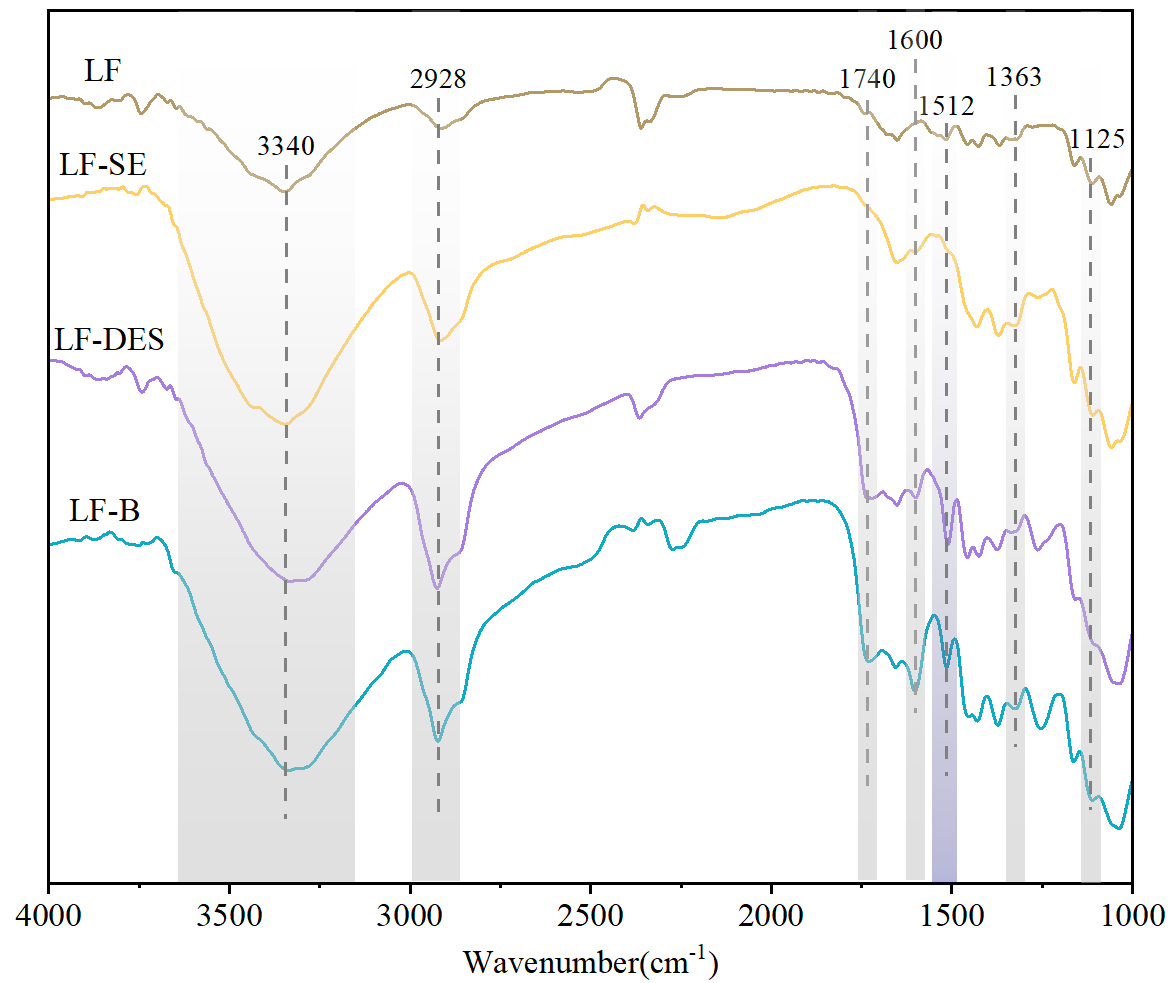


**Figure S21.** FTIR spectra of corn stover long fibers (LF) after steam explosion (LF-SE), lignin removal with DES (LF-DES), and H_2_O_2_ bleaching (LF-B) treatments.

LF: Long fibers;

LF-SE: Long fibers after steam explosion;

LF-DES: Long fibers after DES treatment;

LF-B: Long fibers after H_2_O_2_ bleaching.


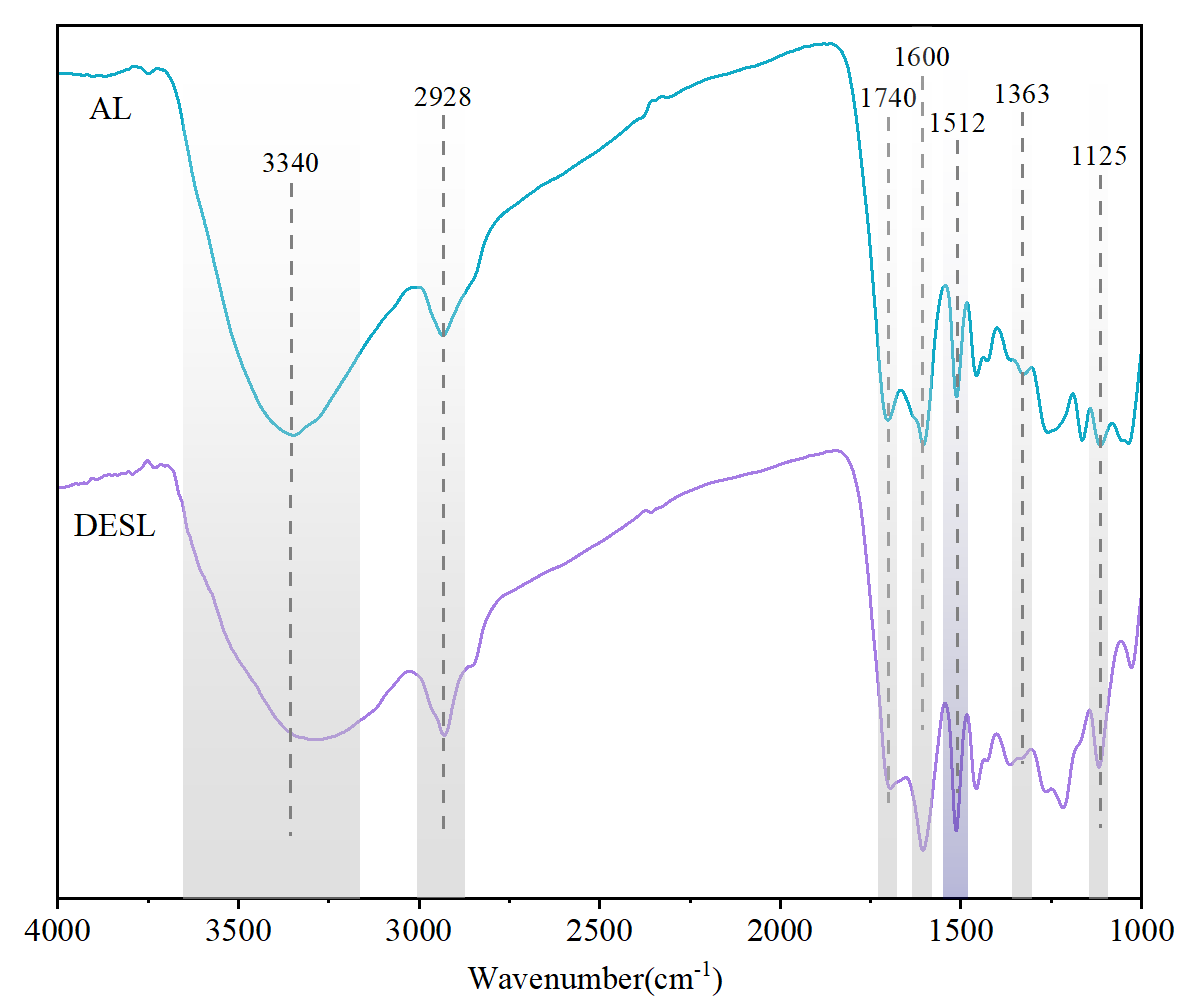


**Figure S22.** FTIR spectra of alkali lignin (AL) and DES extracted lignin (DESL) from steam-exploded corn stover long fibers.


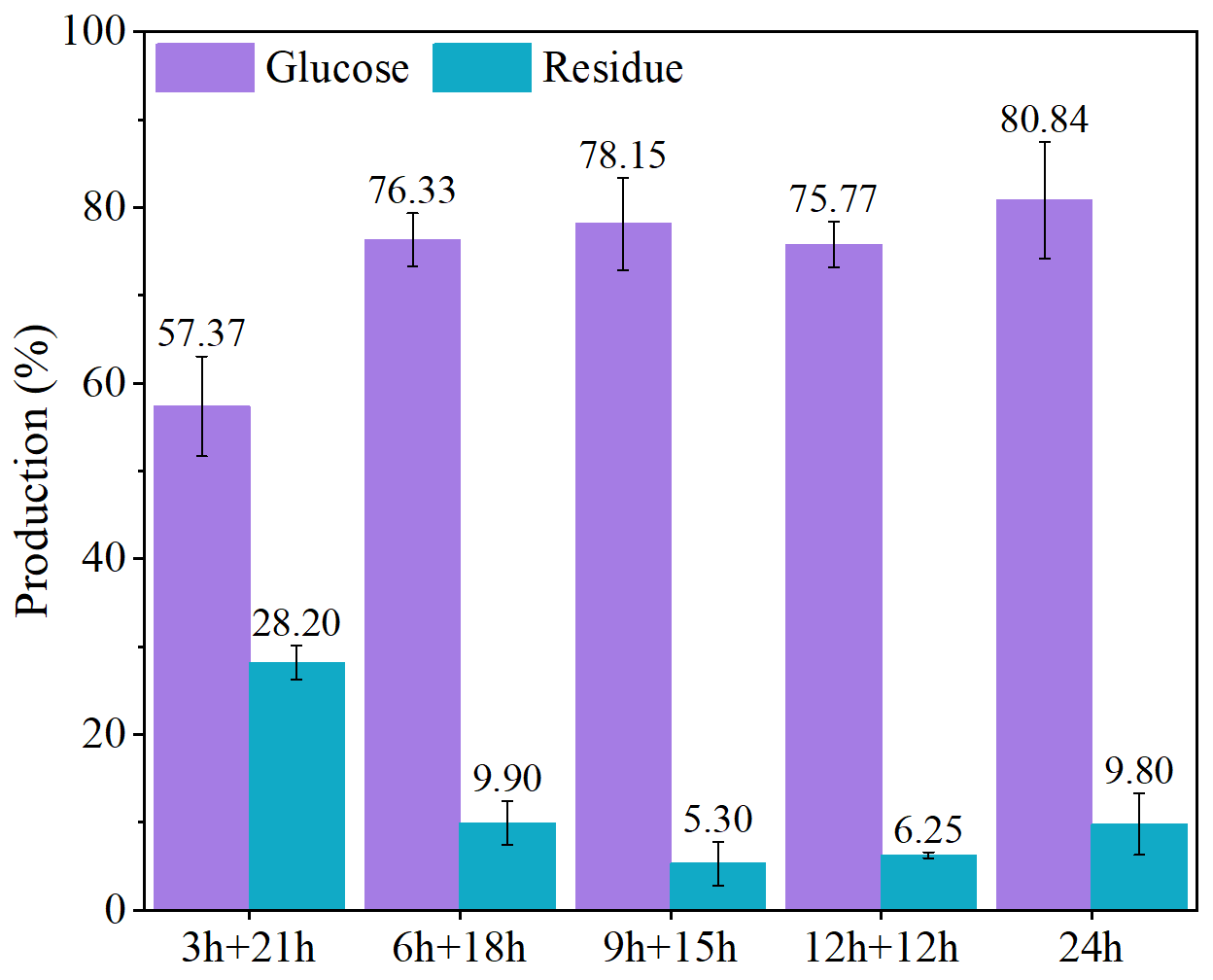


**Figure S23.** Glucose and residue production under different two-stage enzymatic hydrolysis strategies.


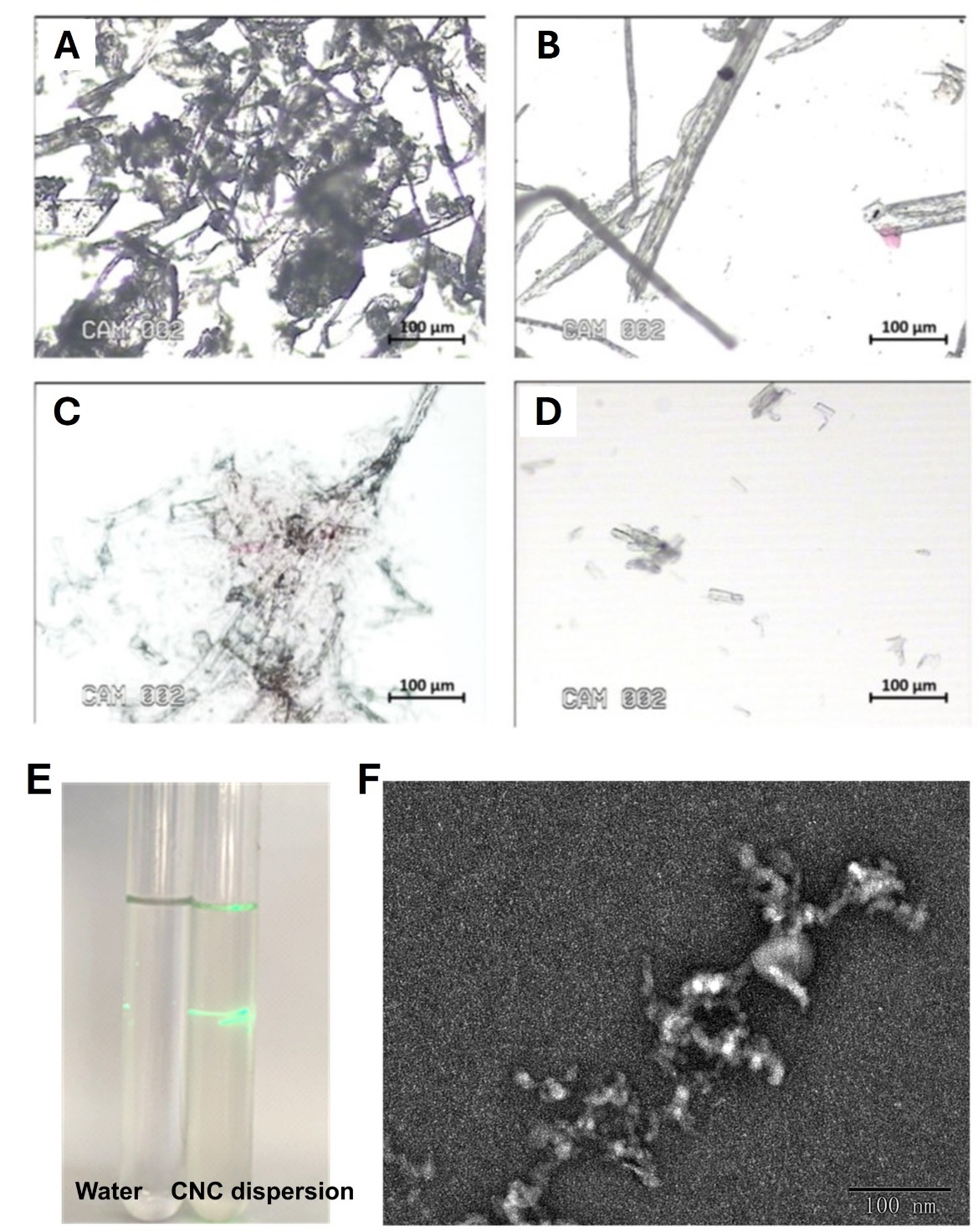


**Figure** **S24.** Optical electron micrographs of each stage in two-stage enzymatic hydrolysis. A) Morphology of bleached steam-exploded corn stover long fibers. B) Morphology and cellulase distribution (red circle) of the substrate after the first stage of the 9 h enzymatic hydrolysis. C) Morphology and cellulase distribution (red circle) of enzyme-hydrolyzed substrate after sonication for 30 min. D) Morphology of the residue from the second stage of 15 h enzymatic hydrolysis of the substrate treated with ultrasound for 30 min. E)Tyndall effect of CNC dispersion, F) TEM image of CNC.


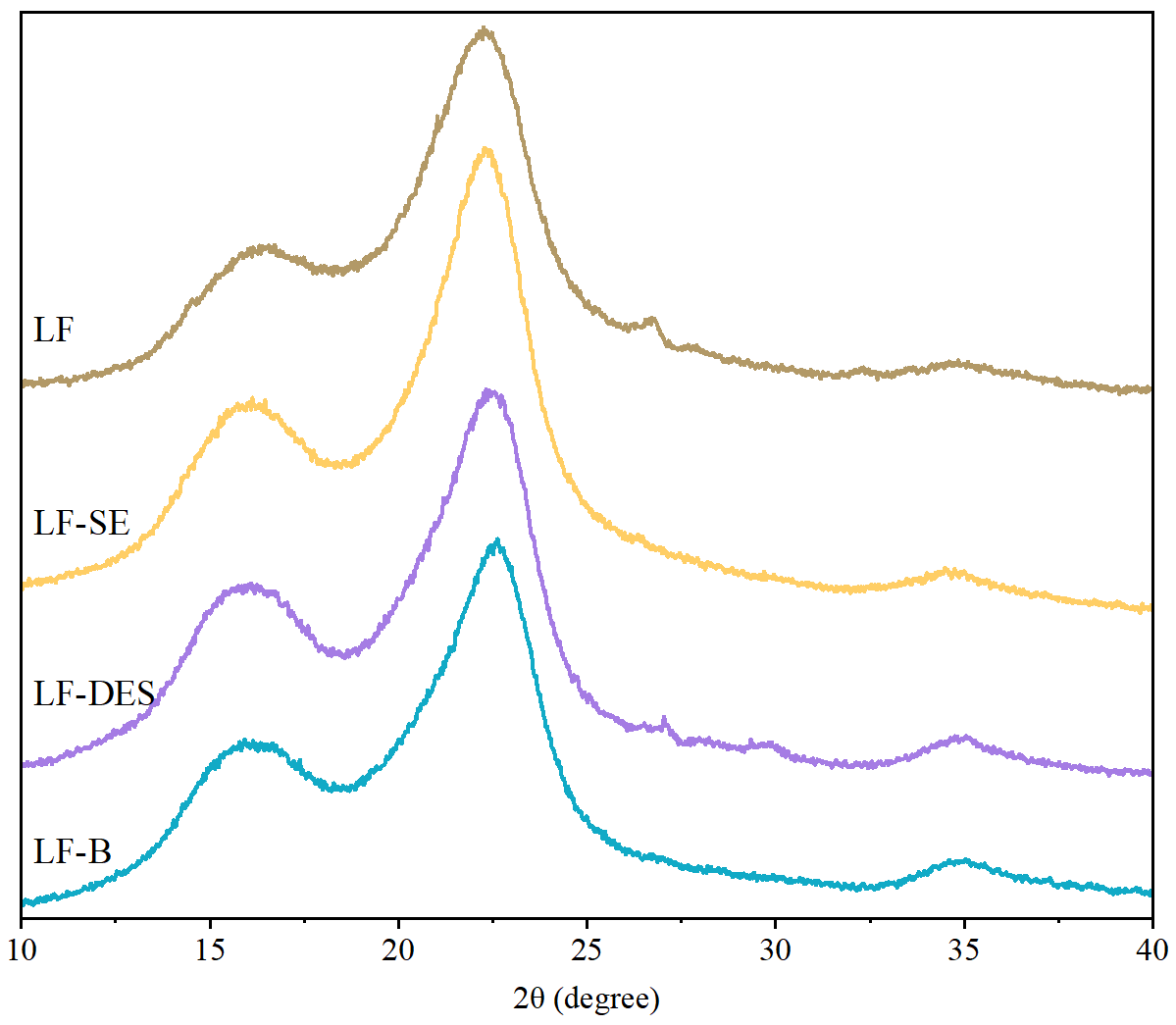


**Figure S25.** XRD spectra of corn stover long fibers (LF) after steam explosion (LF-SE), lignin removal with DES (LF-DES), and H_2_O_2_ bleaching (LF-B) treatments.

LF, Long fibers;

LF-SE, Long fibers after steam explosion;

LF-DES, Long fibers after DES treatment;

LF-B, Long fibers after H_2_O_2_ bleaching.

*
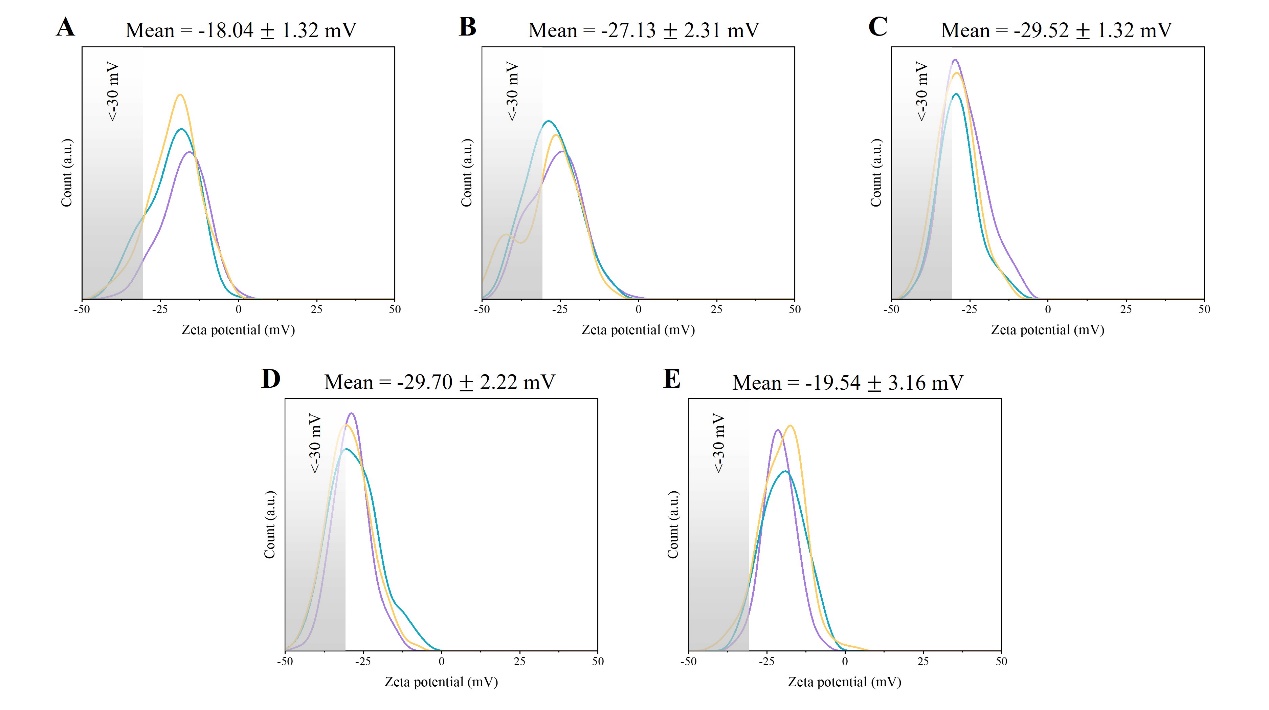
*

**Figure S26.** Zeta potential results of CNC obtained through different enzymatic hydrolysis condition: A) 3 h+21 h, B) 6 h+18 h, C) 9 h+15 h, D) 12 h+12 h, E) 24 h.


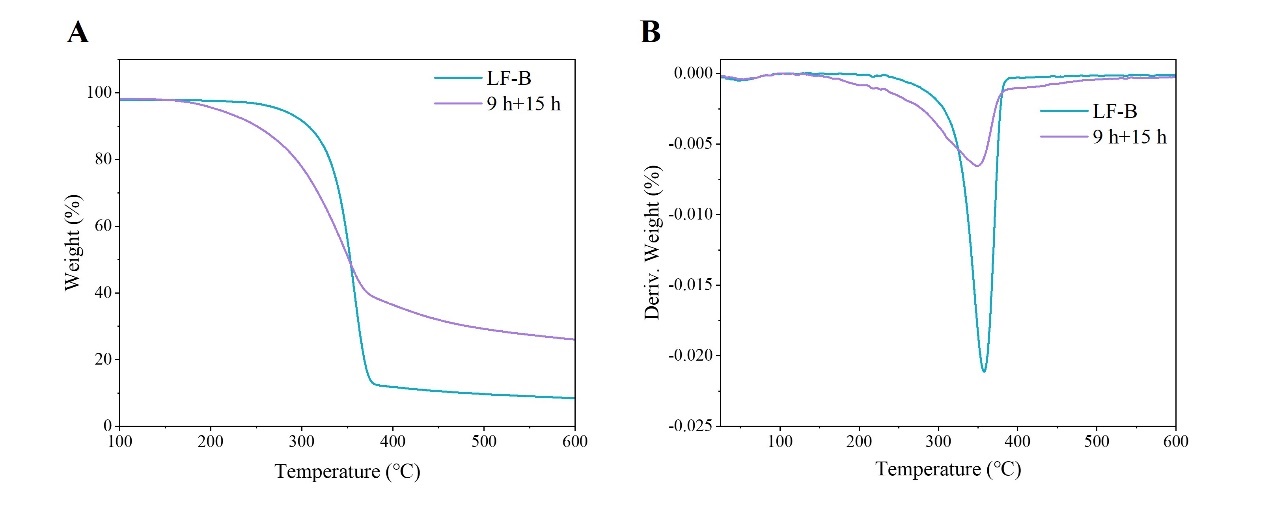


**Figure S27.** TG (A) and DTG (B) results of bleached steam-exploded corn stover long fibers (LF-B) and CNC prepared by 9 h+15 h enzymatic hydrolysis (9h+15h).


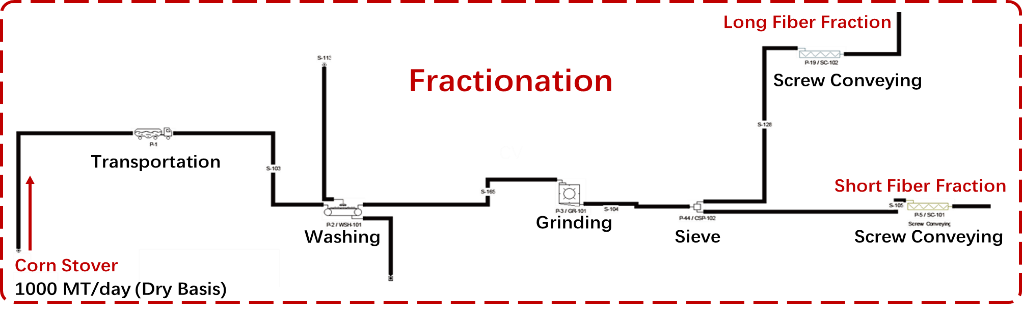


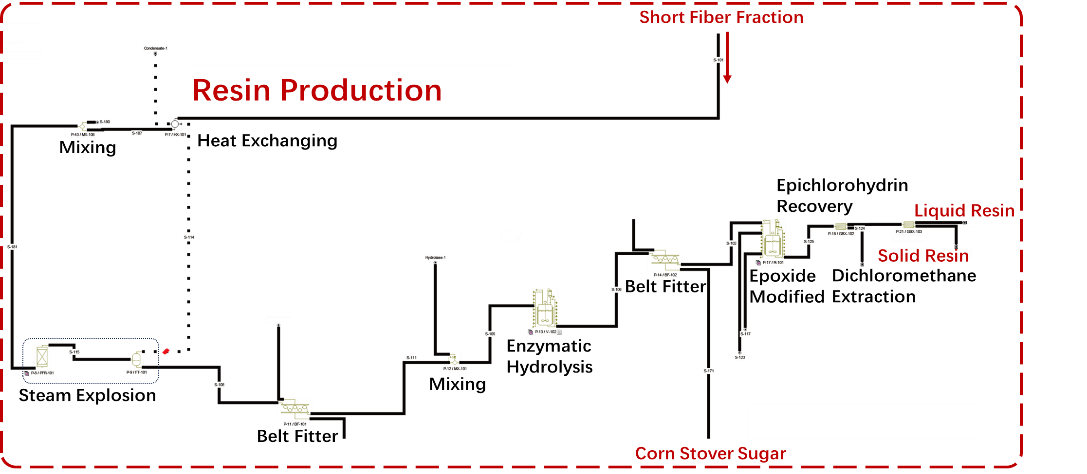


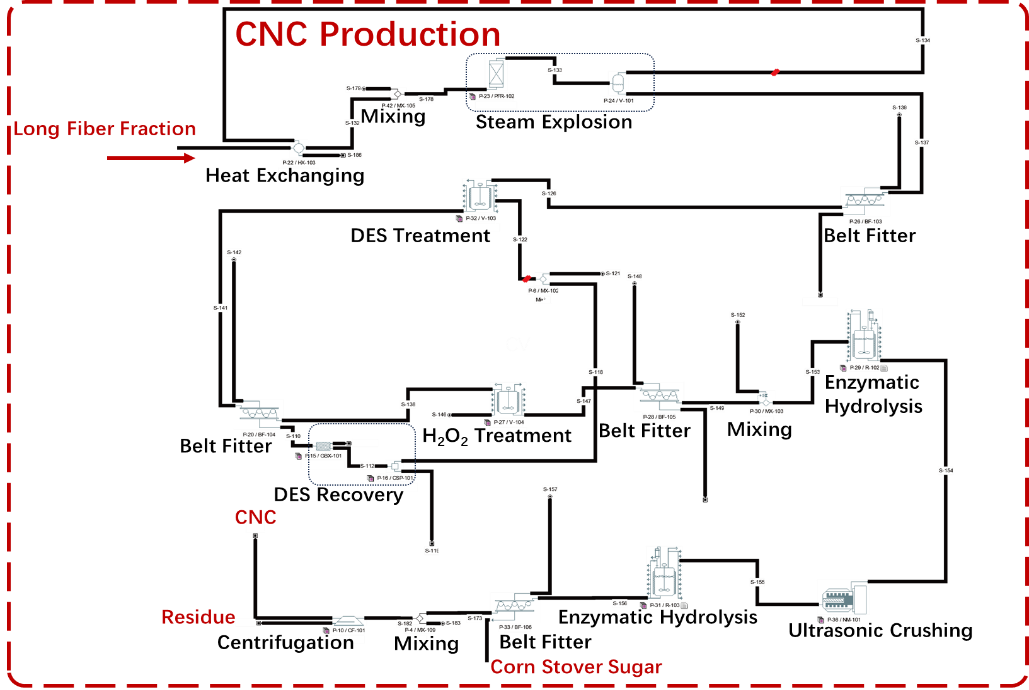


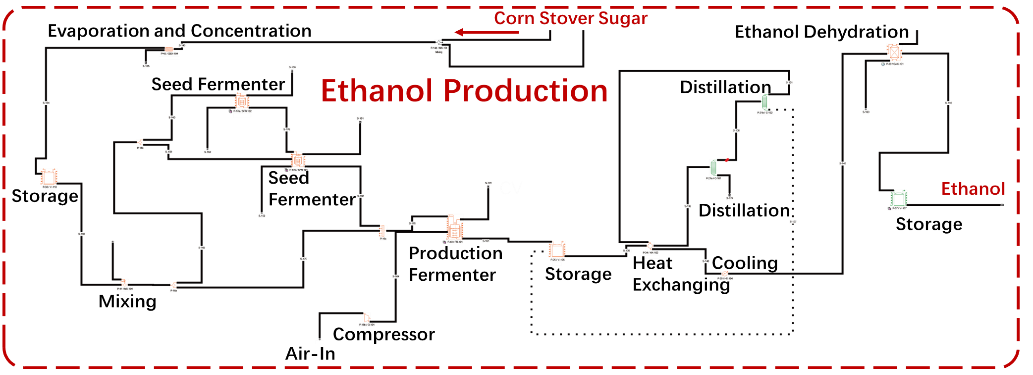


**Figure S28.** Process route diagram for the co-production of lignin-based resin, CNC and bioethanol production from corn stover (generated by SuperPro Designer software).


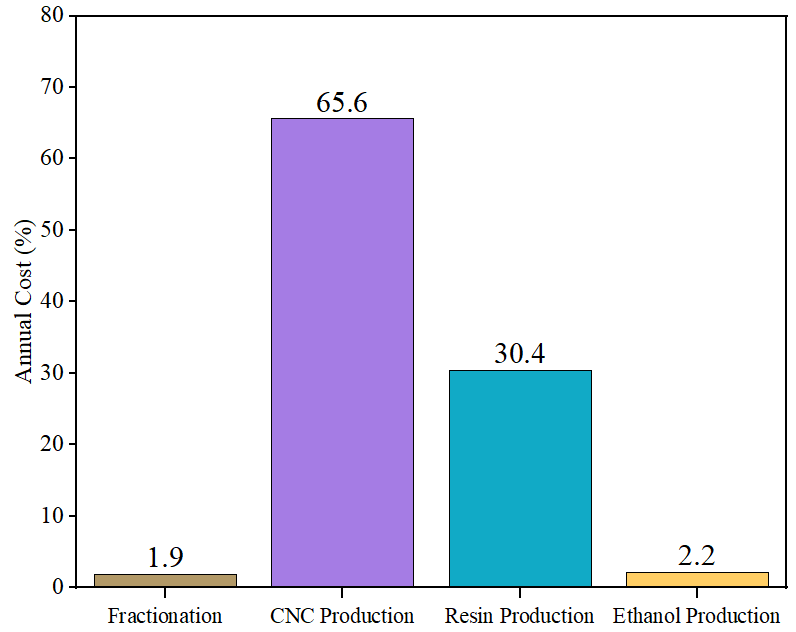


**Figure S29.** Annual operating cost of each section in the production line for lignocellulosic high-value material co-production bioethanol consuming 330,000 tons of corn stover annually.


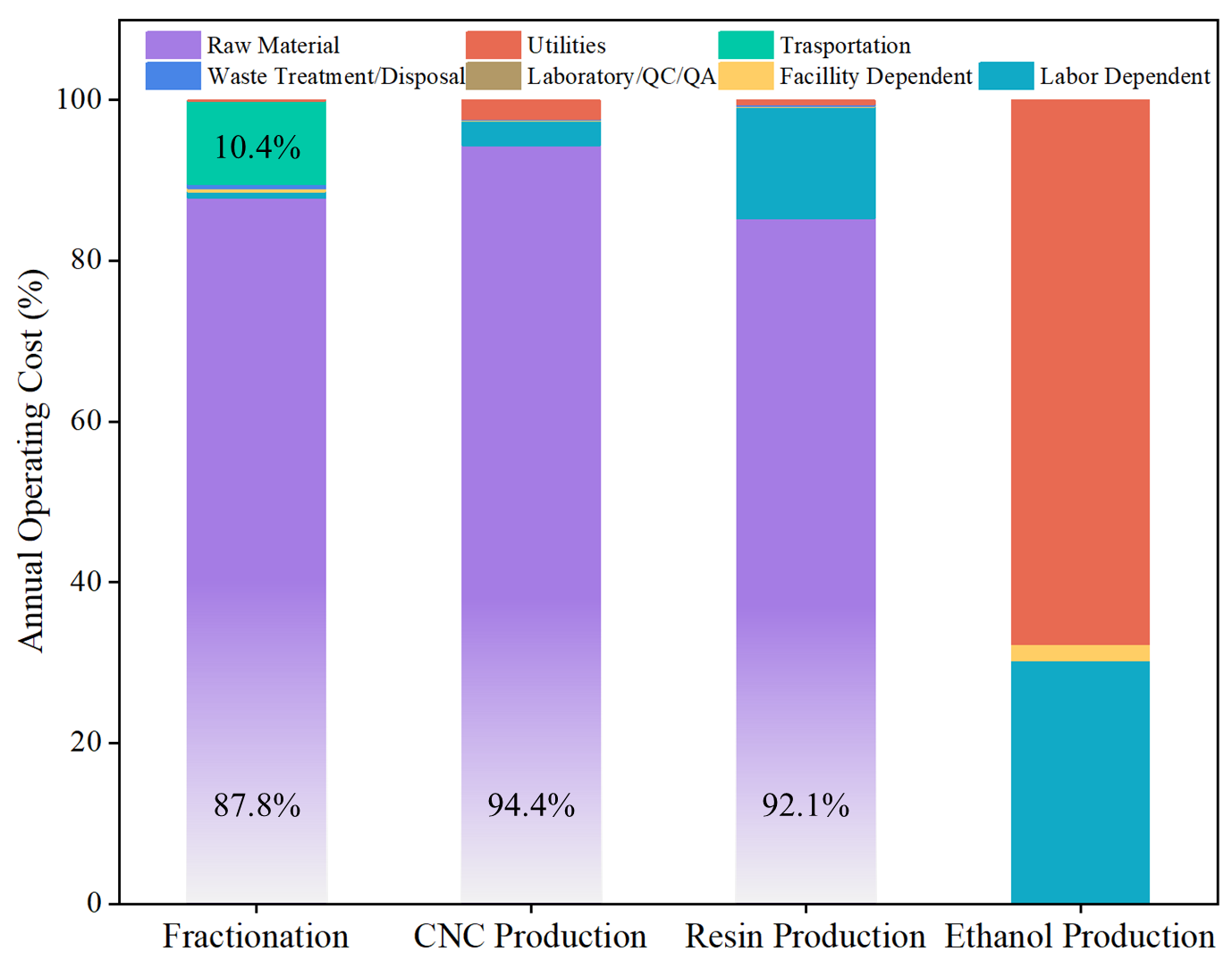


**Figure S30.** Annual cost of each section in the production line for lignocellulosic high-value material co-production bioethanol consuming 330,000 tons of corn stover annually.


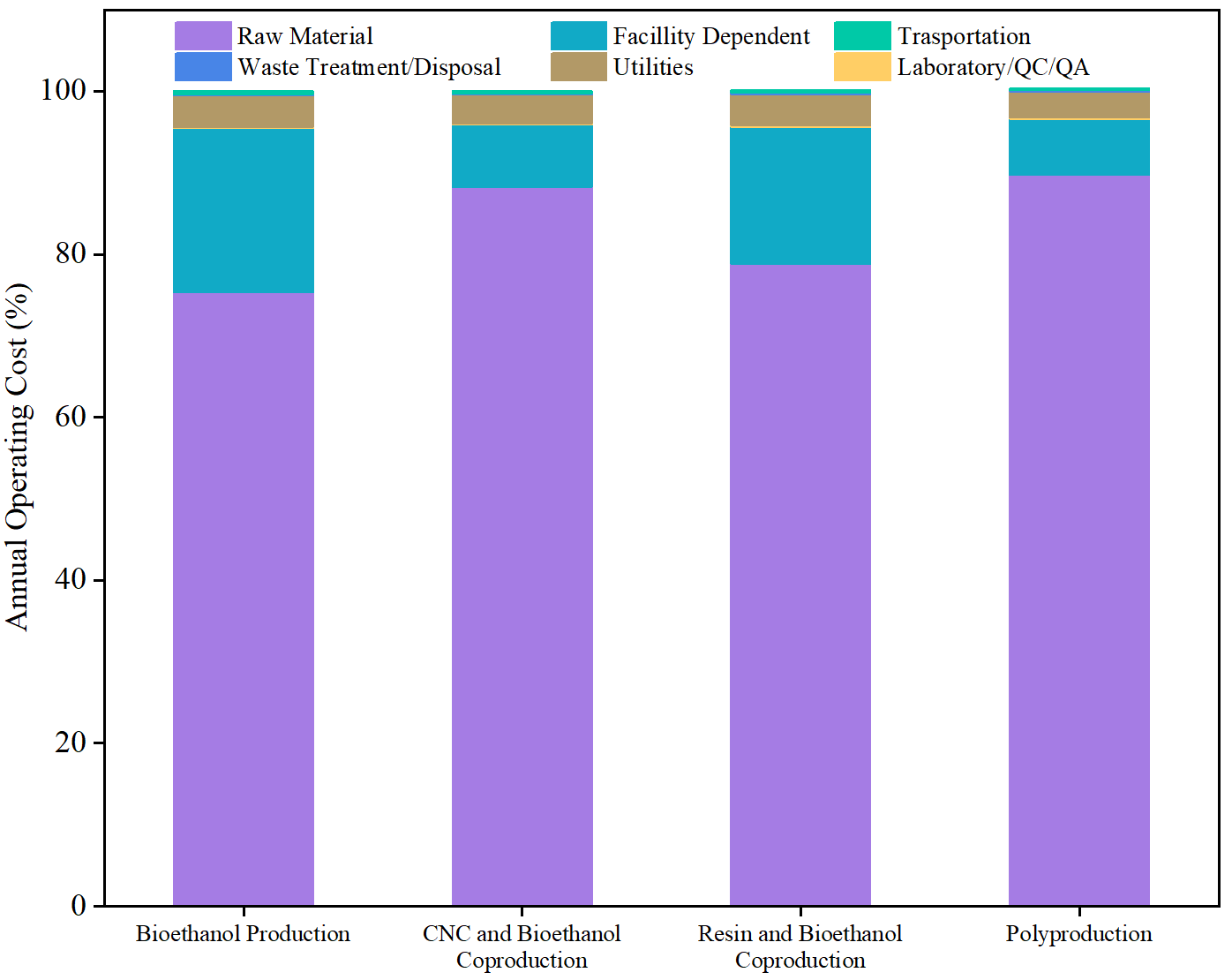


**Figure S31.** Percentage of annual operating costs under the bioethanol production, CNC and bioethanol coproduction, lignin-based epoxy resin and bioethanol coproduction, and poly-production process routes.


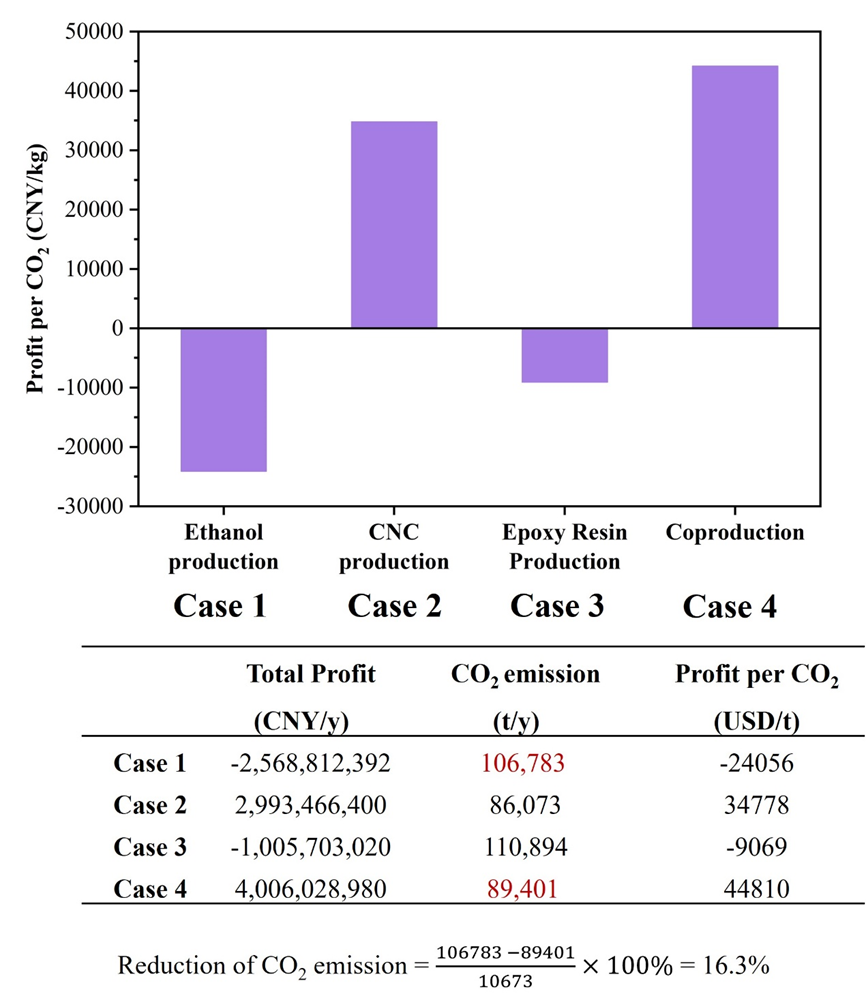


**Figure S32.** Profit per unit CO_2_ emission for ethanol-only production, CNC production, lignin-based epoxy resin production, and co-production process.

*
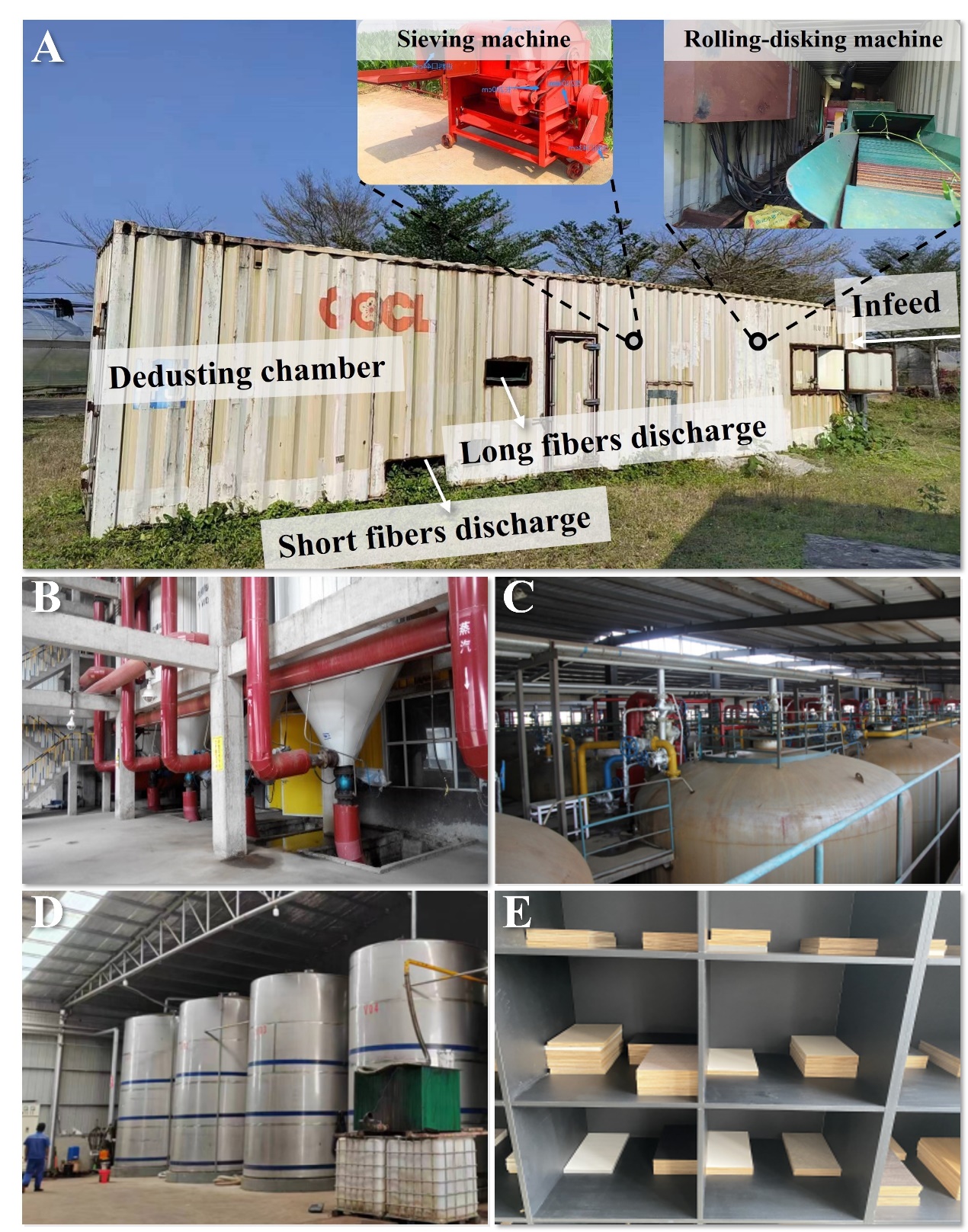
*

**Figure S33.** An industrial-scale precision biorefinery line for corn stover (300,000 t/a). A) Containerized mechanical fractionation system. B) Low-intensity methanol-protected steam explosion. C) CQD-enhanced high-solids enzymatic hydrolysis. D) EHL-based adhesive production line. E) Plywood bonded with EHL-based adhesive.

EHL, Enzyme-hydrolyzed lignin.


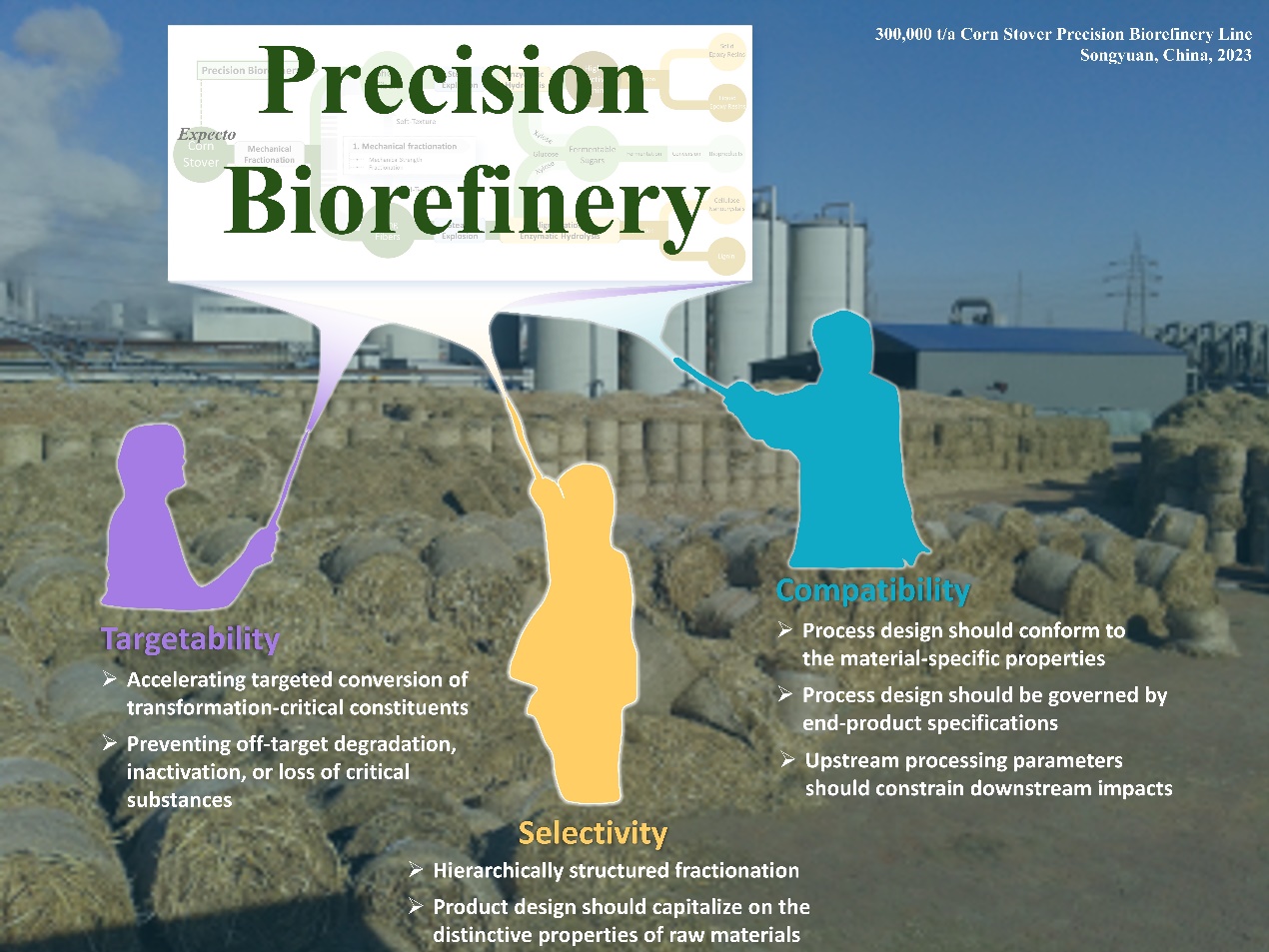


**Figure S34.** Three principles of precision biorefinery.

**Supplementary Tables**

**Table S1.** Comparison of molecular weight distribution of steam-exploded (1 min) corn stover (SE1) and steam-exploded (1 min) corn stover long fibers (SE1-L) and short fibers (SE1-S).

| Sample | Mn | Mw | PDI |
| --- | --- | --- | --- |
| SE1 | 7373.53 | 21880.56 | 2.97 |
| SE1-S | 7853.90 | 20777.74 | 2.65 |
| SE1-L | 10114.59 | 24060.93 | 2.38 |

SE1, Enzyme-hydrolyzed lignin from 1-min steam-exploded corn stover.

SE1-S, Enzyme-hydrolyzed lignin from short fibers of 1-min steam-exploded corn stover.

SE1-L, Enzyme-hydrolyzed lignin from long fibers of 1-min steam-exploded corn stover.

Mn, Number-average molecular weight.

Mw, Weight-average molecular weight.

PDI, Polydispersity index.

PDI = Mw/Mn.

**Table S2.** Comparison of semi-quantitative analysis results of EHL before and after steam explosion and fractionation treatments.

| Sample | A_3340_/A_1512_ | A_2923_/A_1512_ | A_1702_/A_1512_ | A_1363_/A_1512_ | A_1125_/A_1512_ |
| --- | --- | --- | --- | --- | --- |
| SE1 | 1.14 | 0.90 | 0.89 | 0.92 | 1.12 |
| SE1-S | 1.13 | 0.91 | 0.86 | 0.82 | 1.32 |
| SE1-L | 0.95 | 0.77 | 0.81 | 0.79 | 1.10 |

SE1, Enzyme-hydrolyzed lignin from 1-min steam-exploded corn stover.

SE1-S, Enzyme-hydrolyzed lignin from short fibers of 1-min steam-exploded corn stover.

SE1-L, Enzyme-hydrolyzed lignin from long fibers of 1-min steam-exploded corn stover.

**Table S3.** Comparison of molecular weight distribution of lignin samples at different steam explosion intensities.

| Sample | Mn | Mw | PDI |
| --- | --- | --- | --- |
| EHL | 7249.60 | 22374.35 | 3.09 |
| SE1 | 7373.53 | 21880.56 | 2.97 |
| SE10 | 5509.29 | 18936.75 | 3.44 |

EHL, Enzyme-hydrolyzed Lignin.

SE1, Enzyme-hydrolyzed lignin from 1-min steam-exploded corn stover.

SE10, Enzyme-hydrolyzed lignin from 10-min steam-exploded corn stover.

Mn, Number-average molecular weight.

Mw, Weight-average molecular weight.

PDI, Polydispersity index.

PDI = Mw/Mn.

**Table S4.** Comparison of semi-quantitative analysis results of enzymatic lignin FTIR spectra at different steam explosion intensities.

| Sample | A_3340_/A_1512_ | A_2923_/A_1512_ | A_1702_/A_1512_ | A_1363_/A_1512_ | A_1125_/A_1512_ |
| --- | --- | --- | --- | --- | --- |
| EHL | 1.45 | 0.64 | 0.80 | 0.78 | 1.34 |
| SE1 | 1.14 | 0.90 | 0.89 | 0.92 | 1.12 |
| SE10 | 0.96 | 0.67 | 0.78 | 0.67 | 1.00 |

EHL, Enzyme-hydrolyzed Lignin.

SE1, Enzyme-hydrolyzed lignin from 1-min steam-exploded corn stover.

SE10, Enzyme-hydrolyzed lignin from 10-min steam-exploded corn stover.

**Table S5.** Comparison of molecular weight distribution of lignin samples at different steam explosion intensities.

| Sample | Mn | Mw | PDI |
| --- | --- | --- | --- |
| SE1-S | 7853.90 | 20777.74 | 2.65 |
| MSE1-S | 12082.00 | 22852.43 | 1.89 |

SE1-S, Enzyme-hydrolyzed lignin from short fibers of 1-min steam-exploded corn stover.

MSE1-S, Enzyme-hydrolyzed lignin from short fibers of 1-min methanol-protected steam-exploded corn stover.

Mn, Number-average molecular weight.

Mw, Weight-average molecular weight.

PDI, Polydispersity index.

PDI = Mw/Mn.

**Table S6.** Comparative analysis of reaction products from methanol acting on lignocellulose under different conditions.

| Nucleo-philicity | Reactants and catalysts | Conditions | Products | Ref |
| --- | --- | --- | --- | --- |
| High | Extractives-free corn stover (**2g**) + methanol (**100 mL**) + H_2_WO_4_ (0.5 g) | 250 mL autoclave reactor, **~200℃**, 6 h, 600 rpm | Lignin oligomers and its derivatives | ^[6]^ |
| High | Ethanol-benzene treated birch sawdust (**2.0 g**) + methanol (**40 mL**) + Ni/C catalyst (0.10 g) | 75 mL autoclave reactor, **200℃**, 6 h, 500 rpm | Lignin oligomers | ^[7]^ |
| High | Lignin (**0.1 g**) + methanol (**10 mL**) + H_2_O (10 mL) + Pd-Zn/AC-10 catalyst (0.1 g) | 50 mL autoclave reactor, **250℃**, 4 h | Lignin oligomers | ^[8]^ |
| Low | 40 mesh raw poplar powder (**2 g**) + methanol (**20 mL**) + 0.045 N H_2_SO_4_ (20 mL) + 37% aqueous formaldehyde (4 mL) | 75 mL Parr reactor, **160℃**, 4 h, 12 bar N_2,_ 700 rpm | Macro-  molecular lignin | ^[9]^ |
| Low | 40 mesh raw corn stover  (**200 g**) + methanol (**40 mL**) + H_2_O (360 g) | 2 L steam explosion reactor, **160℃**, 5 min, 1.0 MPa (near-saturated steam) | Macro-molecular lignin | This work |

**Table S7.** Process steps of the bioethanol only production, CNC and bioethanol production, lignin-based epoxy resin and bioethanol production, and coproduction process routes.

| Routes | Process steps |
| --- | --- |
| Ethanol Only Production | - Fractionation:   Transportation, washing, and crushing   - Steam explosion pretreatment:   Steam explosion pressure at 1.0 MPa for 10 minutes   - Enzymatic hydrolysis:   Temperature at 50 °C of 96 hours, solid content at 10%, enzyme dosage at 20 FPU/g DM, stirring speed at 150 rpm   - Ethanol production:   The hydrolysate is concentrated and then directly enters the ethanol production |
| CNC and Ethanol Production | - Mechanical texture sorting:   Transportation, washing, disc rolling and sieving.   - Steam explosion pretreatment:   Long fibers were rehydrated (2:1 water-to-fibers ratio), steam-exploded (1.0 MPa, 10 min), DES delignified, and H_2_O_2_ bleached.   - Enzymatic hydrolysis:   Temperature at 50 °C of 96 hours, solid content at 10%, enzyme dosage at 20 FPU/g DM, stirring speed at 150 rpm, with 1 ppm carbon quantum dots added to enhance efficiency   - CNC production   Bleached long fibers were two-stage enzymatically hydrolyzed (50 °C, 9 h, 10% solids, 20 FPU/g DM, 150 rpm), ultrasonicated (800 W, 30 min), and subjected to a secondary hydrolysis (15 h, no additional enzymes) to yield CNC.   - Ethanol production:   The remaining non-materialized long fibers raw materials from corn stover are converted under the same enzymatic hydrolysis conditions as the process route that only produces bioethanol, and then enter the ethanol production stage |
| Epoxy Resin and Ethanol Production | - Mechanical texture sorting:   Transportation, washing, disc rolling and sieving.   - Methanol protected Steam explosion pretreatment:   short fibers were mixed with a 10% methanol solution (2:1 mass ratio) and steam-exploded at 1.0 MPa for 1 min, achieving partial hydrolysis (5% cellulose, 10% hemicellulose)   - CQD enhanced Enzymatic hydrolysis:   Temperature at 50 °C of 96 hours, solid content at 10%, enzyme dosage at 20 FPU/g DM, stirring speed at 150 rpm, with 1 ppm carbon quantum dots added to enhance efficiency   - Epoxy Resin production   The solids were separated via plate-frame filtration and reacted with sodium hydroxide, epichlorohydrin, and aniline (98 °C, 1.5 h) to synthesize epoxy resin, while liquids were directed to fermentation.   - Ethanol production:   The remaining non-materialized short fibers raw materials from corn stover are converted under the same enzymatic hydrolysis conditions as the process route that only produces bioethanol, and then enter the ethanol production stage. |
| Coproduction Route | - Mechanical texture sorting:   Transportation, washing, disc rolling and sieving.   - Methanol protected Steam explosion for short fibers:   Short fibers were mixed with a 10% methanol solution (2:1 mass ratio) and steam-exploded at 1.0 MPa for 1 min, achieving partial hydrolysis (5% cellulose, 10% hemicellulose)   - Steam explosion for long fibers:   Long fibers were rehydrated (2:1 water-to-fibers ratio), steam-exploded (1.0 MPa, 10 min), DES delignified, and H_2_O_2_ bleached.   - CQD enhanced Enzymatic hydrolysis for short fibers:   Temperature at 50 °C of 96 hours, solid content at 10%, enzyme dosage at 20 FPU/g DM, stirring speed at 150 rpm, with 1 ppm carbon quantum dots added to enhance efficiency   - Enzymatic hydrolysis for long fibers:   Temperature at 50 °C of 96 hours, solid content at 10%, enzyme dosage at 20 FPU/g DM, stirring speed at 150 rpm, with 1 ppm carbon quantum dots added to enhance efficiency   - Epoxy Resin production   The solids were separated via plate-frame filtration and reacted with sodium hydroxide, epichlorohydrin, and aniline (98 °C, 1.5 h) to synthesize epoxy resin, while liquids were directed to fermentation.   - CNC production   Bleached long fibers were two-stage enzymatically hydrolyzed (50 °C, 9 h, 10% solids, 20 FPU/g DM, 150 rpm), ultrasonicated (800 W, 30 min), and subjected to a secondary hydrolysis (15 h, no additional enzymes) to yield CNC.   - Ethanol production:   The remaining non-materialized short fibers raw materials from corn stover are converted under the same enzymatic hydrolysis conditions as the process route that only produces bioethanol, and then enter the ethanol production stage. |

**Table S8.** Costs involved in the model.

| Utility costs involved in the model | |
| --- | --- |
| Utility | Cost (USD) |
| Standard Power | 0.091 (USD /kW^-^h) |
| Steam | 16.70 (USD /MT) |
| Steam (High Pressure) | 18.10 (USD /MT) |
| Water | 0.42 (USD /MT) |
|  | |
| **Equipment costs involved in the model** | |
| Equipment | Cost (USD) |
| Belt Filter | 98,000.00 |
| Blending Tank | 2,000.00 |
| Centrifugal Compressor | 17,000.00 |
| Centritech Centrifuge | 3,000.00 |
| Component Splitter | 49,000.00 |
| Distillation Column | 42,000.00 |
| Fermentor | 210,000.00 |
| Flat Bottom Tank | 630,000.00 |
|  | |
| **Equipment costs involved in the model** | |
| Equipment | Cost (USD) |
| Granular Activated Carbon Column | 42,000.00 |
| Grinder | 25,000.00 |
| Heat Exchanger | 6,000.00 |
| Plug Flow Reactor | 1,000.00 |
| Screw Conveyor | 35,000.00 |
| Seed Fermentor | 70,000.00 |
| Stirred Reactor | 119,000.00 |
| Stirred Reactor (Small Size) | 2,000.00 |
| Ultrasound Crusher | 2,000.00 |
| Washer | 42,000.00 |
|  | |
| **Raw material costs involved in the model** | |
| Raw Materials Cost (USD/MT) | Raw Materials Cost (USD/MT) |
| Amm. Sulfate | 100.00 |
| Aniline | 1,564.00 |
| Corn Stover | 41.82 |
| Carbon Quantum Dots (CQDs) | 156,130,000.00 |
| Deep Eutectic Solvent (DES) | 1,256.00 |
| Enzyme | 4,180.00 |
| Epichlorohydrin | 1,131.87 |
| Sodium Hydroxide | 347.50 |
| Hydro Peroxide | 25.11 |
| Methanol | 343.10 |

MT, Metric Ton; CNY, Chinese Yuan; USD, United States Dollar.

**Table S9.** Comparison of product cost and profitability under different process routes.

|  | **Case 1** | **Case 2** | **Case 3** | **Case 4** |
| --- | --- | --- | --- | --- |
|  | **Bioethanol only** | **Bioethanol and CNC** | **Bioethanol and resins** | **Coproduction** |
| Ethanol production (MT/yr) | 76924.93 | 61439 | 79849 | 63786 |
| Ethanol price (CNY/MT) | 5600 | 5600 | 5600 | 5600 |
| Ethanol revenue (CNY/yr） | 430779608 | 344058400 | 447154400 | 357201600 |
| CNC production (MT/yr) | 0 | 9852.24 | 0 | 9852.24 |
| CNC price (CNY/MT) | 800000 | 800000 | 800000 | 800000 |
| CNC revenue (CNY/yr） | 0 | 7881792000 | 0 | 7881792000 |
| Liquid resin production (MT/yr) | 0 | 0 | 55940.21 | 55940.21 |
| Liquid resin price (CNY/MT) | 16000 | 16000 | 16000 | 16000 |
| Liquid resin revenue (CNY/yr） | 0 | 0 | 895043360 | 895043360 |
| Solid resin production (MT/yr) | 0 | 0 | 49762.76 | 49762.76 |
| Solid resin price (CNY/MT) | 14500 | 14500 | 14500 | 14500 |
| Solid resin revenue (CNY/yr） | 0 | 0 | 721560020 | 721560020 |
| Total revenue (CNY/yr） | 430779608 | 8225850400 | 2063757780 | 9855596980 |
| Total cost (CNY/yr） | 2999592000 | 5232384000 | 3069460800 | 5849568000 |
| Total profit (CNY/yr） | -2568812392 | 2993466400 | -1005703020 | 4006028980 |

The price of **ethanol** (99.7%) was set at 5,600 CNY/MT. Based on the fluctuation analysis of China’s ethanol market from September 2024 to August 2025, with a maximum value of 5,965 CNY/MT and a minimum value of 5,105 CNY/MT (https://www.100ppi.com/news/detail-20250904-4731655.html), a price of 5,600 CNY/MT is therefore reasonable.

The price of **CNC** (oven-dry) was set at 800,000 CNY/MT. For reference, the price of a comparable product (CelluRods) from CelluForce (USA, https://celluforce.com/product/cellurods-100l/) is 175 USD/20 L (7.5% w/w), which converts to 840,000 CNY/MT on an oven-dry basis using an exchange rate of 7.2. The price of a similar product (BGG Ultra CNC) from Blue Goose Biorefineries (Canada, https://store.bluegoosebiorefineries.com/#tile-products-opd5wX) is 1,000 USD/kg on a oven-dry basis, equivalent to 7,200,000 CNY/MT. The price of a comparable product (NFC-33L2) from Shengquan Group (China, https://detail.1688.com/offer/672478658606.html) is 90 CNY/kg (8% w/w), corresponding to an oven-dry price of 1,125,000 CNY/MT. Based on the comprehensive review of the above product quotations, we have set the price of CNC at 800,000 CNY/MT.

The price of **liquid epoxy resin** was set at 16000 CNY/MT, with reference to quotations from Baozhen Chemistry (16000 CNY/MT, https://www.rawmex.cn/sell-4198779.html) and Shandong Hanbang Scientific Instrument Co., Ltd. (16000 CNY/MT, <http://www.chemct.cn/info/detail-20250905-4735931.html>).

The price of **solid epoxy resin** was set at 14500 CNY/MT, with reference to E-12 (13600-16000 CNY/MT, https://detail.1688.com/offer/37032507469.html).

MT: Metric Ton. CNY: Chinese Yuan. USD: U.S. Dollar. CNC: Cellulose Nanocrystals.

**Table S10. Comparison of total revenues and growth rate under different process routes.**

|  | **Unregulated process (Baseline)** | **CNC-regulated**  **process** | **Epoxy Resin-regulated**  **process** | **Co-regulated**  **process** |
| --- | --- | --- | --- | --- |
| Ethanol production (MT/yr) | 62486.29 | 61504.19 | 64106.53 | 63786.00 |
| Ethanol price (CNY/MT) | 5600 | 5600 | 5600 | 5600 |
| Ethanol revenue (CNY/yr） | 349923197.49 | 344423488.57 | 358996582.91 | 357201600.00 |
| CNC production (MT/yr) | 6799.34 | 9852.24 | 6799.34 | 9852.24 |
| CNC price (CNY/MT) | 800000 | 800000 | 800000 | 800000 |
| CNC revenue (CNY/yr） | 5439469979 | 7881792000 | 5439469979 | 7881792000 |
| Liquid resin production (MT/yr) | 37718.43 | 37718.43 | 55940.21 | 55940.21 |
| Liquid resin price (CNY/MT) | 16000 | 16000 | 16000 | 16000 |
| Liquid resin revenue (CNY/yr） | 603494949.77 | 603494949.77 | 895043360.00 | 895043360.00 |
| Solid resin production (MT/yr) | 56292.71 | 56292.71 | 49762.76 | 49762.76 |
| Solid resin price (CNY/MT) | 14500 | 14500 | 14500 | 14500 |
| Solid resin revenue (CNY/yr） | 816244366.5 | 816244366.5 | 721560020 | 721560020 |
| Total revenue (CNY/yr） | 7209132493 | 9645954805 | 7415069942 | 9855596980 |
| Revenue increasement (CNY/yr） | NA | 2436822312 | 205937449.1 | 2646464487 |
| Revenue growth rate (%) | NA | 33.80 | 2.86 | 36.71 |
| Ethanol contribution (%) | NA | -0.08 | 0.13 | 0.10 |
| CNC contribution (%) | NA | 33.88 | 0.00 | 33.88 |
| Liquid resin contribution (%) | NA | 0.00 | 4.04 | 4.04 |
| Solid resin contribution (%) | NA | 0.00 | -1.31 | -1.31 |

**Unregulated process**: For long fibers, 24 h enzymatic hydrolysis followed by ultrasonication was employed. For short fibers, methanol was not added during steam explosion, and CQDs were not supplemented during enzymatic hydrolysis.

**CNC-regulated process**: For long fibers, 9h + 15 h ultrasonication-assisted enzymatic hydrolysis was employed. For short fibers, methanol was not added during steam explosion, and CQDs were not supplemented during enzymatic hydrolysis.

**Epoxy resin-regulated process**: For long fibers, 9h + 15 h ultrasonication-assisted enzymatic hydrolysis was employed. For short fibers, methanol was added during steam explosion, and CQDs were supplemented during enzymatic hydrolysis.

**Co-regulated process**: For long fibers, 9h + 15 h ultrasonication-assisted enzymatic hydrolysis was employed. For short fibers, methanol was added during steam explosion, and CQDs were supplemented during enzymatic hydrolysis.

MT: Metric Ton.

CNY: Chinese Yuan.

CNC: Cellulose Nanocrystals.

CQD: Carbon Quantum Dots.

**References**

[1] S. Lv, S. Zhang, J. Zuo, et al., "The efficient detection of by sulfonamidated lignin composite carbon quantum dots", *Polymer engineering and science* **2023**, *63*(5), 1439. <https://doi.org/10.1002/pen.26295>.

[2] L. Zhu, D. Shen, Q. Wang, K. H. Luo, "Green Synthesis of Tunable Fluorescent Carbon Quantum Dots from Lignin and Their Application in Anti-Counterfeit Printing", *ACS applied materials & interfaces* **2021**, *13*(47), 56465. <https://doi.org/10.1021/acsami.1c16679>.

[3] H.-M. Wang, C.-Y. Ma, H.-Y. Li, et al., "Structural Variations of Lignin Macromolecules from Early Growth Stages of Poplar Cell Walls", *ACS sustainable chemistry & engineering* **2020**, *8*(4), 1813. <https://doi.org/10.1021/acssuschemeng.9b05845>.

[4] P. Verdross, S. Guinchard, R. T. Woodward, A. Bismarck, "Black liquor-based epoxy resin: Thermosets from untreated kraft lignin", *Chemical Engineering Journal* **2023**, *475*145787. <https://doi.org/10.1016/j.cej.2023.145787>.

[5] Determination of structural carbohydrates and lignin in biomass: laboratory analytical procedure (LAP) : issue date, 4/25/2008. National Renewable Energy Laboratory: Golden, Colo, **2008**; Vol. NREL/TP-510-42618;510-42618;.

[6] Z. Ma, S. Kasipandi, Z. Wen, et al., "Highly efficient fractionation of corn stover into lignin monomers and cellulose-rich pulp over H_2_WO_4_", *Applied Catalysis B: Environmental* **2021**, *284*119731. <https://doi.org/https://doi.org/10.1016/j.apcatb.2020.119731>.

[7] Q. Song, F. Wang, J. Cai, et al., "Lignin depolymerization (LDP) in alcohol over nickel-based catalysts via a fragmentation–hydrogenolysis process", *Energy & Environmental Science* **2013**, *6*(3), 994. <https://doi.org/10.1039/C2EE23741E>.

[8] J. Liang, K. Wu, F. Li, et al., "Hydrogenolysis of lignin with endogenous hydrogen from aqueous phase reforming of methanol", *Industrial Crops and Products* **2024**, *217*118860. <https://doi.org/https://doi.org/10.1016/j.indcrop.2024.118860>.

[9] H. Luo, M. M. Abu-Omar, "Lignin extraction and catalytic upgrading from genetically modified poplar", *Green Chemistry* **2018**, *20*(3), 745. <https://doi.org/10.1039/C7GC03417B>.
